# Functional and structural characterization of a serine protease from *Bacillus toyonensis* co-expressed with short peptides from late embryogenesis abundant (LEA) proteins

**DOI:** 10.1128/spectrum.03010-25

**Published:** 2026-06-22

**Authors:** Ammar Khazaal Kadhim Almansoori, Nurul Ain Syukriyah, Choo Yi Hang, Mustafa Abdulfattah, Shinya Ikeno, Lo Hui Yu, Haider Mahmod Jasim, Rashidah Abdul Rahim

**Affiliations:** 1 School of Biological Sciences, Universiti Sains Malaysia, Gelugor, Penang, Malaysia; 2 Centre for Chemical Biology (CCB), Universiti Sains Malaysia, Bayan Lepas, Penang, Malaysia; 3 Department of Medical Laboratory Techniques, Al-Mustaqbal University 588508, Hillah, Babylon, Iraq; 4 Department of Biological Functions and Engineering, Graduate School of Life Science and System Engineering, Kyushu Institute of Technology 12924, Kitakyushu, Japan; 5 College of Pharmacy, Al-Muthanna University 362927, Samawah, Iraq; Ocean University of China, Qingdao, China

**Keywords:** serine protease, *Bacillus toyonensis*, LEA-derived short peptides, enzyme characterization, structural properties

## Abstract

The increasing demand for high-performance proteases in industrial applications necessitates strategies to enhance enzyme yield, stability, and functional robustness under extreme conditions. This study investigates a serine protease from *Bacillus toyonensis*, isolated from fermented kitchen waste, and its co-expression with short peptides derived from late embryogenesis abundant (LEA) proteins, LEA-I, and LEA-K. The protease gene was cloned into a pRSFDuet-1 vector and expressed in *Escherichia coli* BL21(DE3), followed by purification using immobilized metal affinity chromatography. Co-expression with LEA peptides significantly enhanced protease production and stability, with ProLEA-K achieving the highest purification fold (1,638.00) and specific activity (16.38 U/mg) compared to 0.01 U/mg in the crude extract. Functionally, ProLEA-K exhibited a 2.5-fold increase in activity at 60°C and retained substantial activity up to 70°C, whereas the protease expressed alone (ProWL) declined sharply at elevated temperatures and maintained high activity across a broad pH range spanning acidic to alkaline conditions, a rare feature for acidic serine proteases. Evaluation under detergent-containing conditions revealed a differential response, whereby detergents enhanced the activity of ProWL but reduced the activity of LEA-co-expressed variants, indicating a context-dependent effect of LEA peptides on enzyme performance. *In silico* analysis supported these experimental findings. Multiple sequence alignment revealed strong conservation of the catalytic triad (Asp171, His203, and Ser225) and an amino acid profile consistent with secreted bacterial enzymes. Structural modeling confirmed stable folding, and molecular docking with azocasein indicated stronger substrate binding in LEA-containing variants (−6.9 kcal/mol for ProLEA-K vs −5.8 kcal/mol for ProWL). These results identify LEA peptides, particularly LEA-K, as effective molecular stabilizers, improving both the yield and functional robustness of industrial proteases.

**IMPORTANCE:**

This work highlights a simple and effective approach to improve industrial proteases using short late embryogenesis abundant peptides as natural stabilizers. The method enhanced yield, thermal stability, and pH adaptability across acidic and alkaline conditions without genetic redesign of the protease itself, offering a broadly applicable and economical strategy for enzyme improvement.

## INTRODUCTION

Proteases are well-known specialized hydrolases that catalyze the breaking of peptide bonds in a protein sequence through hydrolysis (1). Their classification is determined by factors such as optimal pH range, specific cleavage site, and underlying catalytic mechanism (2–4). Subsequently, serine proteases are categorized as alkaline proteases, which function optimally within a pH range of 7.0–11.0 (5, 6). Their catalytic mechanism depends on a highly reactive serine residue within the catalytic triad along with histidine and aspartate (7–9). Several microorganisms are known to synthesize serine protease, including *Neisseria*, *Shigella*, *Arthrobacter*, *Streptomyces*, *Flavobacterium*, *Aeromonas*, *Escherichia*, and *Bacillus* species (9–14), with *Bacillus* sp. being recognized as one of the most common organisms to produce the enzyme.

Most *Bacillus*-derived serine proteases (EC 3.4.21) typically comprise around 442 amino acids, with molecular weights between 27 and 71 kDa. These enzymes generally exhibit optimal activity within an alkaline pH range of 8–12 and at temperatures from 37°C to 70°C (15–18). On the other hand, *Bacillus toyonensis*, which was discovered in 1966 in Japan, is well-known for its probiotic capability. According to Kumar et al. (19), the optimum temperature of serine protease isolated from *B. toyonensis* is 55°C, along with an optimum pH at 7–8. Due to its probiotic potential and promising enzymatic profile, *B. toyonensis* has gathered interest for recombinant serine protease production. Hence, protein engineering strategies have been extensively employed to enhance the stability and catalytic performance of the enzyme, which aims to support more sustainable and efficient industrial applications.

In relation to this matter, the late embryogenesis abundant (LEA) protein, which is a primary metabolite present during the late stages of seed development in plants, was first identified in cotton cotyledons (20). As illustrated by Hong-Bo et al. (21), the LEA proteins are responsible for providing fundamental protection to the host plants against desiccation and various abiotic stresses. Moreover, the proteins were not only discovered in plants, but also found in bacteria (22, 23), fungi, algae, and invertebrates (24–26), whereby Group 3 LEA protein is known to enclose the structural properties of a repeating sequence containing 11-amino acid residues (27). Subsequently, the LEA-like peptides that were derived from the LEA proteins in a sleeping chironomid, *Polypedilum vanderplanki*, were engineered by Ikeno and Haruyama (28) to evaluate the effectiveness of their co-expression in enhancing the production of the green fluorescence protein (GFP). The study demonstrated that with the aid of LEA-like peptides, specifically LEA-II, the expression of the target protein was elevated twofold using the co-expression strategy.

Additionally, the mutated LEA peptides were constructed by Pathak et al. (29) to further examine the enhanced GFP expression level through the co-expression approach, in which the LEA-like peptide variants (LEA-K, LEA-E, LEA-L, and LEA-N) showed a greater efficiency in synthesizing GFP, thereby paving the way for a novel protein engineering blueprint. Beyond this, the LEA-like peptide, in the form of LEA-II, was shown to enhance the expression of Cry protein, an insect-directed toxin extracted from *Bacillus thuringiensis* (30) using the conventional co-expression system. Throughout the study, 0.05 mM isopropyl β-D-1-thiogalactopyranoside (IPTG), along with a 12 h induction time, was optimized to produce threefold of the targeted protein (31). Lactose was also shown to function as an alternative inducer for the expression of the Cry toxin co-expressed with LEA peptides, offering a cost-effective approach compared to conventional IPTG induction (32). The capability of the LEA-like peptide to upregulate the target gene is due to the role of the peptide as a molecular shield, thereby preventing protein aggregation and consequently enhancing the expression of heterologous proteins (33).

Apart from enhanced protein expression, the LEA peptides were investigated for their potential to enhance the physical characteristics of the expression host. A study by Huwaidi et al. (34) revealed that the LEA-I peptide could shield the host cell *Escherichia coli* from ultraviolet radiation damage (UVA and UVC) that aided in the production of reactive oxygen species. Additionally, the LEA-K peptide mutant was proven to elevate the acid tolerance of *E. coli* up to pH 4 (35). With the dynamic nature of the peptide, the co-expression of LEA peptide with *Sphingobacterium* sp. Ab3 lipase further improves the structural properties of the enzyme, in which the expressed enzyme exhibits cold adaptation and operates effectively at neutral pH (5.0–7.0), and shows tolerance toward cofactors such as Na^+^ ions and the water-like solvent methanol, and displays markedly increased activity upon the addition of nonionic detergents (36). In addition, with the aid of the LEA-II mediated co-expression system, the lipase extracted from *B. licheniformis* IBRL-CHS showed enhanced thermostability up to 45°C (37). The capacity of LEA-like peptides to protect target proteins from denaturation may stem from their abundance of polar amino acid residues, which can create physical barriers that prevent direct contact between the biomolecule and the membrane (38).

Although LEA peptides are known for enhancing protein expression and stress tolerance, their impact on the structural and functional properties of serine proteases via co-expression is not well understood. In this study, the effect of Group 3 LEA peptides, specifically LEA-I and LEA-K, on the stability, catalytic efficiency, and yield of a recombinant serine protease from *B. toyonensis* was examined. The enzyme was assessed under stress conditions, including elevated temperature and a wide pH range, with emphasis on acidic conditions. Structural modeling was used to investigate possible stabilizing effects, including hydrogen-bond networks and polar residue interactions that may protect the catalytic triad and maintain enzyme activity under stress.

## MATERIALS AND METHODS

### *In vitro* characterization of serine protease

#### Isolation and identification of protease-producing bacteria

The bacteria were isolated from kitchen waste, including fruit peels, vegetable waste, and eggshells, which had been processed to obtain pure cultures on nutrient agar plates and further confirmed using skim milk agar for protease production. The DNA of the isolated colonies was extracted using the glucose-Tris-EDTA extraction method (39) with slight modifications, and 16S rRNA sequencing was performed to identify the bacterial species (40).

#### Amplification, cloning, and transformation of the protease gene

The serine protease sequence of *Bacillus toyonensis* was obtained from the National Center for Biotechnology Information (NCBI) database (GenBank accession no. AHA07180.1), as illustrated in Fig. S1. Subsequently, a pair of primers was designed using Primer-BLAST, as displayed in Table S1, followed by PCR to amplify the serine protease gene from *B. toyonensis*. Then, the PCR product was purified using the HiYield Gel/PCR DNA Mini Kit (Real Biotech Corporation, Taiwan) to prepare for the subsequent cloning process.

The pRSFLEA-Pro plasmid series was constructed by cloning the serine protease gene into the BamHI and HindIII restriction sites of MCS1 in the pRSF-LEA vector, which also harbors the LEA-like peptide gene inserted between the NdeI and XhoI sites of MCS2 (28), derived from pRSFDuet-1 (Novagen, USA). The vector map, along with the position of the LEA peptide in the MCS2 region and the protease gene in the MCS1 region, is shown in Fig. S2. The recombinant plasmid was subsequently introduced into the expression host *Escherichia coli* BL21(DE3) and grown on LB agar plates supplemented with kanamycin.

#### Expression and purification of recombinant protease

The recombinant protease cultures, denoted as control (ProWL), protease-LEA-I (ProLEA-I), and protease-LEA-K (ProLEA-K), were grown in LB broth with kanamycin at 37°C and 180 rpm until reaching an optical density of 0.4–0.6, then induced with 0.1 M isopropyl β-D-1-thiogalactopyranoside and incubated at 25°C for 18 h at 150 rpm. The expressed cells were harvested by centrifugation at 4°C and 8,000 rpm for 15 min. Then, the expressed cell pellets, which were resuspended using 50 mM Tris-HCl buffer (pH 8.8), were sonicated to break open the cell membrane and release the protein. The expressed proteins were then purified using His-tagged immobilized metal affinity chromatography (IMAC) (41) with TALON disposable column (Takara, USA), followed by protein quantification using the Bradford protein assay (42), with the standard curve shown in Fig. S3.

#### Determination of enzymatic activity

The protease activity was measured using azocasein, a chromogenic casein derivative dyed with sulfanilic acid that releases azo dye upon hydrolysis by protease. Several assay methods were initially evaluated; however, only the azocasein-based method produced consistent and quantifiable results. This assay was slightly modified from the method described by Charney and Tomarelli (43), with one unit of protease activity defined as a 0.01 increase in absorbance at 440 nm within 30 min.

#### Characterization of recombinant protease enzyme

The enzymatic activity of the proteases (ProWL, ProLEA-I, and ProLEA-K) was assessed using the standard azocasein assay, as illustrated by Duarte Rocha et al. (44), with slight modifications. Azocasein (2% in 100 mM Tris-HCl, 0.2 M NaCl) was incubated with crude enzyme (1:1, vol/vol) at 40°C for 30 min. The reaction was stopped with trichloroacetic acid (110 mM), centrifuged (10,000 rpm, 10 min), and the supernatant mixed with 1 M NaOH (1:2, vol/vol). Absorbance was measured at 440 nm (Multiskan GO, ThermoFisher Scientific, Inc.). One unit of activity corresponded to an increase of 0.01 in absorbance at 440 nm in 30 min under the assay conditions. To investigate the effect of temperature, pH, and detergent on protease activity, the proteases were incubated at temperatures ranging from 40°C to 70°C, pH levels from 5 to 11, and with detergents (DMSO, SDS, Tween 20, and Triton X-100) at a concentration of 0.1% for 30 min. For statistical analysis, the significant differences in bacterial growth and protease activity across various experimental conditions were evaluated using two-way ANOVA in GraphPad Prism, with a significance threshold set at *P* value <0.005.

### *In silico* characterization of serine protease

#### Sequence retrieval and homology data set construction

The amino acid sequence of serine protease from *Bacillus toyonensis* was retrieved from the NCBI Protein Database (accessed 2025). Homologous sequences from *Bacillus cereus*, *B. subtilis*, *B. thuringiensis*, *B. wiedmannii*, *B. luti*, and *B. mycoides* were identified using BLASTp. Seventeen sequences with confirmed serine protease function and similar lengths (~263 amino acids) were selected.

#### Multiple sequence alignment and conserved motif analysis

Multiple sequence alignment was performed using Clustal Omega (EMBL-EBI) with default parameters. The alignment was inspected in Jalview to identify conserved regions and the catalytic triad residues Asp171, His203, and Ser225 (45). A sequence logo was generated using WebLogo (46) to visualize residue conservation across the aligned sequences.

#### Phylogenetic relationship and amino acid composition analysis

A phylogenetic tree was constructed in MEGA X (47) using the Neighbor-Joining method with a Poisson correction model and 1,000 bootstrap replicates. Amino acid composition was also calculated in MEGA X to compare residue frequencies. The results aligned with the MSA and sequence logo findings, highlighting conserved features among the serine proteases.

### Structural modeling and interaction analysis

#### Tertiary structure prediction of serine protease and LEA peptides

The structure of serine protease from *B. toyonensis* was predicted by several web servers, such as Phyre2 (http://www.sbg.bio.ic.ac.uk/~phyre2/html/page.cgi?id=index) (48), SWISS-MODEL (https://swissmodel.expasy.org/) (49), Sequence Similarity Search (https://www.ebi.ac.uk/jdispatcher/sss/fasta) (50), and AlphaFold Colab (https://colab.research.google.com/github/sokrypton/ColabFold/blob/main/AlphaFold2.ipynb) (51). Simultaneously, the tertiary structure prediction for LEA I and K was conducted using the PEP-FOLD 3.5 web server (http://bioserv.rpbs.univ-paris-diderot.fr/PEP-FOLD) (52). The PDB files of the modeled structures were then submitted to the Structural Analysis and Verification Server (SAVESv6.1) (https://saves.mbi.ucla.edu/) for validation. Their quality was assessed through ERRAT (53), VERIFY3D (54), and PROCHECK (55). PROCHECK was utilized to determine the model’s stereochemical quality. ERRAT was additionally employed to analyze the overall model quality, whereas VERIFY3D determined the compatibility between the tertiary conformation and primary amino acid sequence of the predicted models (56). The predicted model was sent to GalaxyRefine (https://galaxy.seoklab.org/cgi-bin/submit.cgi?type=REFINE) (57) for refinement if the percentage of the Ramachandran plot generated was below 90%. The predicted models with the highest quality were chosen, and their structures were visualized using PyMOL (https://pymol.org/) (58).

#### Protein-protein docking of serine protease with LEA peptides

Protein-protein docking of serine protease from *B. toyonensis* with LEA-I and LEA-K was performed using the ClusPro web server (https://cluspro.org) (59), and their protein-protein interactions were analyzed using the PDBSum web server (https://www.ebi.ac.uk/thornton-srv/databases/pdbsum/).

#### Protein-ligand docking with azocasein substrate

Protein-ligand docking was conducted on AutoDock Vina Version 1.1.2 (https://vina.scripps.edu/download/) (60) by docking azocasein into the serine protease to determine the binding affinity. Additionally, the binding affinities between azocasein and Serine Protease + LEA-I, as well as Serine Protease + LEA-K, were measured through docking studies. The molecular structure of azocasein was modeled and optimized using Avogadro2 (https://avogadro.cc/) (61). Next, the PDB files of protease, Protease + LEA-I, Protease + LEA-K, and azocasein were changed and saved into a PDBQT file format for docking preparation. Molecular docking of azocasein with Serine Protease, Serine Protease + LEA-I, and Serine Protease + LEA-K was conducted within a grid box with a size of 61 × 51 × 70 Å. The *X*, *Y*, and *Z* coordinates for the center of the grid box were 2.81, −0.86, and 1.80, respectively. For each effective search, the default parameters were utilized, except that the number of modes was set to 1,000. The docked conformations having the lowest binding free energies were selected, and their interactions were visualized using Biovia Discovery Studio Version 2024 (https://discover.3ds.com/discovery-studio-visualizer-download) (62).

## RESULTS

### *In vitro* characterization of serine protease

#### Isolation and identification of protease-producing bacteria

**Fig 1:**
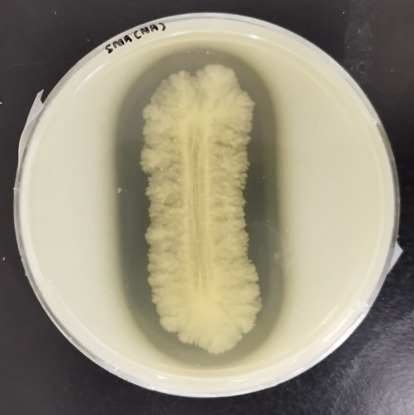
Casein hydrolysis test using skim milk agar.

After 3 months of fermenting kitchen waste, a brownish, acidic liquid with a pH of 3.9, known as garbage or eco enzyme, was obtained and stored in a plastic bottle with regular gas release. The liquid was diluted and cultured using the spread plate technique, thereby yielding four colonies, of which two distinct morphologies were observed and labeled as K1 and K2 (Fig. S4). K2 was demonstrated to have protease activity by clear zone formation on skim milk agar (Fig. 1). Following 16S rRNA sequencing, colony K2 was identified as *Bacillus toyonensis*.

#### Amplification, cloning, and transformation of protease gene

The protease gene from *B. toyonensis* was successfully amplified by PCR, which was confirmed by a single band on the agarose gel of approximately 800 bp (expected 792 bp), as shown in Fig. S6. Following that, the protease gene was successfully cloned into the pRSF-DUET vector using BamHI and HindIII, as confirmed by transformant growth on kanamycin-supplemented LB agar (Fig. S7). The presence of colonies on selective LB agar indicates successful transformation, thereby activating the antibiotic resistance conferred by the vector.

#### Expression and purification of recombinant protease

**Fig 2:**
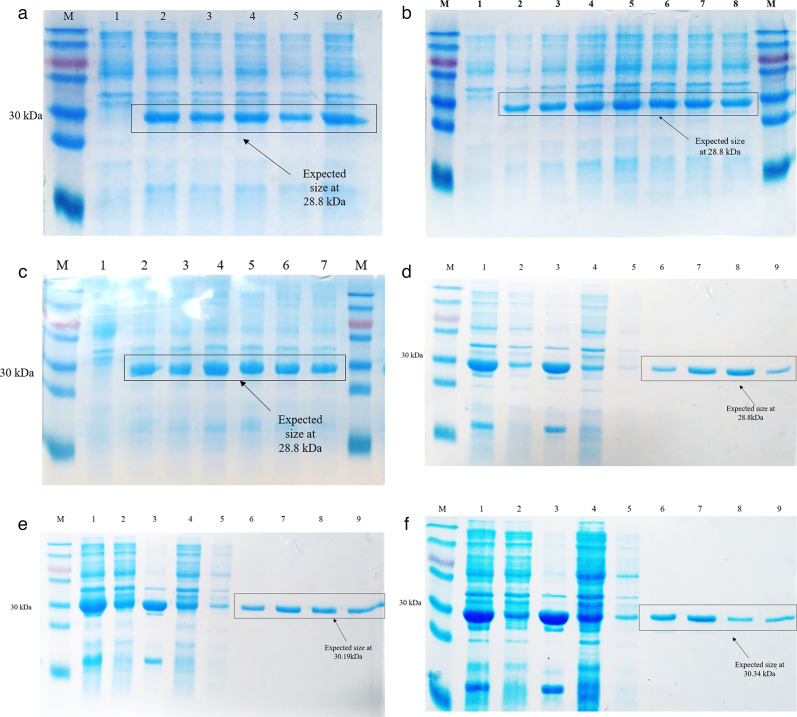
SDS-PAGE analysis of ProWL, ProLEA-I, and ProLEA-K. The optimal IPTG concentration (**a**) and incubation time (**b**) for recombinant protease expression were determined, and the molecular weight of the expressed proteases was confirmed (**c**). The optimal imidazole concentrations for elution and purification of ProWL (**d**), ProLEA-I (**e**), and ProLEA-K (**f**) were identified. (**a**) *M*: protein marker; Lane 1: *E. coli* BL21 (control); Lane 2: 0.1 mM IPTG; Lane 3: 0.2 mM IPTG; Lane 4: 0.4 mM IPTG; Lane 5: 0.6 mM IPTG; Lane 6: 1.0 mM IPTG. (**b**) *M*: protein marker; Lane 1: *E. coli* BL21 (control); Lanes 2 and 3: 12 h induction; Lanes 4 and 5: 18 h induction; Lanes 6 and 7: 20 h induction; Lane 8: 24 h induction; *M*: protein marker. (**c**) *M*: protein marker; Lane 1: *E. coli* BL21 (control); Lanes 2 and 3: ProWL; Lanes 4 and 5: ProLEA-I; Lanes 6 and 7: ProLEA-K. (d–f) *M*: protein marker; Lane 1: total protein; Lane 2: insoluble protein; Lane 3: soluble protein; Lane 4: flow-through; Lane 5: wash; Lanes 6–9: elution with imidazole at 20, 40, 60, and 80 mM, respectively.

The successful expression and purification of the recombinant protease were displayed using SDS-PAGE in Fig. 2. The optimized IPTG concentration to induce the expression of recombinant protease is 0.1 mM, along with an incubation time of 18 h. Following that, the optimal imidazole concentration for eluting ProWL is 60 mM, whereas 40 mM is most effective for eluting ProLEA-I and ProLEA-K. The molecular weight of the recombinant protease was identified as approximately 30 kDa (expected 28.80 kDa for ProWL, 30.18 kDa for ProLEA-I, and 30.32 kDa for ProLEA-K). In terms of protein content, the ProLEA-I was quantified at 0.33 mg, while ProWL and ProLEA-K were determined at 0.32 mg.

#### Effect of LEA peptides on protease yield and activity

The protease variants ProWL (without LEA), ProLEA-I (with LEA-I), and ProLEA-K (with LEA-K) were tested for proteolytic activity using azocasein as the substrate. As summarized in Table 1, ProLEA-K demonstrated the highest purification fold (1,638.00) and specific activity (16.38 U/mg), followed by ProLEA-I (312.00-fold, 9.36 U/mg) and ProWL (378.00-fold, 7.56 U/mg). These values were calculated based on increases in activity after IMAC purification from their respective crude extracts. Notably, ProLEA-K showed a drastic enhancement from 0.01 U/mg in the crude sample to 16.38 U/mg, indicating a significant gain in active enzyme yield. Despite similar protein yields after purification (0.32–0.33 mg), the markedly higher specific activity of ProLEA-K indicates that LEA-K primarily enhances catalytic efficiency rather than expression level.

**TABLE 1:** Purification table of recombinant proteases

Sample		Protein content, mg	Total activity, U	Specific activity, U/mg	Purification fold
ProWL	Crude protein	1.63	0.07	0.02	–^*a*^
	IMAC purified protein	0.32	2.42	7.56	378.00
ProLEA-I	Crude protein	1.97	0.06	0.03	–
	IMAC purified protein	0.33	3.09	9.36	312.00
ProLEA-K	Crude protein	1.69	0.03	0.01	–
	IMAC purified protein	0.32	5.24	16.38	1,638.00

^*a*^ –, not available.

#### Temperature study of protease co-expressed with LEA-like peptides

**Fig 3:**
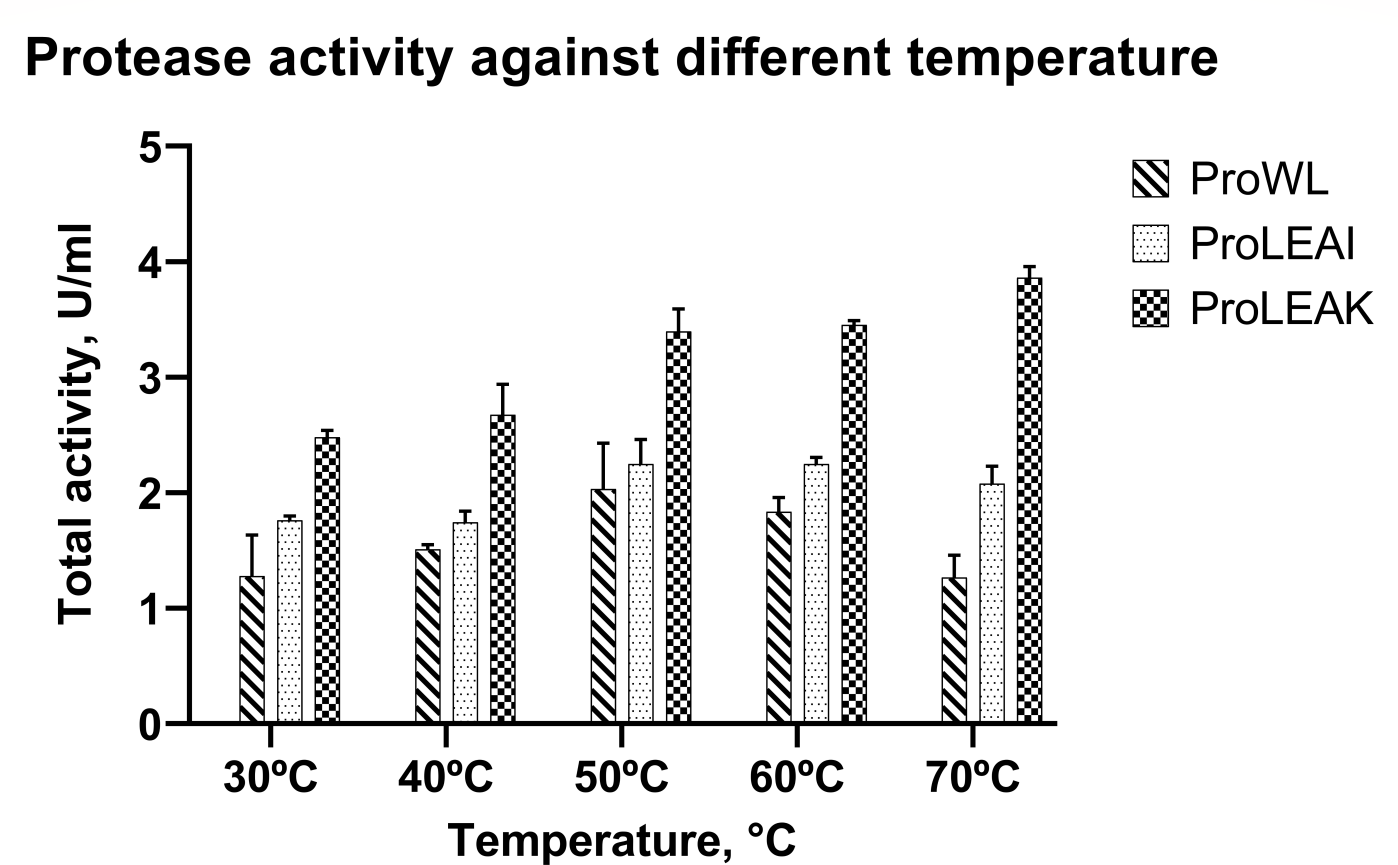
The protease activity against different temperatures.

The protease activity of the recombinant variants was assessed at 30°C, 40°C, 50°C, 60°C, and 70°C. As shown in Fig. 3, ProWL reached its maximum activity at 50°C but declined sharply at higher temperatures, indicating limited heat tolerance. In contrast, ProLEA-I and ProLEA-K maintained higher activity at elevated temperatures, with their highest activity recorded at 70°C.

Across the full temperature range, ProLEA-K showed 1.5–2.5-fold higher activity than ProWL and up to 1.3-fold higher activity than ProLEA-I. These results indicate that co-expression with LEA-K improves the ability of the protease to remain active under heat stress and supports better performance at high temperatures.

#### pH study of protease co-expressed with LEA-like peptides

**Fig 4:**
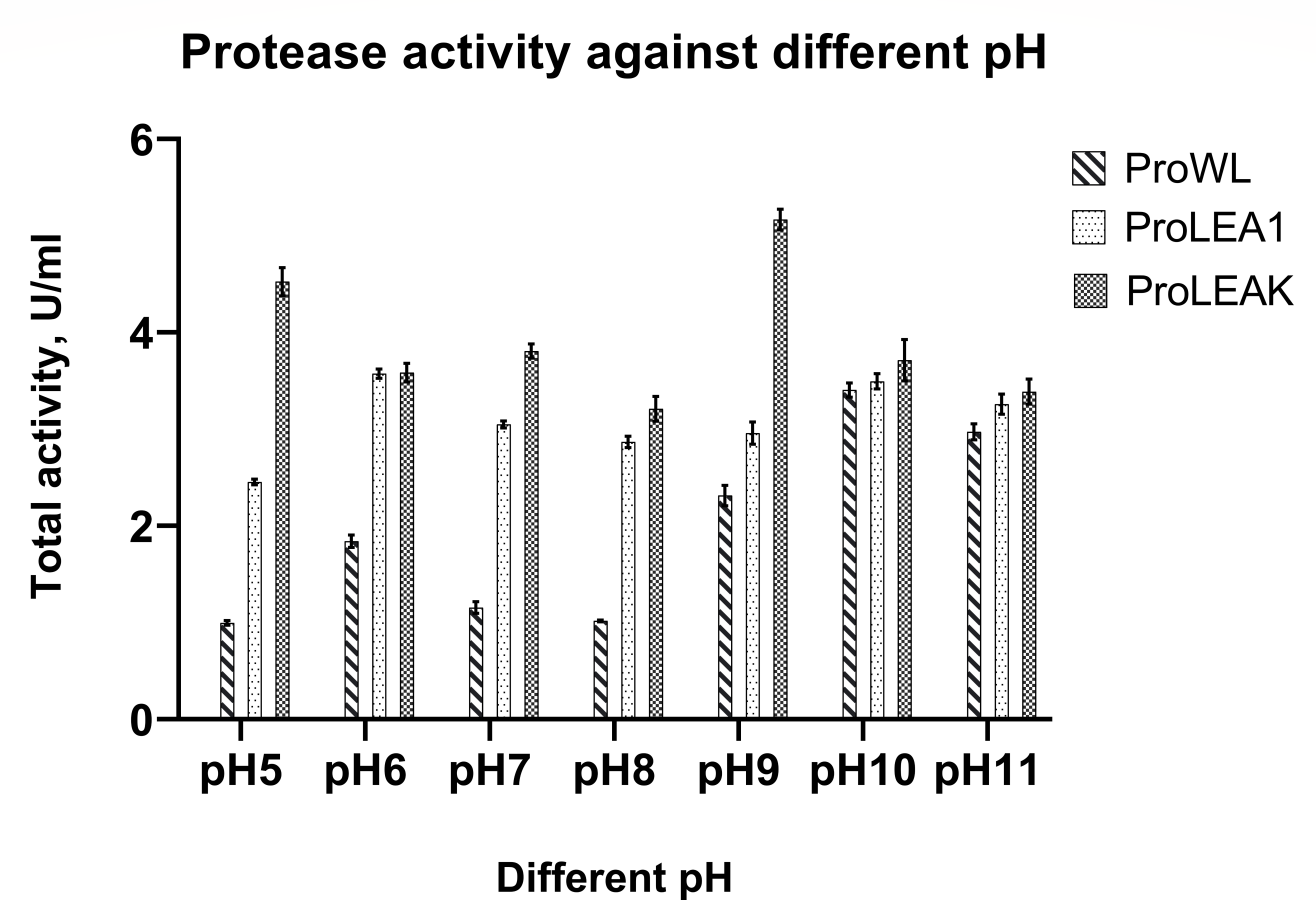
The protease activity against different pH levels.

The protease activity of the recombinant variants was evaluated at pH 5–11 (Fig. 4). ProWL displayed its highest activity at pH 10, with reduced performance under acidic conditions. In contrast, ProLEA-I reached maximum activity at pH 6, while ProLEA-K showed peak activity at pH 9 and maintained high activity from pH 5 to pH 11. These results indicate that the serine protease retains catalytic function in both acidic and alkaline environments, with co-expression of LEA-K providing the broadest pH tolerance. The variation in the optimum pH is suggested to be influenced by the isoelectric points (pI), which affect protein solubility and enzyme-substrate interactions.

#### Effect of detergent on recombinant protease activity

**Fig 5:**
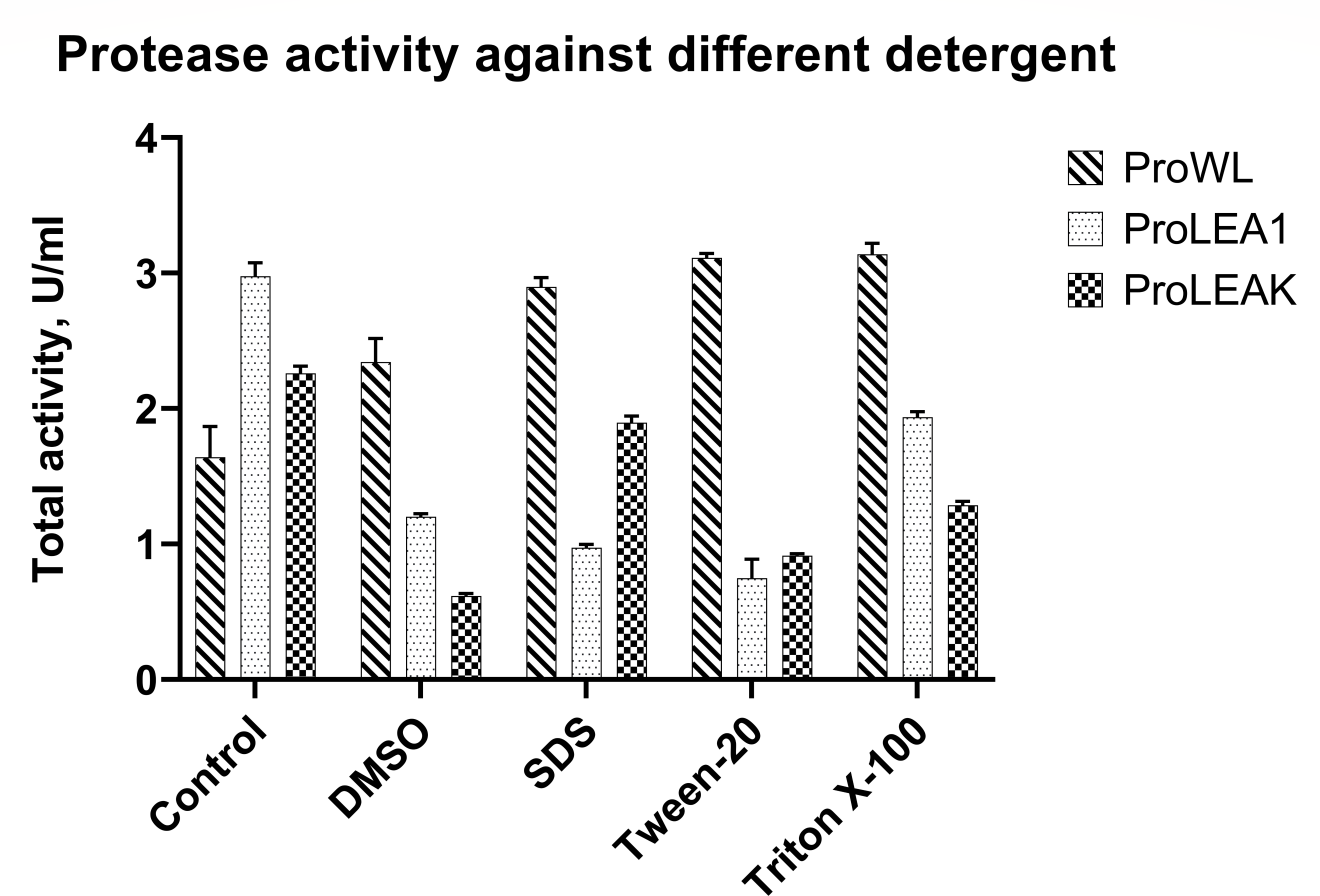
The protease activity in the presence of different detergents.

The recombinant protease activity was assessed in the presence of DMSO, SDS, Tween-20, and Triton X-100. Figure 5 demonstrates that the presence of detergent enhances the activity of ProWL but inhibits the activity of ProLEA-I and ProLEA-K, suggesting the variation in the optimum activity is likely due to the differences in the mechanism of detergents interacting with the protease active site.

### *In silico* analysis of serine protease sequences and structure

#### Sequence alignment and conserved residues

**Fig 6:**
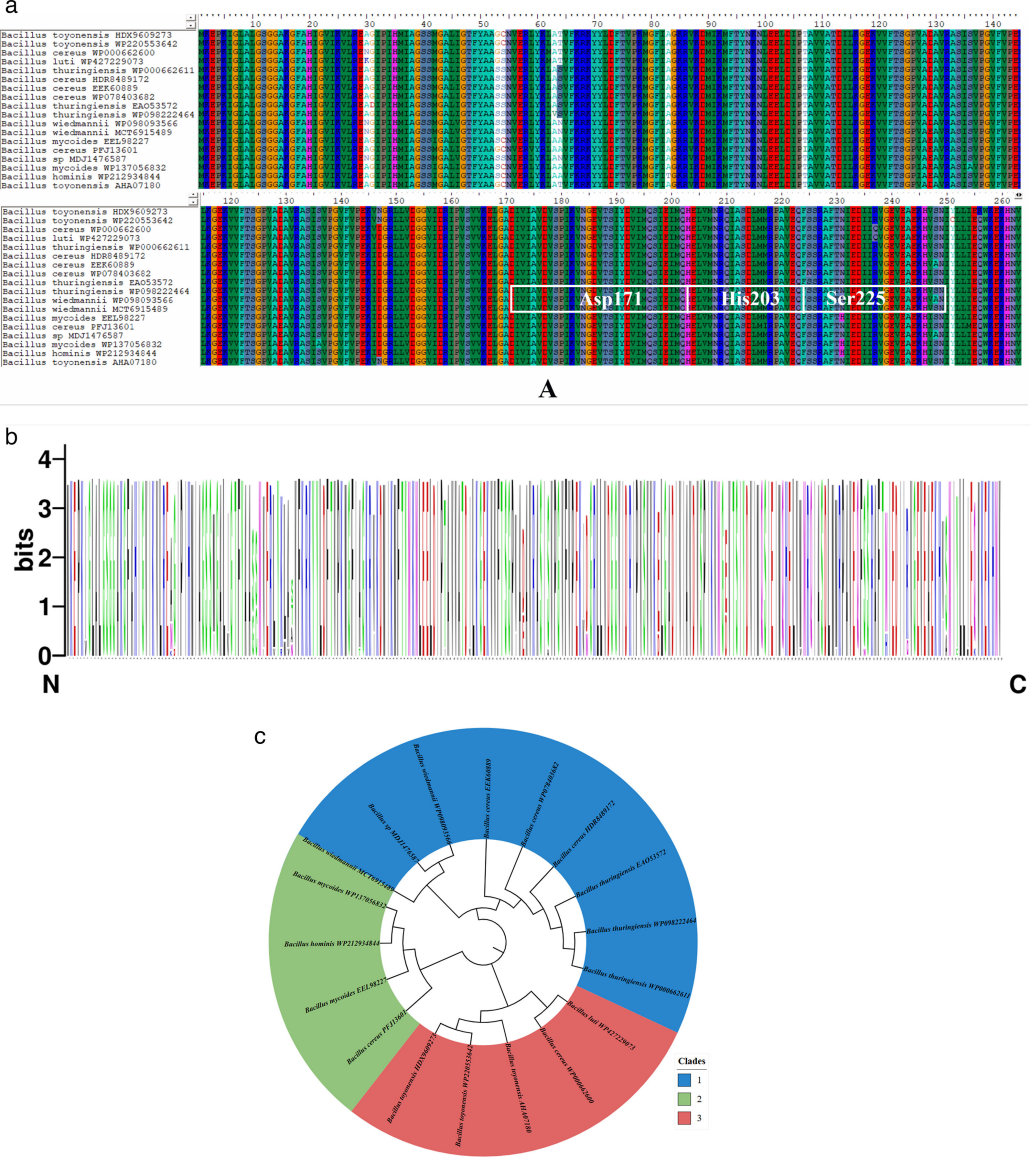
Comparative analysis of serine protease sequences from *Bacillus toyonensis* and related species, including (**a**) multiple sequence alignment highlighting conserved residues and catalytic motifs, (**b**) sequence logo illustrating residue conservation across aligned sequences, and (**c**) phylogenetic tree constructed using the Neighbor-Joining method showing three major clades.

The multiple sequence alignment of serine proteases from *Bacillus toyonensis* and related species (Fig. 6a) showed strong conservation across the sequences. The central region, which includes the catalytic triad residues Asp171, His203, and Ser225, was highly conserved. Motifs associated with enzymatic activity were maintained in all sequences, indicating evolutionary conservation of function.

#### Sequence logo analysis

A sequence logo generated from the alignment (Fig. 6b) confirmed residue-level conservation. Positions near the active site showed high information content, close to 4 bits, suggesting strong evolutionary constraint. Variability was mainly observed at the N-terminal and C-terminal regions, which are likely less critical to enzymatic function.

#### Phylogenetic analysis

The phylogenetic tree (Fig. 6c), constructed using the Neighbor-Joining method, grouped the protease sequences into three clades. *B. toyonensis* formed a tight cluster with *B. cereus* and *B. thuringiensis*, supporting a close evolutionary relationship. The clade structure reflects functional and taxonomic similarities among the species.

#### Amino acid composition

Amino acid frequency analysis (Table S2) showed that valine (12.21%), glycine (8.09%), alanine (7.63%), and threonine (5.83%) were the most abundant residues. These amino acids contribute to protein flexibility and core stability. The profiles were consistent across species and aligned with the conserved regions identified in the sequence alignment. Catalytic residues such as serine, aspartate, and histidine were uniformly present. Cysteine and tryptophan were found at low frequencies, consistent with the absence of disulfide bonds and limited aromatic residue content, a common feature of secreted bacterial enzymes.

#### Tertiary structure prediction of serine protease and LEA peptides

**Fig 7:**
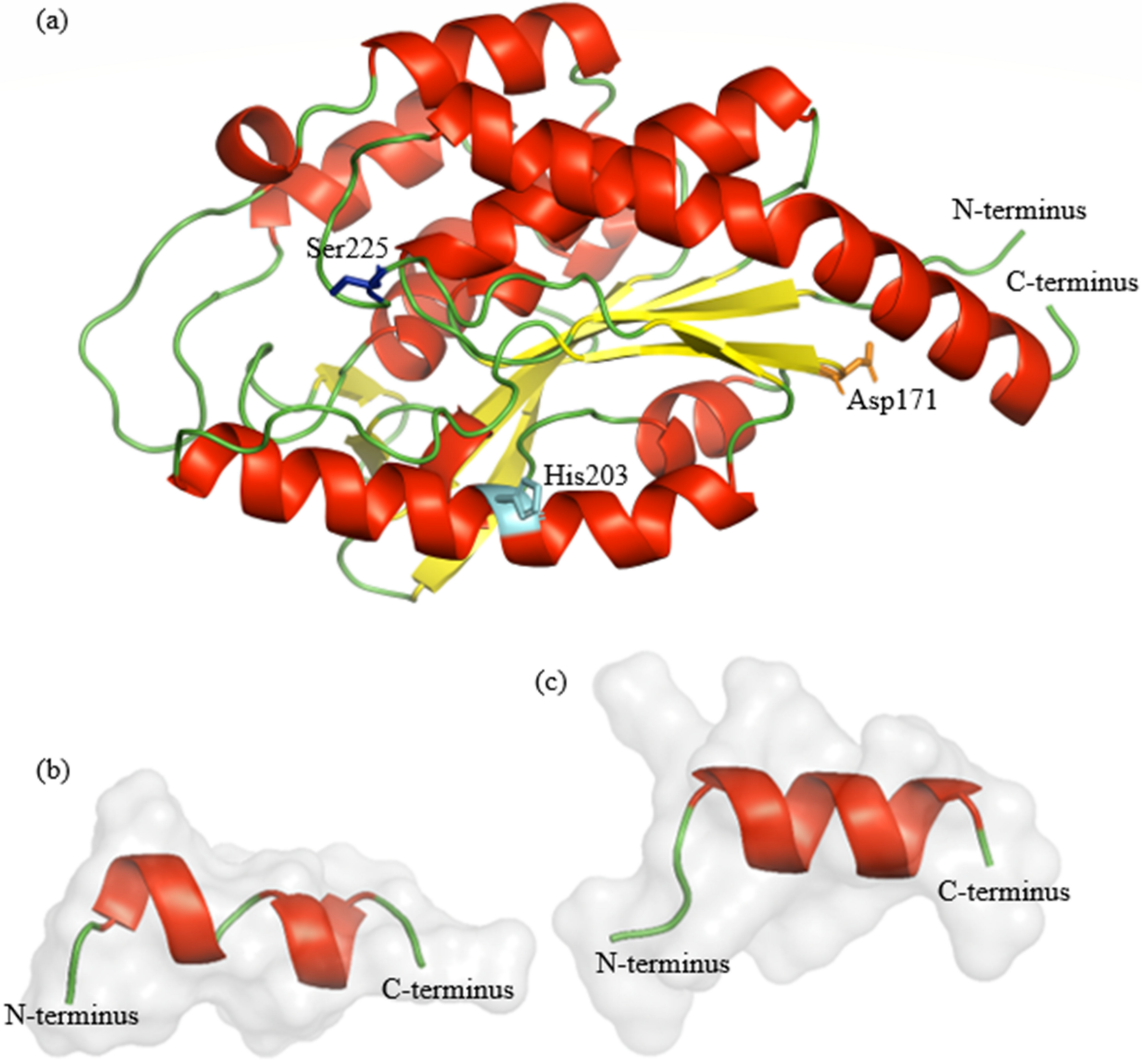
Tertiary structure of (**a**) serine protease predicted by Sequence Similarity Search, (**b**) LEA-I, and (**c**) LEA-K. The α-helices are red, the β-strands are yellow, and the loops are green in color. The catalytic triad of serine protease was shown in stick form. Asp171 is orange in color, His203 is cyan in color, and Ser225 is blue in color.

The tertiary structures of serine protease and LEA peptides were predicted and demonstrated in Fig. 7. Serine protease was made up of 11 α-helices and 8 β-strands connected by loops. A β-sheet consisted of five parallel strands located in the core of the protein. Serine protease was globular-shaped and adopted an α/β-hydrolase fold, and its catalytic triad was Asp171-His203-Ser225. The validation results (Fig. S14) of serine protease predicted by Sequence Similarity Search were 95.082% (ERRAT), 44.49% (VERIFY3D), and 94.3% (PROCHECK). The validation results of serine proteases predicted by several web servers are shown in Table S3 and Fig. S9 through S18. LEA-I peptide was made up of two α-helices, whereas LEA-K only had one α-helix in its structure. The α-helices were connected by loops.

#### Protein-protein docking

**Fig 8:**
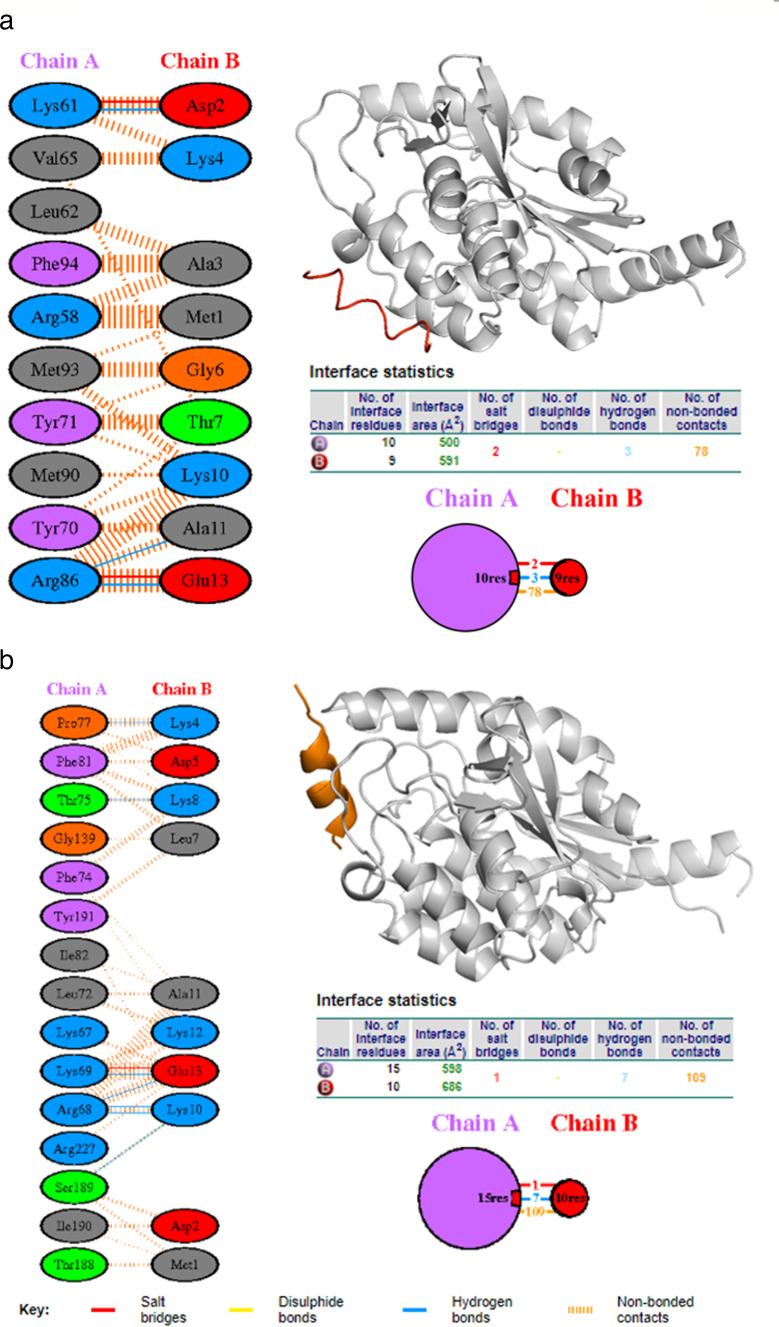
Structures of protein-protein docking best pose between (**a**) serine protease (Chain A) with LEA-I (Chain B) and (**b**) serine protease (Chain A) with LEA-K (Chain B). The left panel explains the residual interactions between serine protease and LEA peptides. The middle panel exhibits interface statistics.

After protein-protein docking, the interactions between the serine protease with LEA-I and LEA-K are shown in Fig. 8. The binding energy score calculated from the ClusPro server for Serine Protease-LEA-I was −366.8, where the LEA-I made 78 non-bonded interactions, 3 hydrogen bonds, and 2 salt bridges with the serine protease. A total of 10 residues from serine protease and 9 residues from LEA-I were involved in these non-bonded interactions. The binding energy score calculated for Serine Protease-LEA-K was −561.5, and the interactions that existed between the protein-peptide complex included 1 salt bridge, 7 hydrogen bonds, and 109 non-bonded interactions.

#### Molecular docking studies

**Fig 9:**
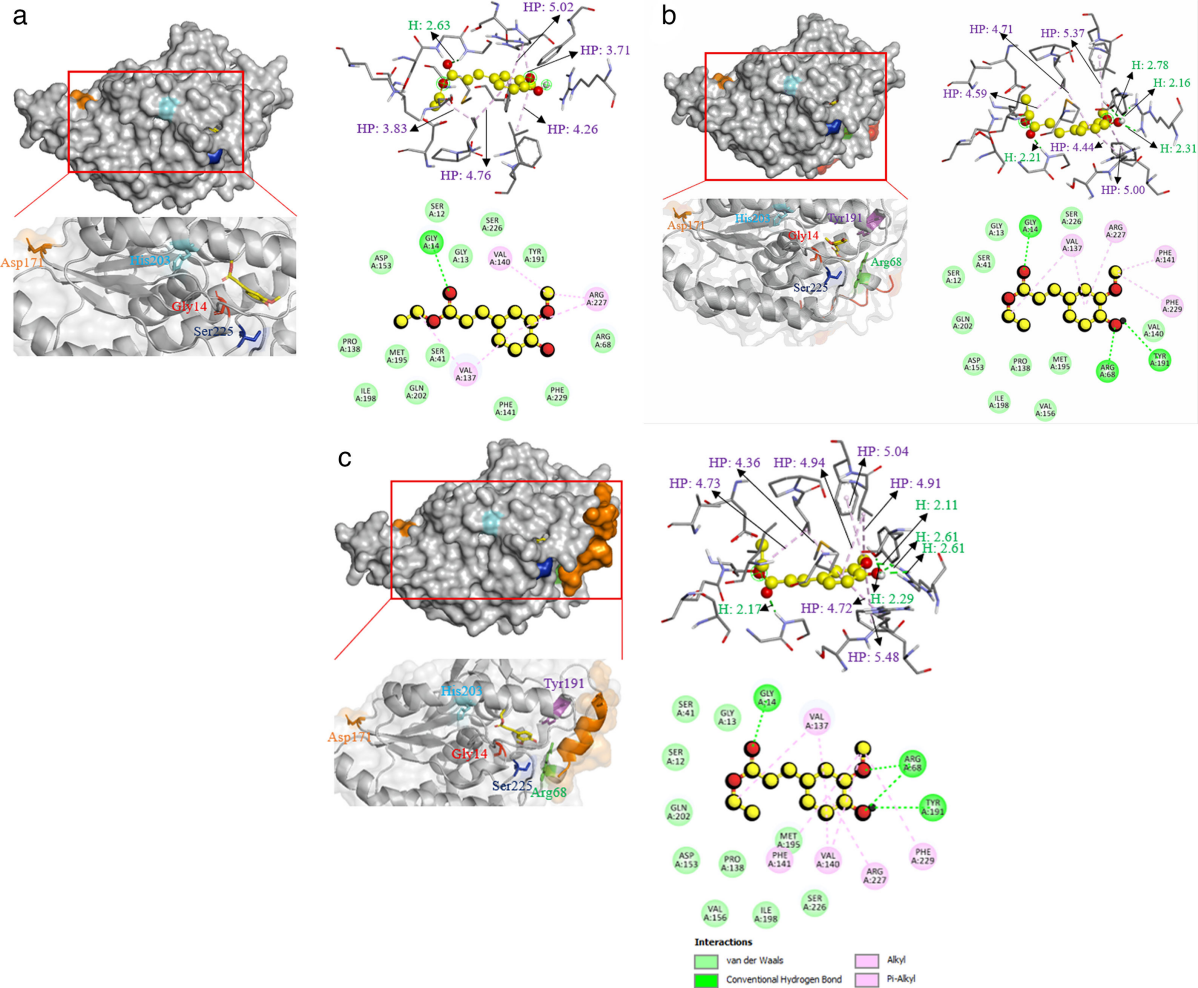
Molecular surface view of (**a**) serine protease_azocasein, (**b**) serine protease_LEA-I _azocasein, and (**c**) serine protease_LEA-K _azocasein. Left panel: the surface on serine protease is shown in gray color, while surfaces on LEA-I and LEA-K are shown in red and orange color. The azocasein is shown in yellow stick representation. Asp171 is orange in color, His203 is cyan in color, and Ser225 blue in color. Right panel: 3D and 2D interaction with hydrogen bond and other non-bonded interactions.

After molecular docking, the binding affinities between azocasein with serine protease, serine protease_LEA-I, and serine protease_LEA-K were determined, and their interactions were analyzed. The binding affinity of serine protease with azocasein was −5.8 kcal/mol. Azocasein formed a hydrogen bond with Gly14 and five hydrophobic interactions with Val137, Val140, and Arg227. In the docking between serine protease_LEA-I and azocasein, the interactions involved were four hydrogen bonds with Gly14, Arg68, and Tyr191, as well as five hydrophobic interactions with Val137, Phe141, Phe229, and Arg227. Similar interactions existed in the docking between serine protease_LEA-K with azocasein, except for an additional hydrogen bond with Arg68, as well as two additional hydrophobic interactions with Val140. The binding affinity of serine protease with azocasein in the presence of LEA-I and LEA-K was −6.7 kcal/mol and −6.9 kcal/mol, respectively. The molecular interactions between proteins and ligands are shown in Fig. 9 and Table 2. Figure 9 also demonstrated that azocasein docked at the catalytic site of serine protease with and without LEA peptides. Additionally, azocasein formed interactions, including hydrogen bonds, hydrophobic interactions, and van der Waals forces, with the conserved motif residues such as Gly14, Ser41, Val137, Asp153, and Arg68.

**TABLE 2:** Molecular docking interactions of serine protease (SP), serine protease_LEA-I (SP_LEA-I), and serine protease_LEA-K (SP_LEA-K) with azocasein

Protein	Ligand/substrate	Binding affinity (kcal/mol)	Interaction types	From	To	Distance (Å)
SP	Azocasein	−5.8	Hydrogen bond	Gly14	Azocasein	2.63
			Alkyl hydrophobic	Azocasein	Val137	3.83
			Alkyl hydrophobic	Azocasein	Val140	4.26
			Alkyl hydrophobic	Azocasein	Arg227	3.71
			Pi-alkyl hydrophobic	Azocasein	Val137	4.76
			Pi-alkyl hydrophobic	Azocasein	Arg227	5.02
SP_LEA-I	Azocasein	−6.7	Hydrogen bond	Gly14	Azocasein	2.21
			Hydrogen bond	Arg68	Azocasein	2.31
			Hydrogen bond	Arg68	Azocasein	2.16
			Hydrogen bond	Azocasein	Tyr191	2.78
			Alkyl hydrophobic	Azocasein	Val137	4.59
			Pi-alkyl hydrophobic	Phe141	Azocasein	5.37
			Pi-alkyl hydrophobic	Phe229	Azocasein	5.00
			Pi-alkyl hydrophobic	Azocasein	Val137	4.71
			Pi-alkyl hydrophobic	Azocasein	Arg227	4.44
SP_LEA-K	Azocasein	−6.9	Hydrogen bond	Gly14	Azocasein	2.17
			Hydrogen bond	Arg68	Azocasein	2.61
			Hydrogen bond	Arg68	Azocasein	2.11
			Hydrogen bond	Arg68	Azocasein	2.61
			Hydrogen bond	Azocasein	Tyr191	2.29
			Alkyl hydrophobic	Azocasein	Val137	4.73
			Alkyl hydrophobic	Azocasein	Val140	4.91
			Pi-alkyl hydrophobic	Phe141	Azocasein	5.04
			Pi-alkyl hydrophobic	Phe229	Azocasein	5.48
			Pi-alkyl hydrophobic	Azocasein	Val137	4.36
			Pi-alkyl hydrophobic	Azocasein	Val140	4.94
			Pi-alkyl hydrophobic	Azocasein	Arg227	4.72

## DISCUSSION

The inherent instability of enzymes under extreme industrial conditions, such as elevated temperatures, broad pH ranges, and exposure to surfactants or organic solvents, continues to limit their effectiveness in industrial processes (63). Among the strategies developed to address this, the use of LEA-derived peptides as molecular stabilizers has gained attention due to their ability to prevent aggregation, enhance folding, and improve stress tolerance in heterologous expression systems (28, 29). In this study, co-expression of a *Bacillus toyonensis* serine protease with short LEA-derived peptides (LEA-I and LEA-K) significantly improved enzyme yield, stability, and environmental tolerance.

### LEA-derived peptides enhance protease yield and reduce aggregation

Purification data (Table 1) revealed that ProLEA-K achieved a specific activity of 16.38 U/mg and a purification fold of 1,638.00, substantially higher than ProWL (7.56 U/mg, 378-fold) and ProLEA-I (9.36 U/mg, 312-fold). These improvements indicate that LEA peptides act during expression to minimize protein misfolding and aggregation, thereby increasing the fraction of correctly folded, active enzyme. LEA-like peptides are intrinsically disordered, highly hydrophilic proteins that form protective hydration shells around client proteins, reducing intermolecular hydrophobic contacts that lead to aggregation (28). Pathak et al. (29) further showed that LEA variants can be engineered to enhance these chaperone-like effects, and Almansoori et al. (37) demonstrated that LEA co-expression doubles *Bacillus licheniformis* lipase activity via similar anti-aggregation mechanisms. In this study, the substitution of glycine with lysine at positions 6 and 12 in LEA-K (Table 3) introduced additional positively charged side chains, increasing potential electrostatic contacts with acidic residues on the protease surface. This not only promotes solubility but also contributes to a denser protein-protein interaction network, consistent with the reduced aggregation observed in other LEA-assisted systems (33).

**TABLE 3:** LEA peptide variants and their corresponding amino acid sequences^*a*^

Peptides	1	2	3	4	5	6	7	8	9	10	11	12	13
LEA-I	M	D	A	K	D	**G**	T	K	E	K	A	**G**	E
LEA-K	M	D	A	K	D	**K**	T	K	E	K	A	**K**	E

^*a*^ The mutation sites within the LEA peptide sequences are indicated using bolded amino acid residues.

This enhancement was achieved without altering the host strain or expression system, unlike previous approaches where optimization strategies such as ethanol precipitation or genetic integration were used to improve protease activity (64). This suggests that LEA-K helps maintain protein folding during expression and purification, reducing misfolding or aggregation and thus increasing the yield of active enzyme.

### Thermal stability enhancement via LEA-protease interactions

ProLEA-K retained 2.5-fold higher activity at 60°C and remained catalytically competent up to 70°C, whereas ProWL activity declined sharply at the same temperature. Temperature profiling further confirmed a performance advantage for ProLEA-K across the 30°C–70°C range, with the clearest gains at 60°C–70°C. Docking analysis revealed that LEA-K formed ~7 hydrogen bonds and ~103 non-bonded contacts with the protease surface, compared to ~3 hydrogen bonds and ~78 contacts for LEA-I. These dense hydrogen-bond networks and van der Waals interactions, particularly around the catalytic triad (Asp171, His203, and Ser225), restrict loop mobility and reduce conformational flexibility, buffering against heat-induced unfolding. LEA-K’s α-helix interfaces with hydrophobic patches near the substrate-binding cleft, limiting solvent penetration and further protecting structural integrity under heat stress. Similar stabilization mechanisms have been observed in thermostable proteases from *Bacillus stearothermophilus* and *Bacillus licheniformis*, where increased hydrogen bonding and compact surface packing improved heat resistance (65, 66). The lysine substitutions at positions 6 and 12 in LEA-K enhance electrostatic interactions, a well-established factor in thermophilic enzyme stability (35, 67). Notably, analogous LEA-assisted stabilization was reported for *Bacillus licheniformis* lipase at 45°C using combined *in vitro* and *in silico* approaches (37).

### Broad pH tolerance and acid stability

ProLEA-K displayed exceptional pH tolerance, retaining high catalytic activity from pH 5 to pH 11, a rare property for acidic serine proteases, which typically exhibit narrow pH optima (63). Docking analysis revealed that LEA-K formed a denser interfacial network with the protease (~7 hydrogen bonds and ~103 non-bonded contacts) compared to LEA-I (~3 hydrogen bonds and ~78 contacts). These hydrogen-bond and van der Waals networks are less susceptible to disruption by protonation than salt bridges, thereby maintaining structural integrity under acidic conditions. This observation is consistent with the findings of Jamal et al. (65).

LEA-K’s lysine-rich surface (Lys4, Lys8, Lys10, and Lys12) engages in cation-π and CH–π interactions with aromatic residues (Phe74, Phe81, and Tyr191) and backbone carbonyls (Gly139), which remain stable at low pH. This finding aligns with the review by Dougherty (68). In our docking models, the LEA-K α-helix partially capped the substrate channel, limiting solvent penetration and shielding flexible loops around the catalytic triad from acid-induced distortion. These observations are in agreement with Metwally and Ikeno (35), who reported that LEA-K expression enhanced acid tolerance in *E. coli*, and they support the broader principle that positively charged, hydration-retaining peptides can stabilize proteins in low-pH environments. Additionally, the ProLEA-K–azocasein complex exhibited more evenly distributed contacts with conserved residues (Ser41, Val137, and Arg227), potentially preserving substrate orientation and catalysis despite protonation-state changes. Together, these features explain the broad pH stability of ProLEA-K, highlighting the protective role of LEA-K in maintaining activity across extreme conditions.

### Detergent tolerance

The stability of serine proteases in the presence of organic solvents, including detergents, is influenced by their ability to replace water molecules within the tertiary structure with solvent molecules without compromising catalytic function (69). In this study, ProWL displayed the highest activity across all tested detergents, maintaining near-optimal performance in the presence of SDS, Tween-20, and Triton X-100. In contrast, incorporation of LEA-like peptides (ProLEA-I and ProLEA-K) led to reduced activity under detergent conditions, likely due to steric interference of the active site by detergent molecules, which may hinder substrate binding to the catalytic triad.

Previous reports have highlighted the inherent detergent tolerance of wild-type serine proteases (69–71) as well as cases where LEA-like peptide recombination enhanced detergent stability (36). For example, Ng et al. (36) showed that cationic detergents such as Tween-20, Tween-80, and Triton X-100 improved the activity of Ab3 lipase, with Tween-20 yielding the highest enhancement. Similarly, Li et al. (72) reported that Tween-20 and Tween-80 surfactants could maximize the catalytic efficiency of serine proteases. However, in the present case, the protective structural effects conferred by LEA-K and LEA-I may be offset by detergent-induced displacement of stabilizing water molecules at the active site, leading to lower net activity compared to the wild type.

The integration of *in silico* and experimental analyses in this study provides a molecular explanation for these improvements. Multiple sequence alignment confirmed strong conservation of the catalytic triad (Asp171–His203–Ser225) and other functionally critical motifs, while amino acid composition analysis indicated features typical of secreted enzymes (high valine, glycine, alanine; low cysteine and tryptophan), supporting stability in extracellular environments (73). Docking analysis with azocasein showed that ProLEA-K had a more favorable binding free energy (−6.9 kcal/mol) compared to ProWL (−5.8 kcal/mol), with more evenly distributed contacts to conserved residues. These expanded substrate interactions likely maintain proper orientation and catalysis across varying temperatures, pH levels, and ionic conditions.

Collectively, the *in vitro* and *in silico* findings demonstrate that LEA peptides, particularly LEA-K, function as effective molecular stabilizers that enhance protease yield, thermostability, and pH tolerance, while modulating enzyme behavior under detergent-rich environments. This co-expression strategy therefore represents a practical and scalable approach for tailoring enzyme performance to specific industrial operating conditions rather than universally enhancing tolerance to all forms of stress.

### Limitations and future work

One of the major limitations in this study encompasses the limited variety of LEA-like peptides being investigated. Future research should expand the scope to include the full spectrum of LEA peptides, rather than focusing exclusively on LEA-I and LEA-K(29). In addition, although the findings of this study indicate that the LEA peptides can improve the structural stability of the serine protease, their interactions with other reagents remain complex and not fully understood. Hence, future work should focus on elucidating the precise molecular mechanisms underlying LEA-protease interactions, particularly their structural influence on the active site and confirmation of the enzyme under different conditions through structural analysis such as X-ray crystallography and NMR spectroscopy.

In addition, Wu and Ikeno (74) reported that the cyclized LEA peptide displays enhanced stability of the targeted protein compared to the linear one. This finding provided fundamental insights into the novel protein engineering approach through the incorporation of cyclized LEA peptides.

### Conclusion

Co-expression with LEA peptides, particularly LEA-K, markedly improved the yield, thermal tolerance, and functional robustness of the *Bacillus toyonensis* serine protease. ProLEA-K maintained high activity at 60°C and retained substantial activity up to 70°C, whereas the protease expressed alone (ProWL) exhibited a pronounced decline at elevated temperatures. ProLEA-K also remained active across a broad pH range spanning acidic to alkaline conditions, a property uncommon among acidic serine proteases. Evaluation under detergent-containing conditions revealed a differential response, with enhanced activity observed for ProWL but reduced activity for LEA-co-expressed variants, indicating a context-dependent effect of LEA peptides on enzyme performance. These biochemical findings were supported by *in silico* analyses, which demonstrated conservation of the catalytic triad, an amino acid composition characteristic of secreted enzymes, stable structural folding, and improved substrate binding affinity in LEA-containing variants. Collectively, these results establish LEA-K co-expression as an effective and adaptable strategy for engineering proteases with enhanced yield and stability under thermal and pH stress, while enabling tailored performance for specific industrial environments.

## Data Availability

All data are included in the manuscript, and all supporting information is provided in the submitted supplementary file.

## SUPPLEMENTAL MATERIAL

Supplemental material spectrum.03010-25-s0001.pdf Supplemental figures and tables.

ASM does not own the copyrights to Supplemental Material that may be linked to, or accessed through, an article. The authors have granted ASM a non-exclusive, world-wide license to publish the Supplemental Material files. Please contact the corresponding author directly for reuse.
