# Supplementary material for: Functional and structural characterization of a serine protease from Bacillus toyonensis co-expressed with short peptides from late embryogenesis abundant (LEA) proteins
Source: Microbiol Spectr. 2026 Jun 22;14(8):e03010-25. doi: 10.1128/spectrum.03010-25 (PMC13435873; doi:10.1128/spectrum.03010-25)
Supplement: Supplementary file 1 [file spectrum.03010-25-s0001.pdf]

Supplementary file

Functional and Structural Characterization of a Serine Protease from *Bacillus toyonensis* Co-expressed with Short Peptides from Late Embryogenesis Abundant (LEA) Proteins

Ammar Khazaal Kadhim Almansoori [1,2,3], Nurul Ain Syukriyah[1], Choo Yi Hang[1], Mustafa Abdulfattah[1,2], Shinya Ikeno[4], Lo Hui Yu, Haider Mahmod Jasim[5] and Rashidah Abdul Rahim[1,2*]

[1]School of Biological Sciences, Universiti Sains Malaysia, Gelugor, 11800 Penang, Malaysia.
[2]Centre for Chemical Biology (CCB), Universiti Sains Malaysia, SAINS@USM,29 Block B, 10, Persiaran Bukit Jambul, 11900 Bayan Lepas, Penang, Malaysia.
[3]Department of Medical Laboratory Techniques, Al-Mustaqbal University, 51001 Hillah, Babylon, Iraq.
[4]Department of Biological Functions and Engineering, Graduate School of Life Science and System Engineering, Kyushu Institute of Technology, 2-4 Hibikino, Wakamatsu Ward, Kitakyushu, 808-0196, Japan.
[5]College of pharmacy, Al-Muthanna University, Samawah, 66001, Iraq.

***Corresponding author:**
Rashidah Abdul Rahim
Email: rshidah@usm.my
Phone: +60-4-6536158
Fax: +60-4-6565125

A

PROTEIN SEQUENCE

>AHA07180.1 Serine protease [*Bacillus toyonensis* BCT-7112]

MKEPKIGLALGSGGAKGFAHIGVIKVLREAGIPIHMIAGSSMGALIGTFYAAG
CNVERLYKLATVFKRKYYLDFTVPKMGFIAGKRVKDMIKMFTYNKNLEELD
IPTAVVATDILKGEKVVFTSGPVADAVRASISVPGVFVPEKVNGRLLVDGGVID
RIPVSVVKELGADIVIAVDVSPIKVNGEVTSIYDVIMQSIEIMQHELVMNRQIA
SDLMMRPAVEQFSSRAFTNIEDIIRVGEVEAEKHVSNIYLLIEQWKEKHNV

B

NUCLEOTIDE SEQUENCE

ATGAAAGAACCGAAGATTGGTTTAGCGCTTGGATCTGGGGGAGCGAAAG
GATTTGCTCATATTGGCGTTATAAAAGTTTTAAGGGAAGCGGGTATCCCGA
TTCATATGATTGCAGGGAGTAGTATGGGGGCCTTAATAGGTACATTTTATGC
AGCTGGCTGTAACGTTGAACGATTGTATAAATTGGCGACTGTTTTTAAGAG
GAAATACTATTTAGATTTCACGGTACCAAAAATGGGGTTTATTGCTGGTAA
ACGTGTCAAAGATATGATTAAAATGTTTACATATAATAAAAATTTAGAAGA
GTTAGATATCCCGACAGCTGTTGTGGCTACTGACATATTGAAGGGGGAGA
AAGTGGTTTTTACAAGTGGTCCTGTTGCAGATGCGGTGAGGGCTAGTATAT
CTGTCCCTGGAGTGTTTGTGCCAGAAAAGGTAAATGGACGATTATTAGTAG
ATGGTGGAGTGATTGATCGCATCCCAGTTTCGGTTGTAAAAGAGTTAGGTG
CCGATATTGTTATTGCTGTTGATGTATCTCCGATTAAGGTGAATGGTGAGGT
TACATCTATTTATGATGTAATTATGCAAAGTATTGAAATTATGCAGCATGAG
CTTGTAATGAATCGGCAAATTGCATCAGATTTAATGATGCGACCAGCTGTG
GAACAATTTAGTTCACGTGCTTTTACAAACATTGAAGACATTATTCGAGTC
GGAGAAGTAGAAGCAGAAAAACATGTTTCAAATATTTATTTATTAATTGAG
CAGTGGAAGGAGAAACATAATGTTTAA

Fig. S1. Protein sequence (A) and nucleotide sequence (B) of serine protease from *Bacillus toyonensis*.

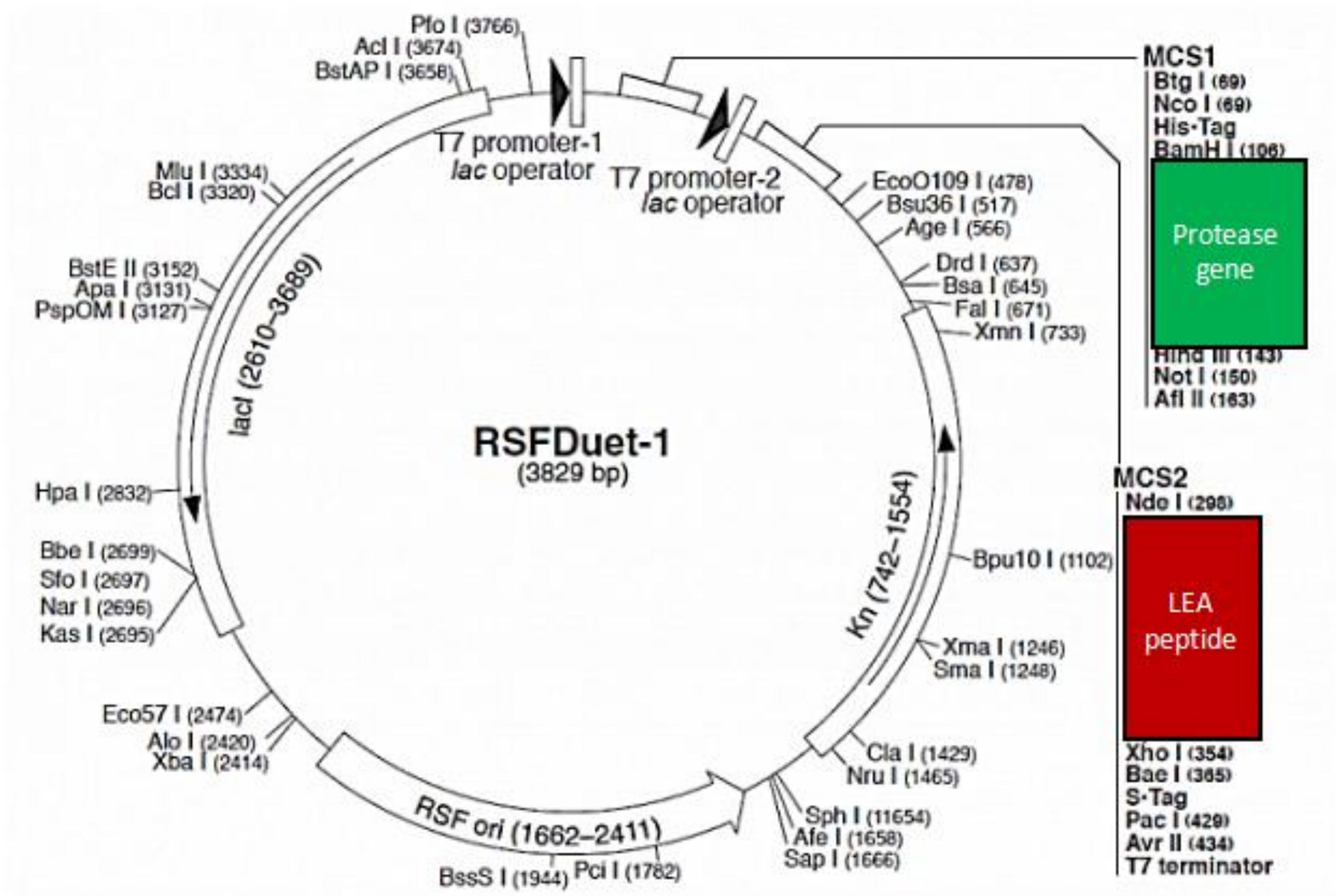

Fig. S2. The pRSF-Duet vector map with two multiple cloning sites.

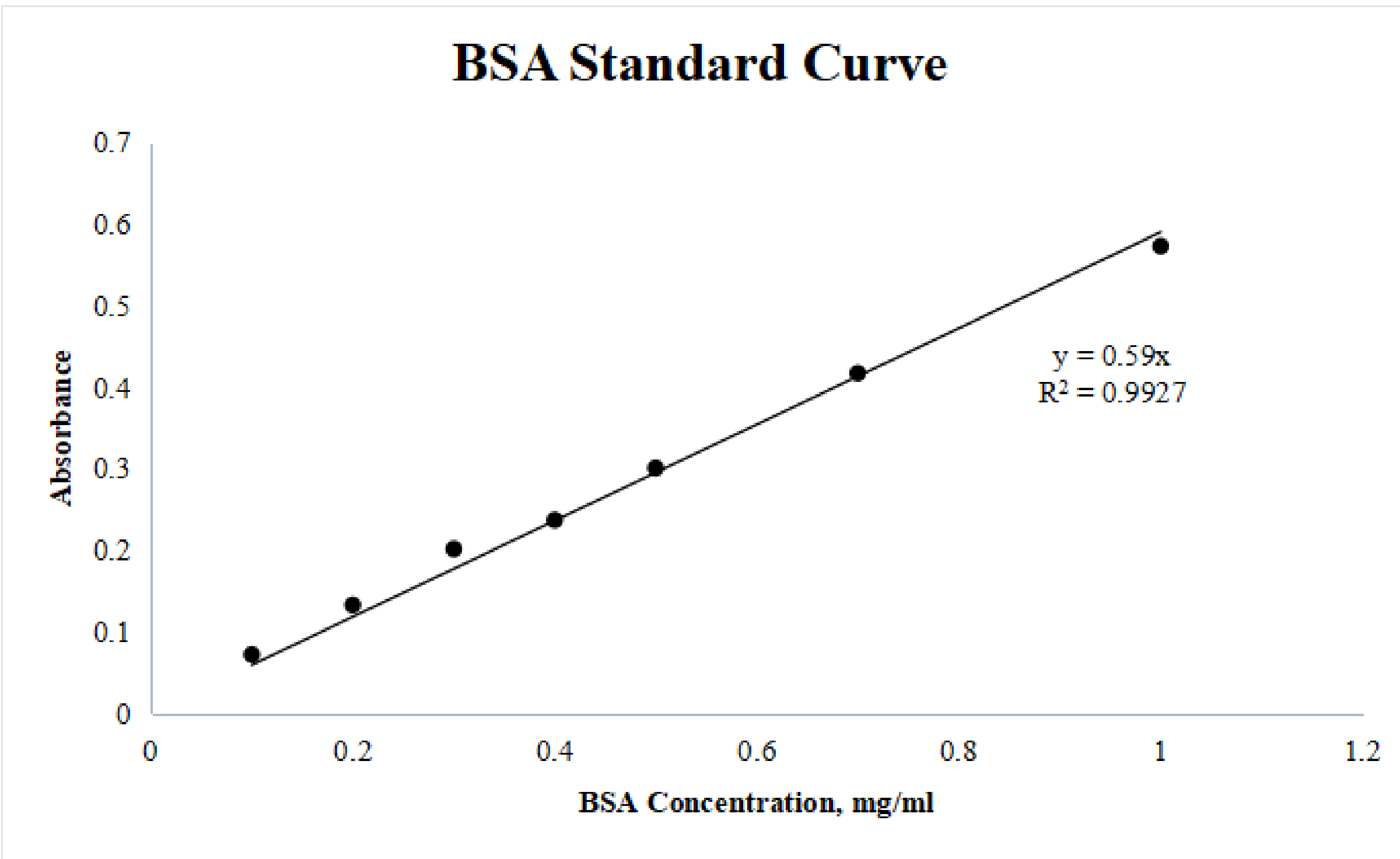

Fig. S3. The BSA standard curve.

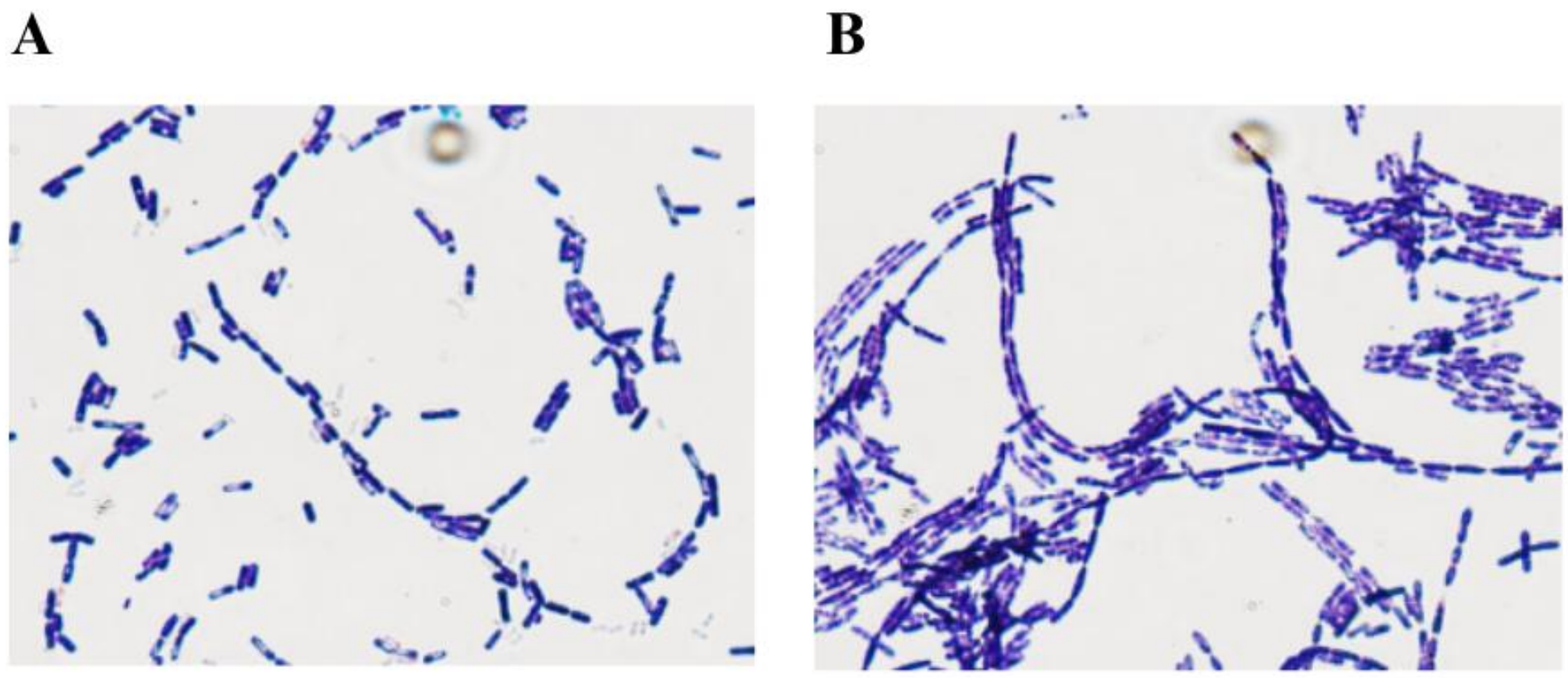

Fig. S4. Morphology observation of the isolated colonies. (A) refers to the first colony isolated from the kitchen waste fermentation liquid, while (B) refers to the second colony isolated from the kitchen waste fermentation liquid.

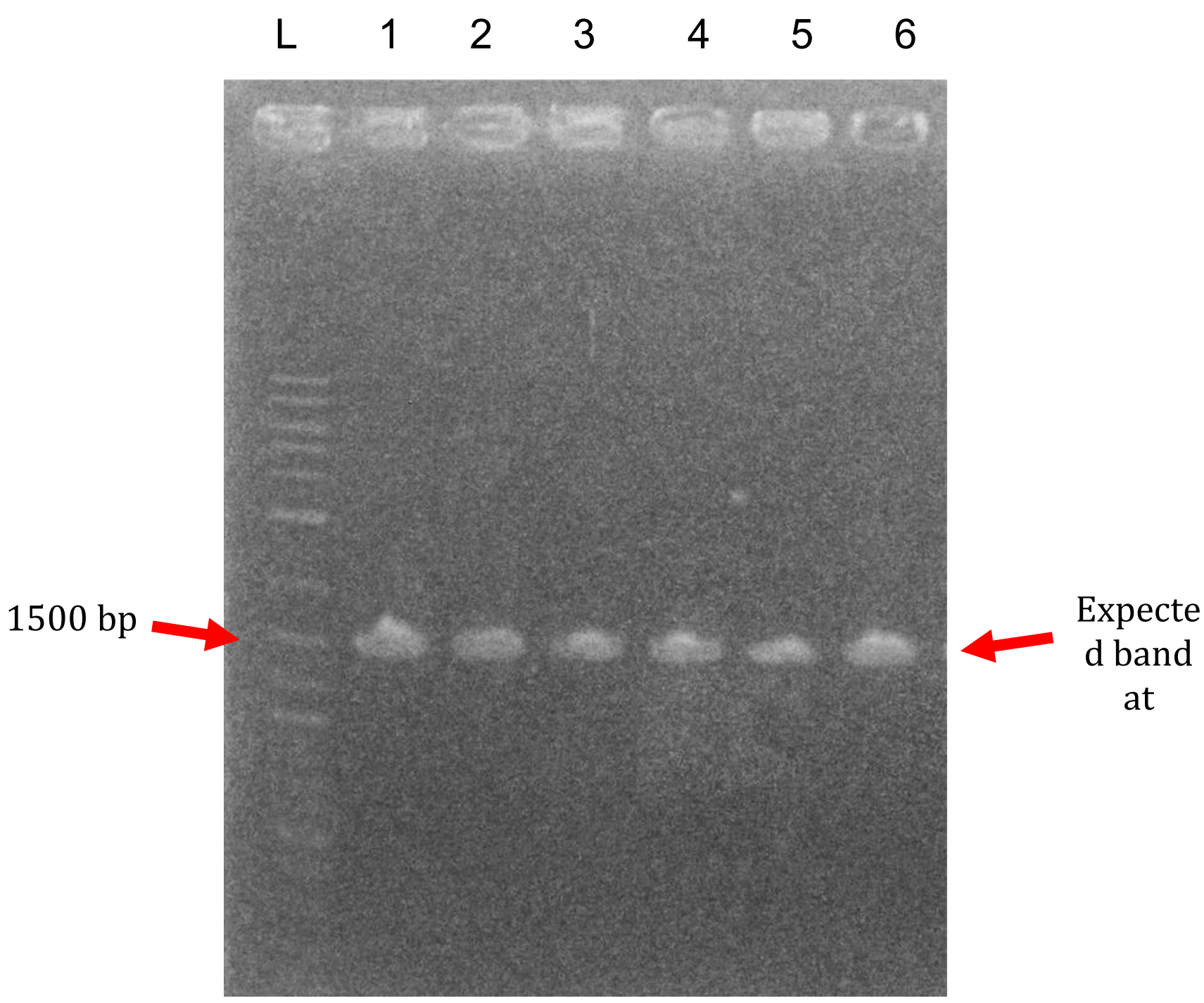

Fig. S5 Amplification of 16S rRNA gene through PCR. L: 1KB DNA ladder, 1-3: Replicated samples of K1 and 4-6: Replicated samples of K2.

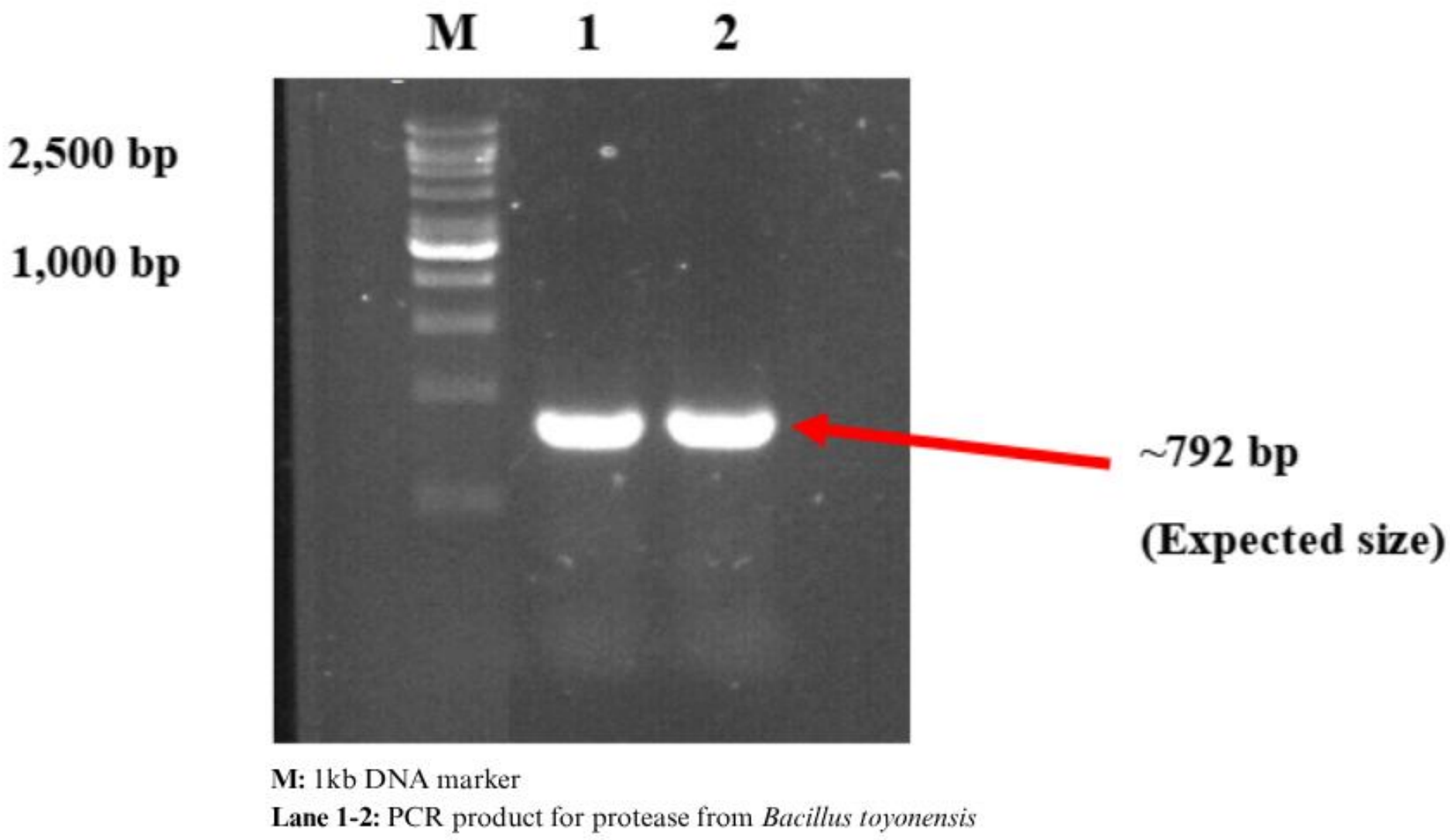

Fig. S6. Amplification of the protease gene through PCR.

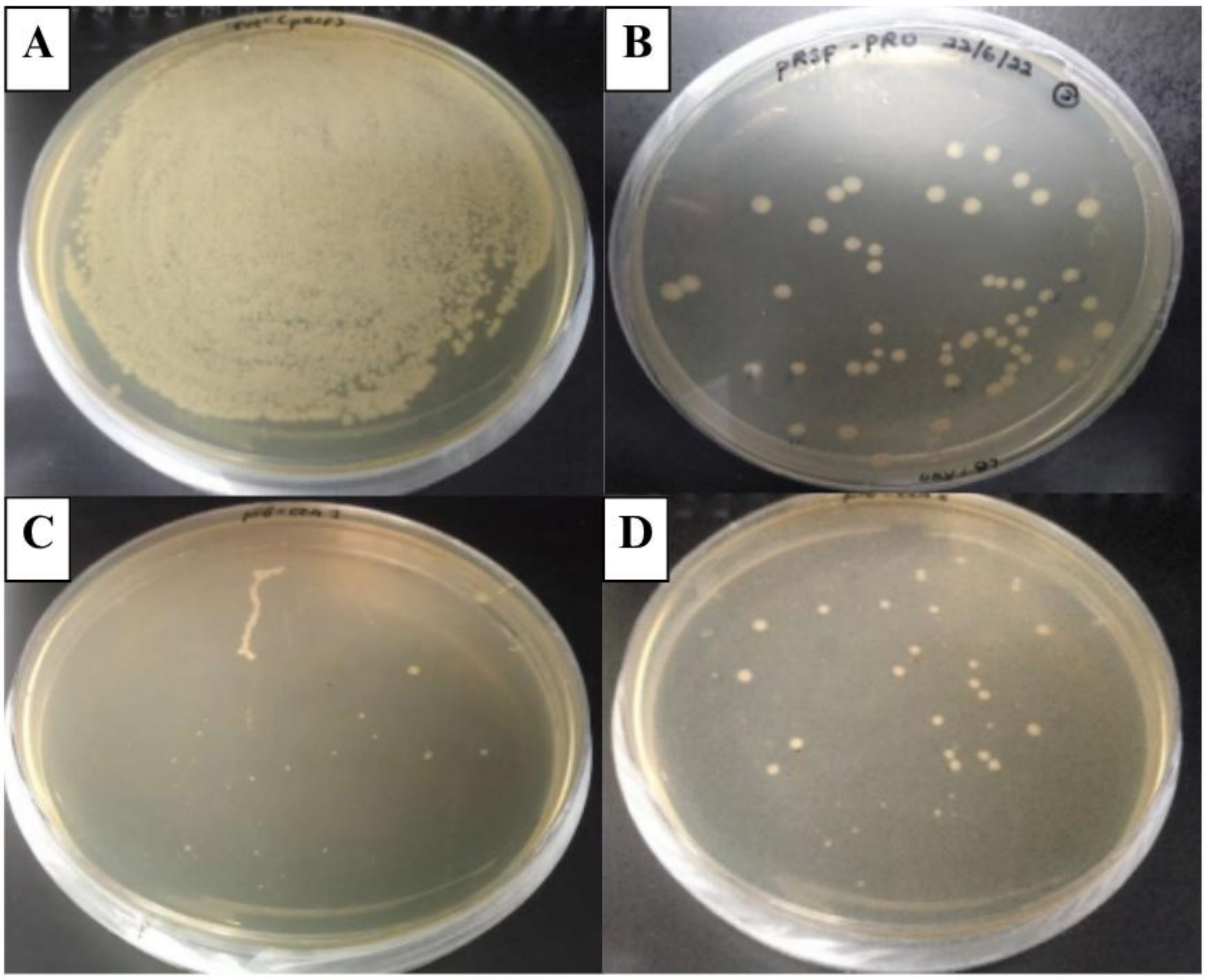

Fig. S7. Transformation plate after overnight incubation at 37ºC. (A) Positive control plate (pRSF-DUET vector only), (B) Recombinant protease without LEA peptides, (C) Recombinant protease with LEA 1 peptides, (D) Recombinant protease with LEA K peptides.

Phyre2:

(a) ERRAT:

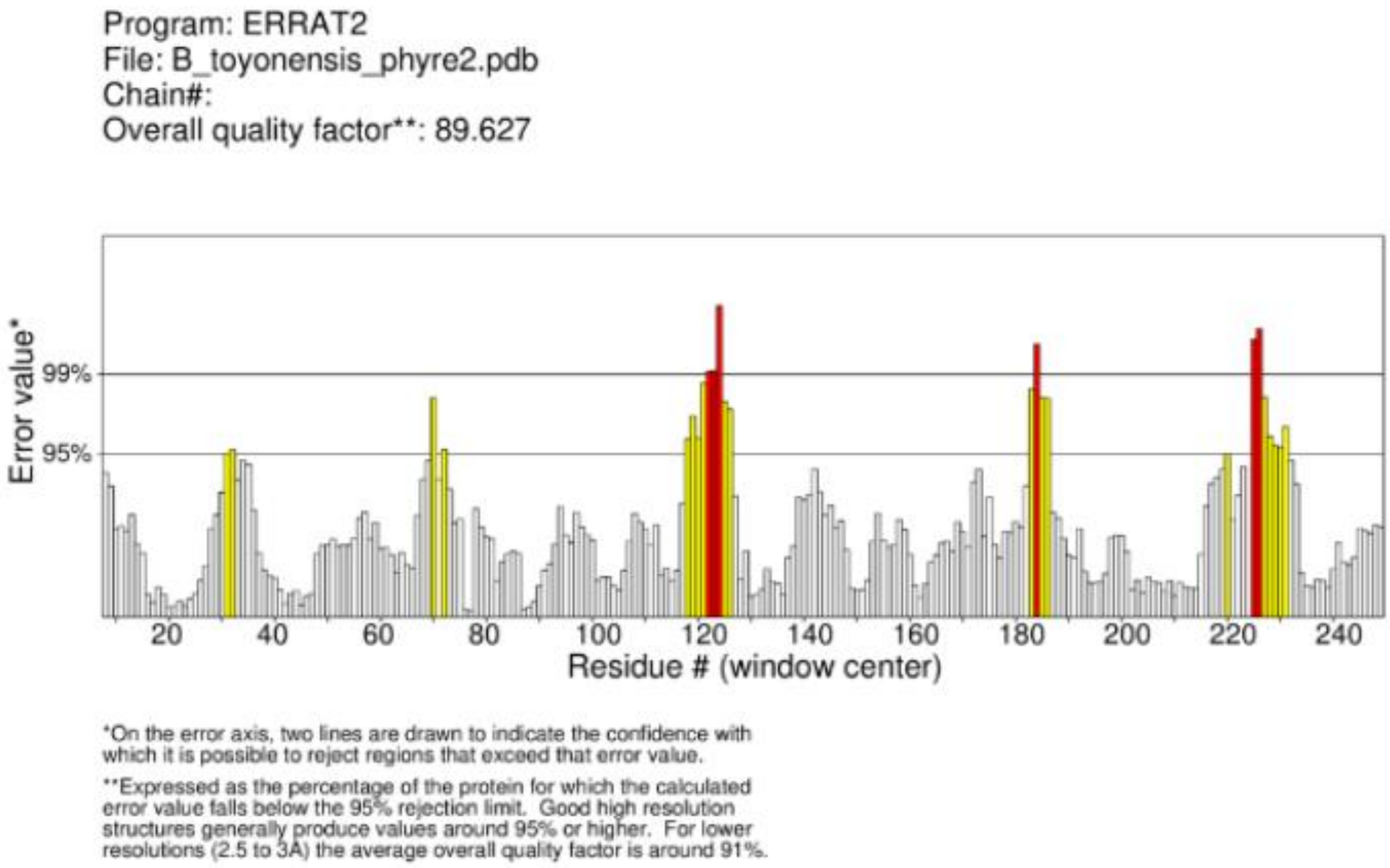

(b) VERIFY3D:

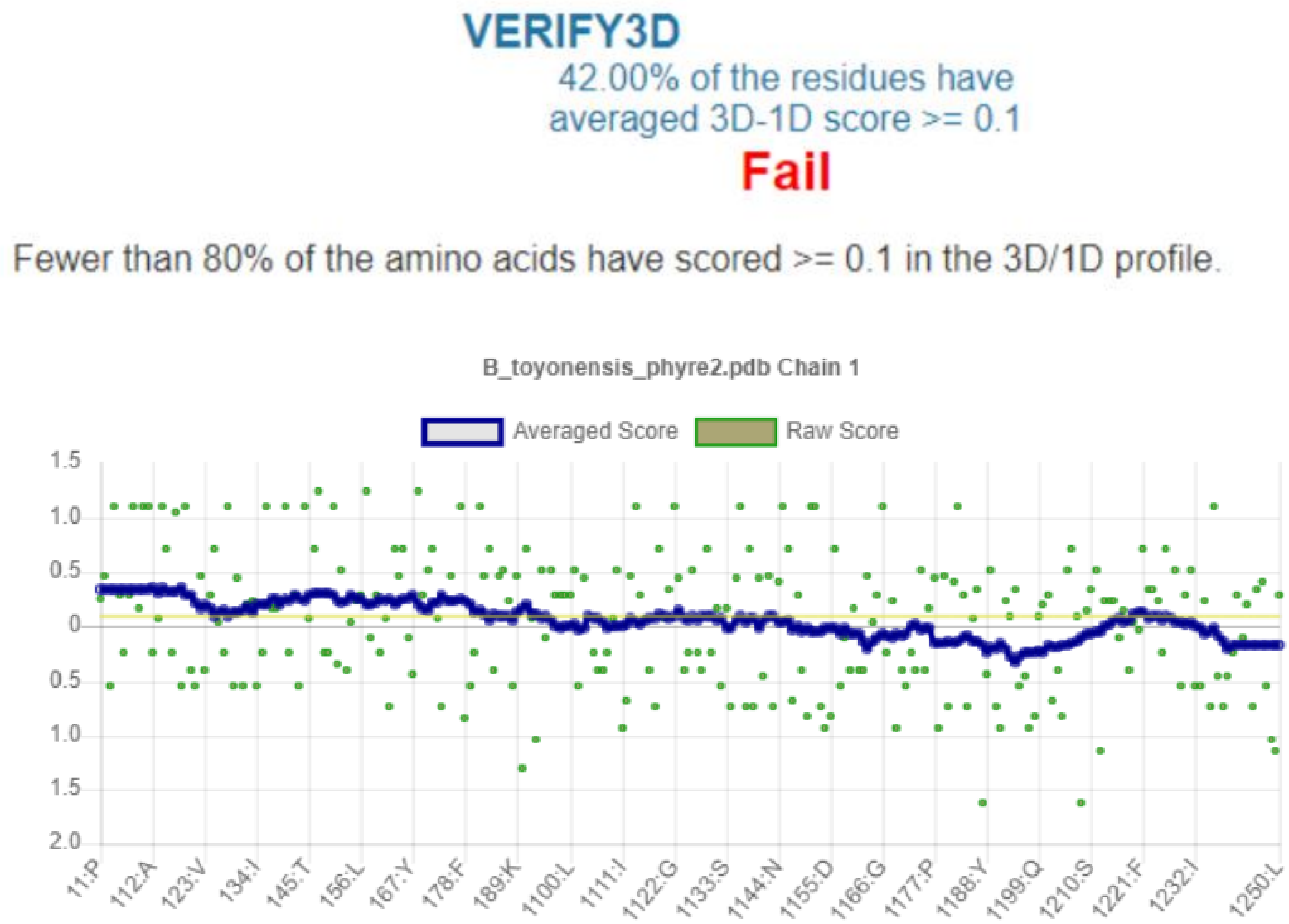

(c) PROCHECK:

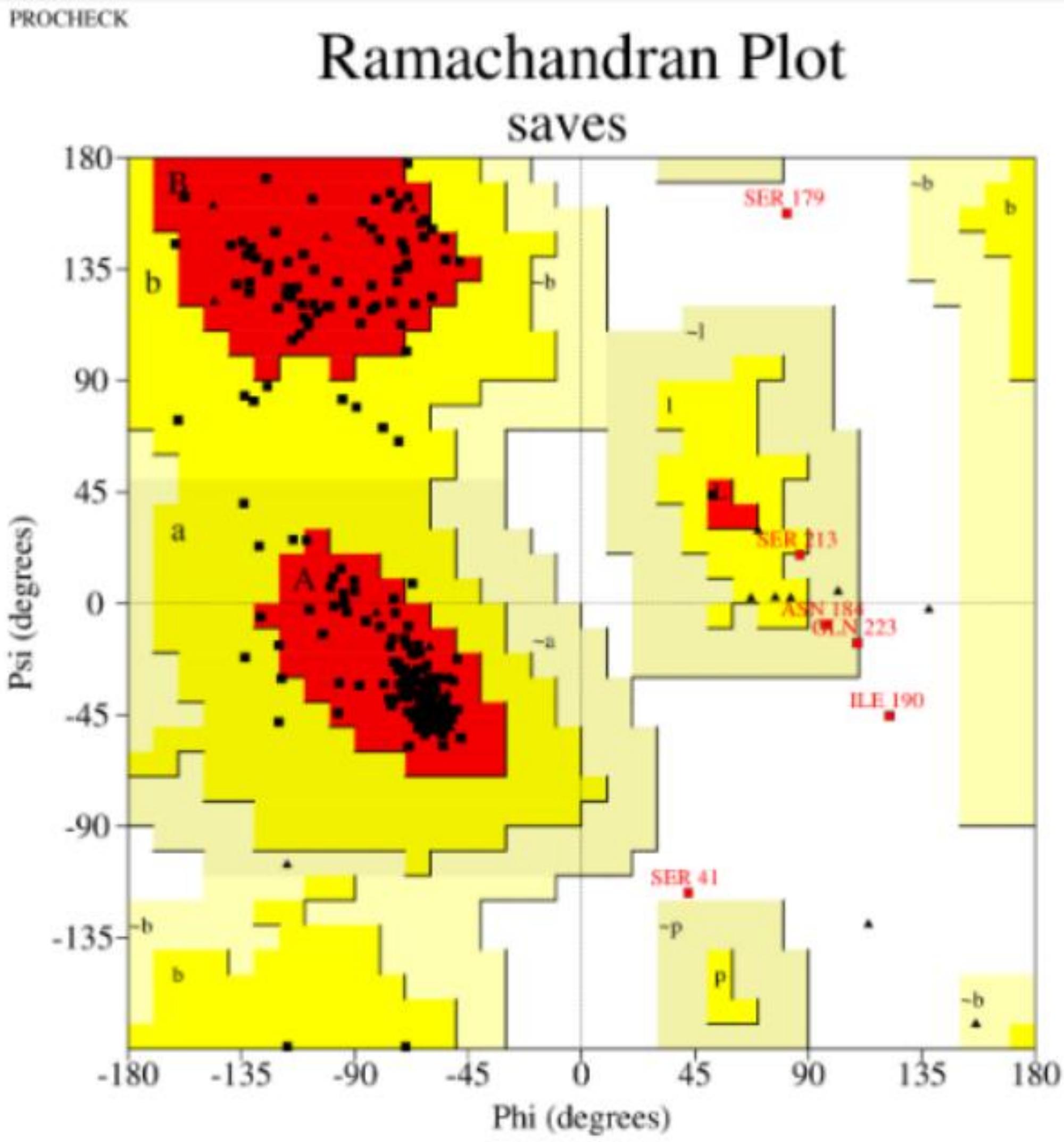

Plot statistics

Residues in most favoured regions [A,B,L]	192	88.5%
Residues in additional allowed regions [a,b,l,p]	19	8.8%
Residues in generously allowed regions [~a,~b,~l,~p]	3	1.4%
Residues in disallowed regions	3	1.4%
Number of non-glycine and non-proline residues	217	100.0%
Number of end-residues (excl. Gly and Pro)	1	
Number of glycine residues (shown as triangles)	22	
Number of proline residues	10	
Total number of residues	250	

Based on an analysis of 118 structures of resolution of at least 2.0 Angstroms and R-factor no greater than 20%, a good quality model would be expected to have over 90% in the most favoured regions.

Fig. S8. Validation result of serine protease predicted by Phyre[2]. (a) ERRAT, (b)VERIFY3D, and (c) PROCHECK.

AlphaColab:

(a) ERRAT:

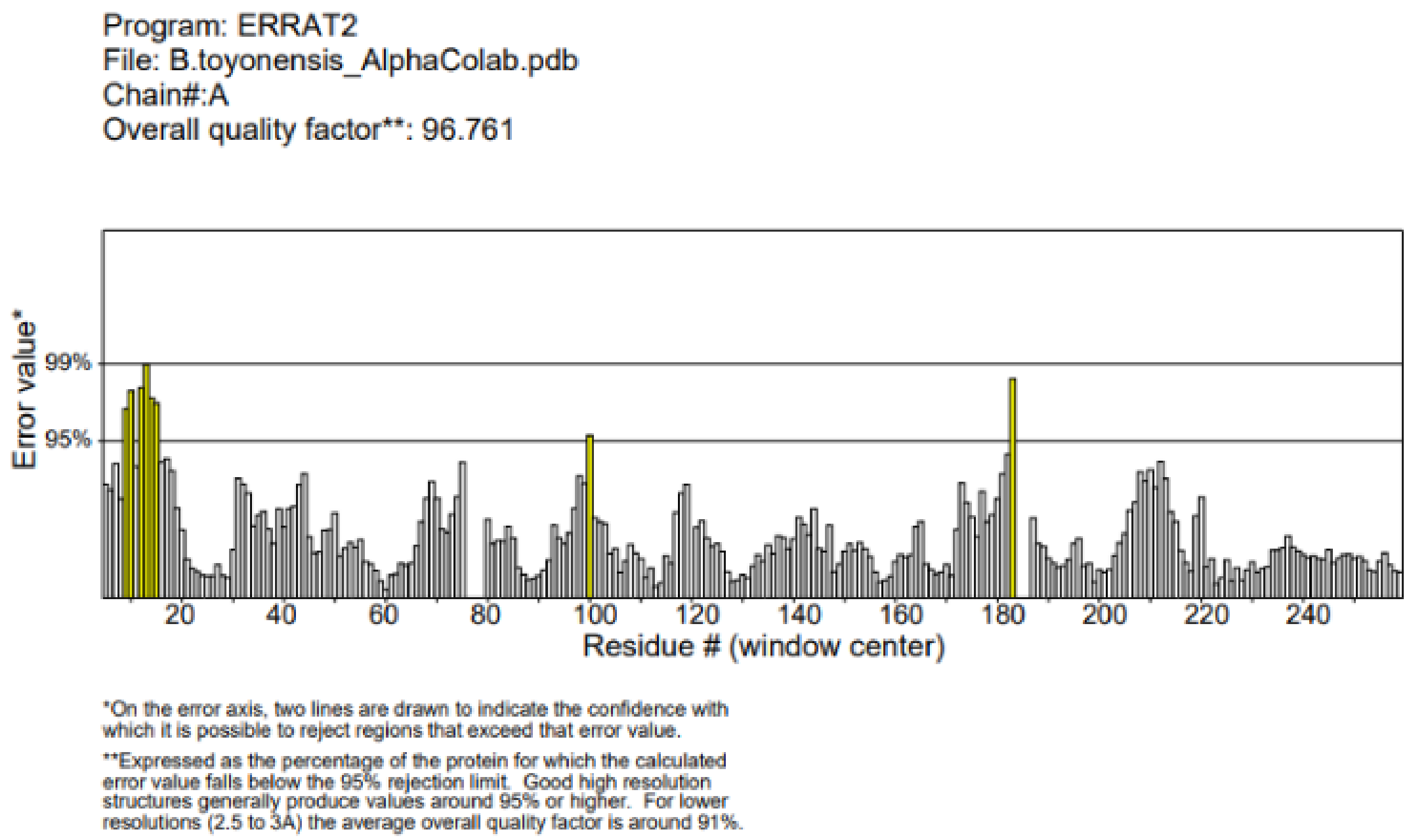

(b) VERIFY3D:

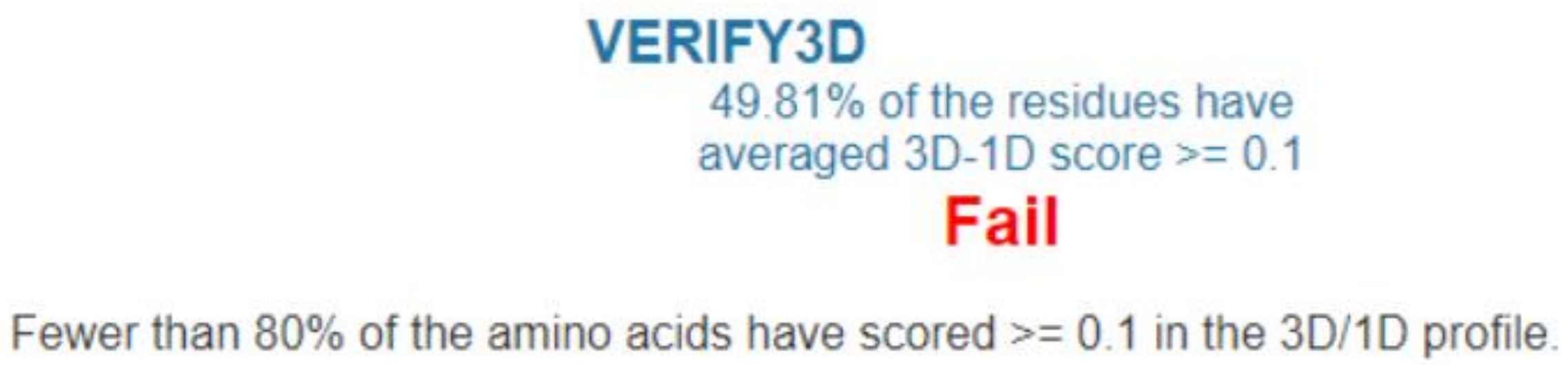

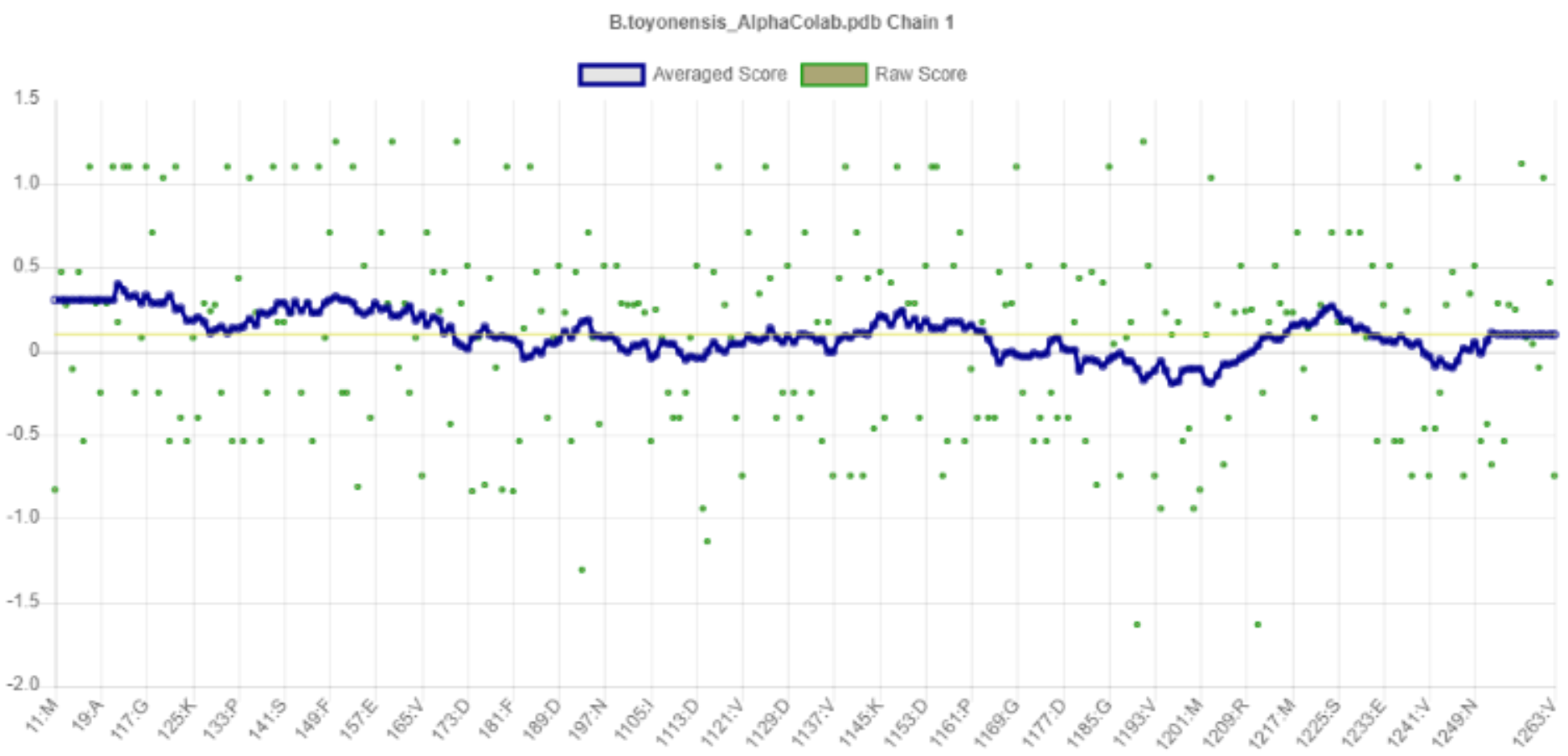

(c) PROCHECK:

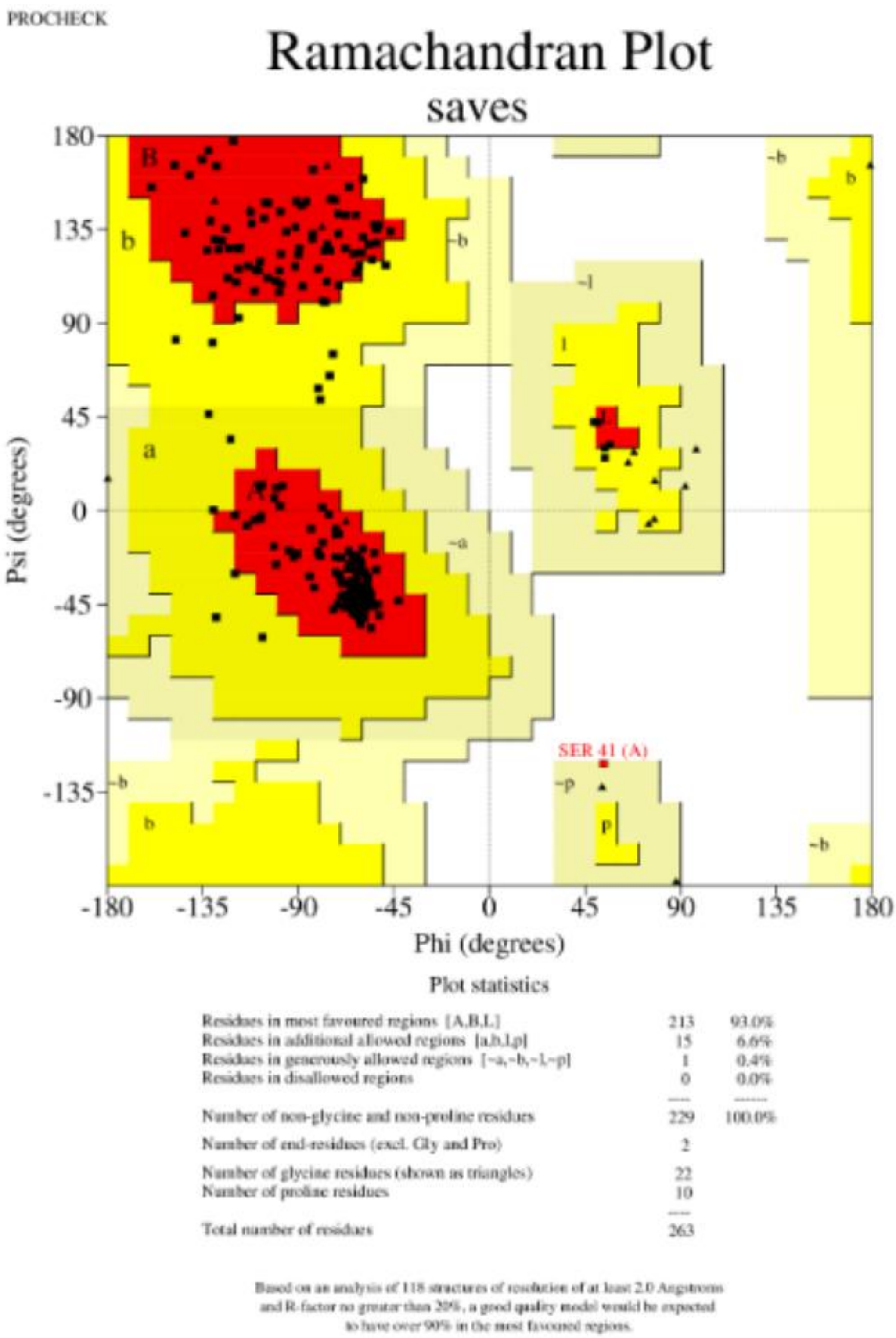

Fig. S9. Validation result of serine protease predicted by AlphaColab. (a) ERRAT, (b)VERIFY3D, and (c) PROCHECK.

<u>SWISS-MODEL:</u>

(a) ERRAT:

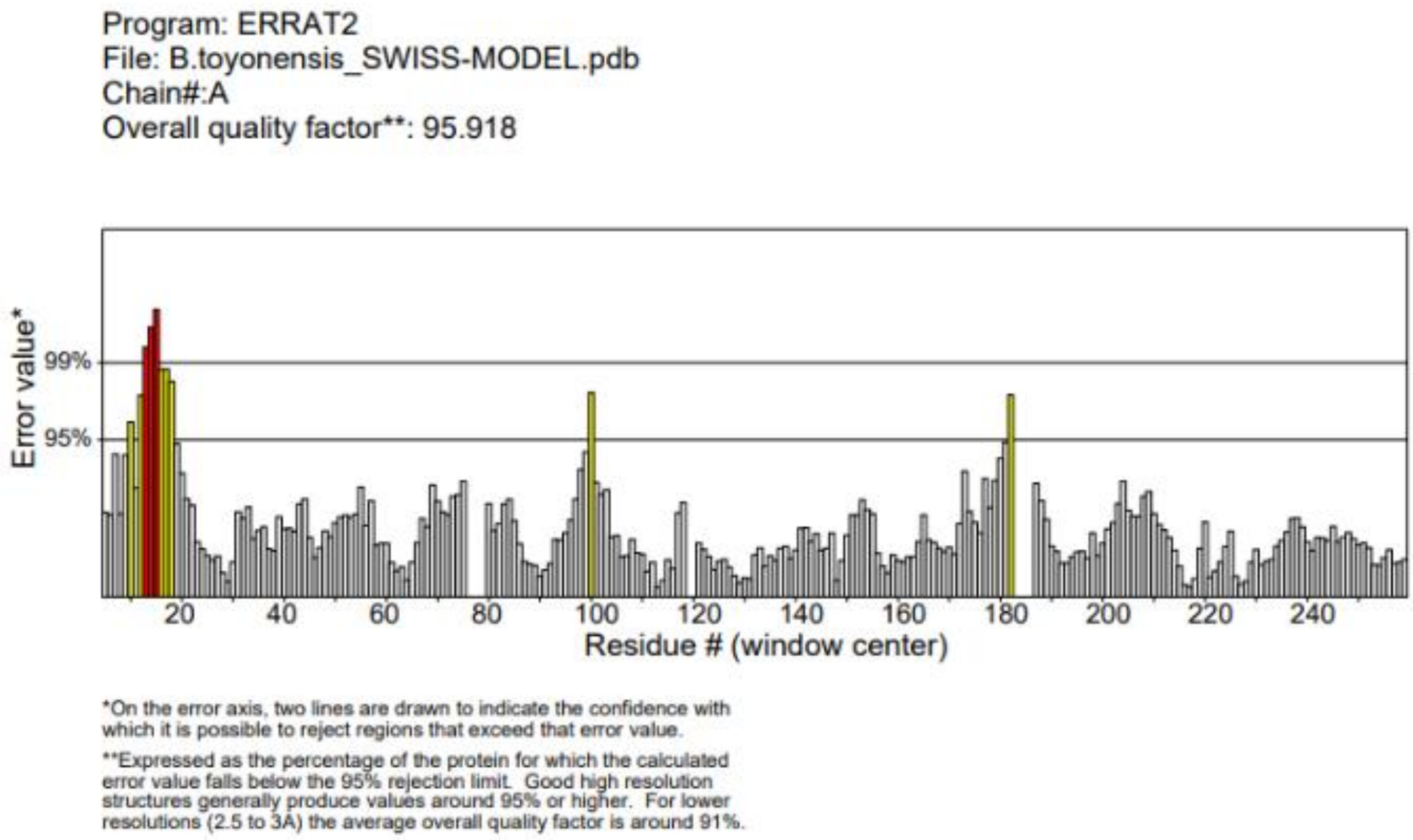

(c) VERIFY3D:

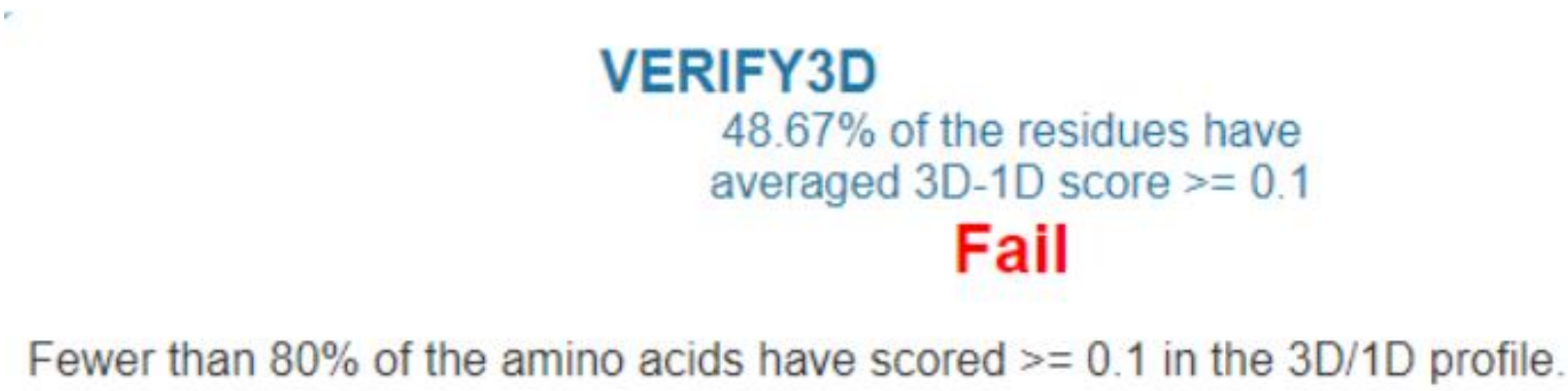

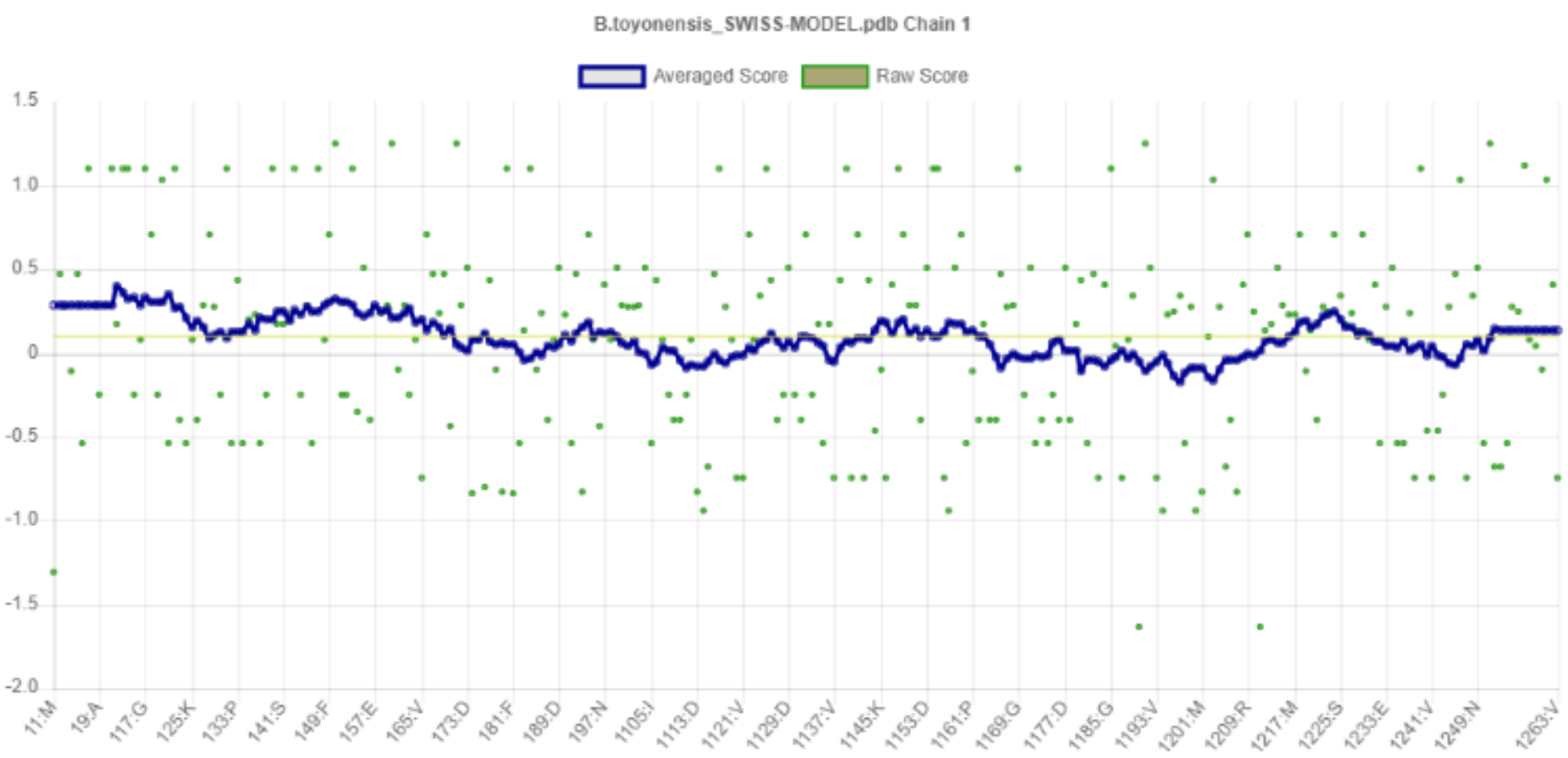

(c) PROCHECK:

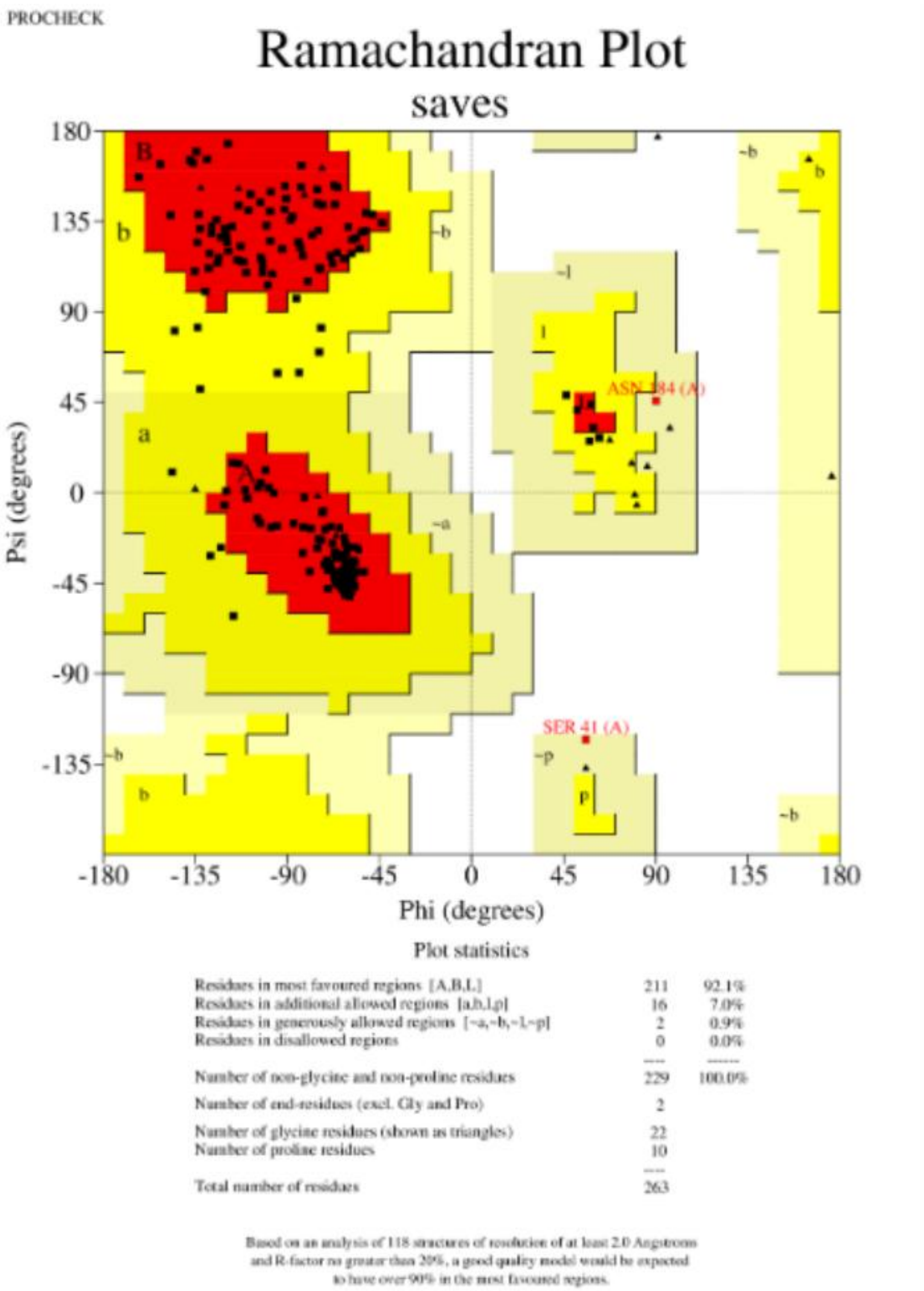

Fig. S10. Validation result of serine protease predicted by SWISS-MODEL using AlphaFold database. (a) ERRAT, (b)VERIFY3D, and (c) PROCHECK.

<u>Sequence Similarity Search:</u>

(A) PNPLA domain-containing protein

1. AFDB:J8KWL5 (*B. cereus*)

(a) ERRAT:

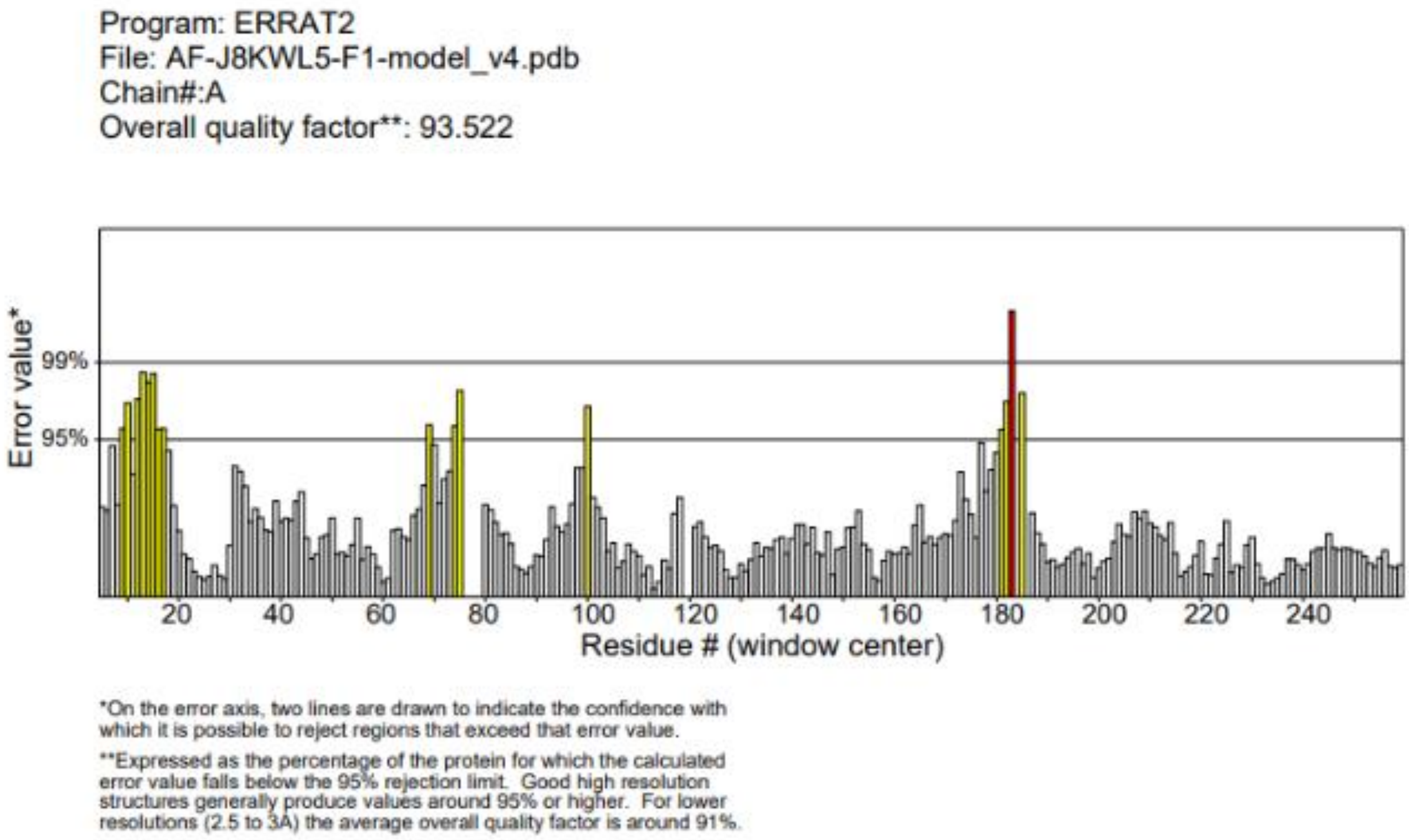

(b) VERIFY3D:

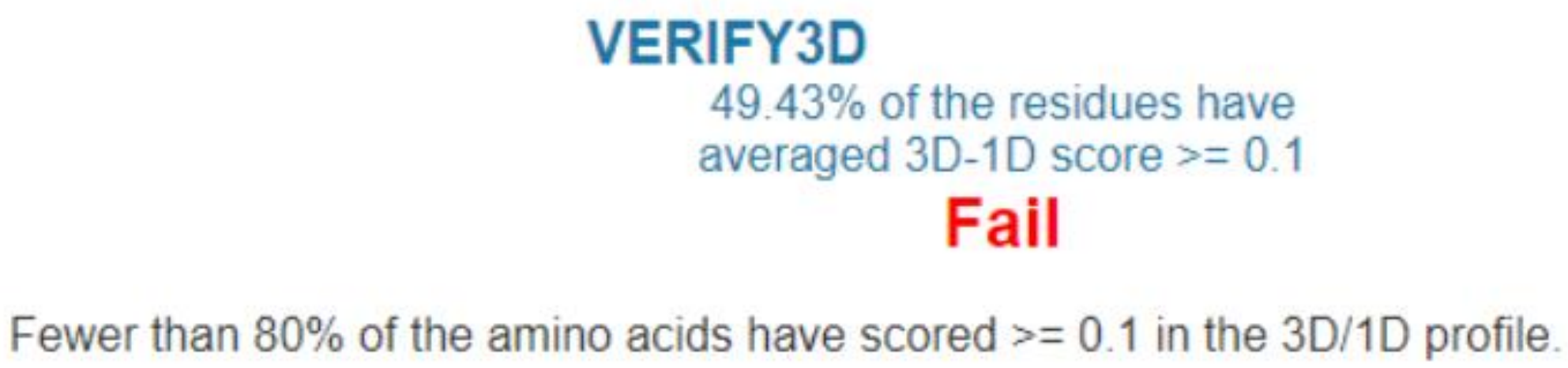

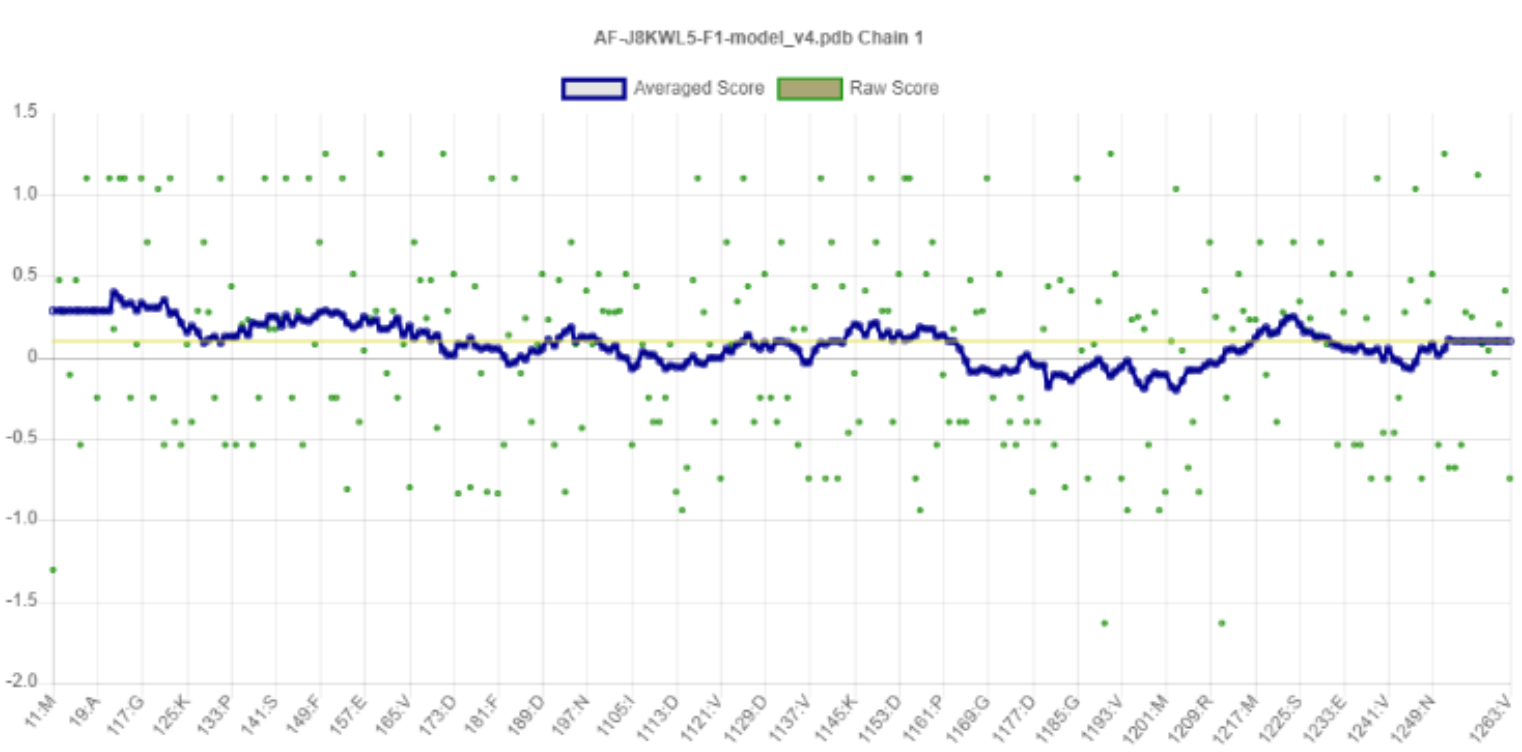

(c) PROCHECK:

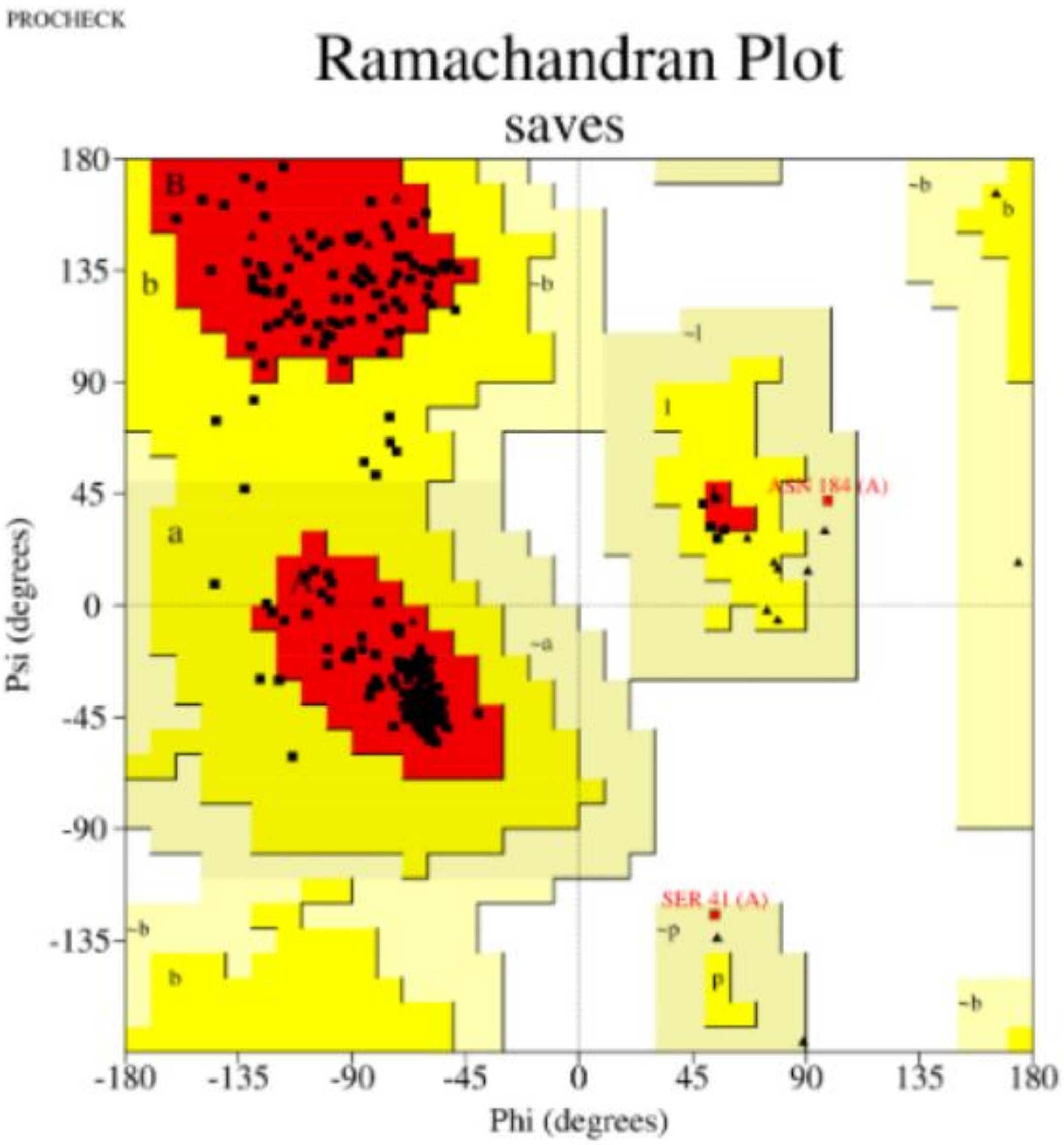

Plot statistics

Residues in most favoured regions [A,B,L]	213	93.0%
Residues in additional allowed regions [a,b,l,p]	14	6.1%
Residues in generously allowed regions [~a,~b,~l,~p]	2	0.9%
Residues in disallowed regions	0	0.0%
	----	------
Number of non-glycine and non-proline residues	229	100.0%
Number of end-residues (excl. Gly and Pro)	2	
Number of glycine residues (shown as triangles)	22	
Number of proline residues	10	

Total number of residues	263	

Based on an analysis of 118 structures of resolution of at least 2.0 Angstroms and R-factor no greater than 20%, a good quality model would be expected to have over 90% in the most favoured regions.

Fig. S11. Validation result of serine protease predicted by Sequence Similarity Search using AFDB:J8KWL5 as template. (a) ERRAT, (b)VERIFY3D, and (c) PROCHECK.

2. AFDB:A0A2I0RDH6 (*B. cereus*)

(a) ERRAT:

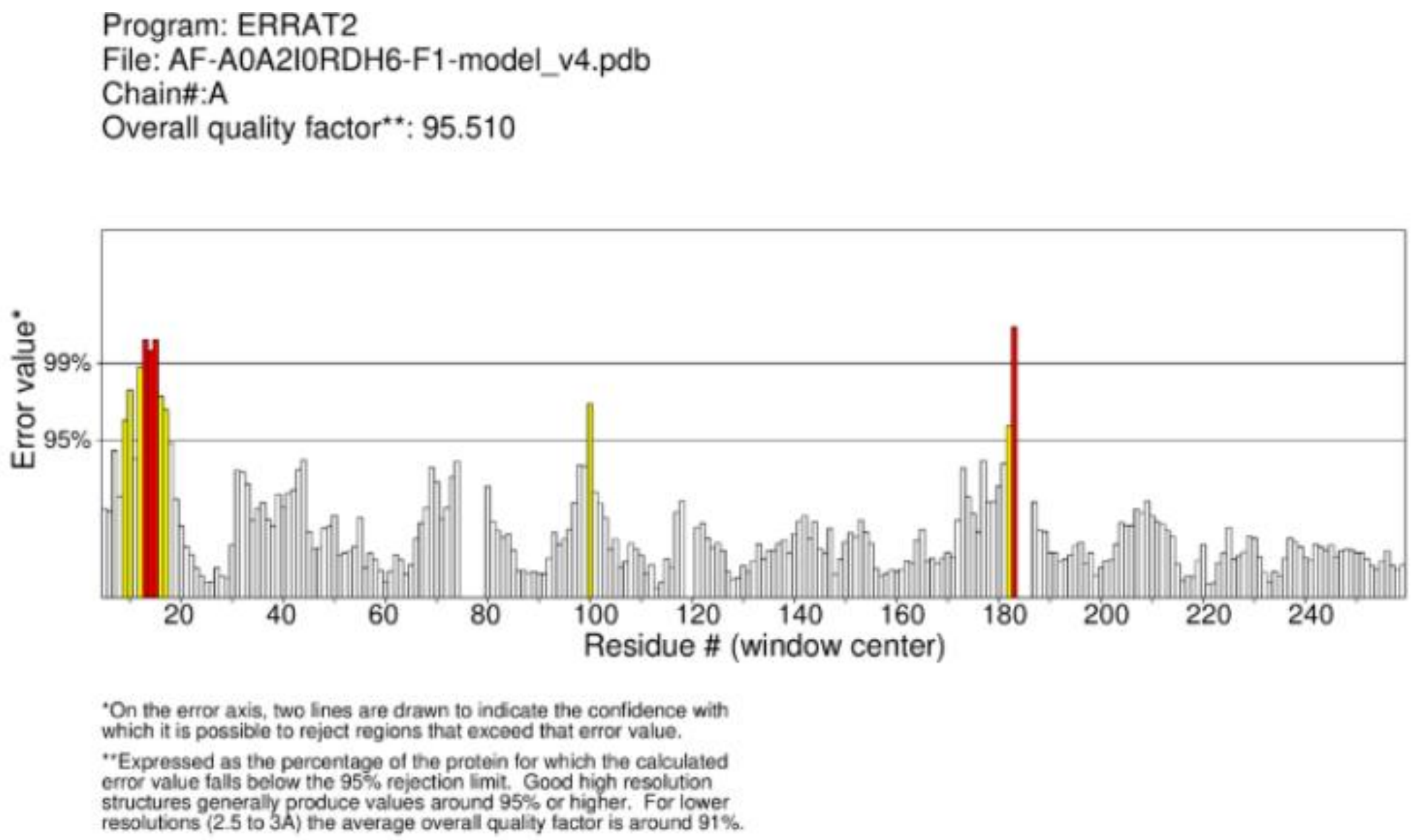

(b) VERIFY3D:

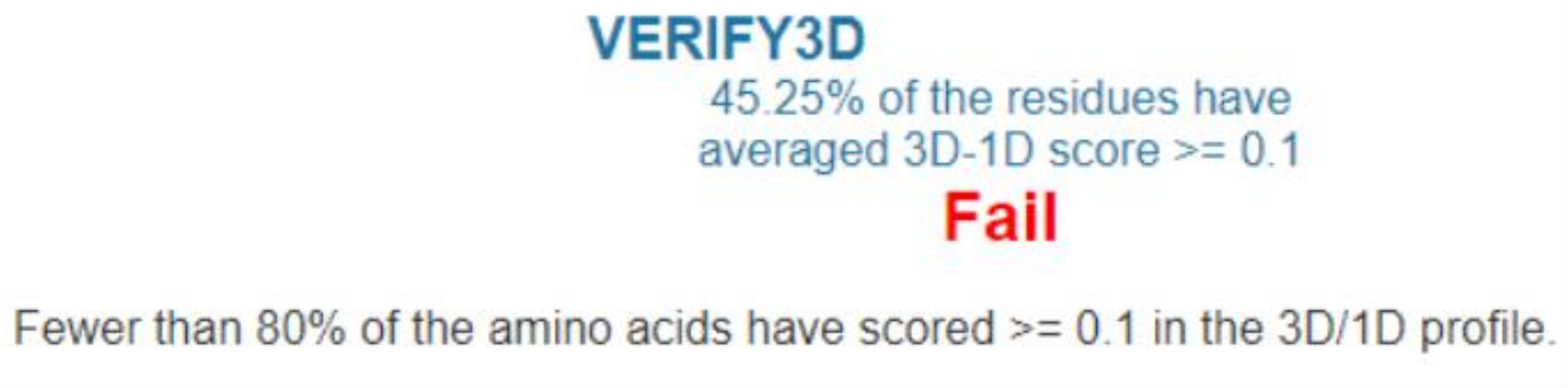

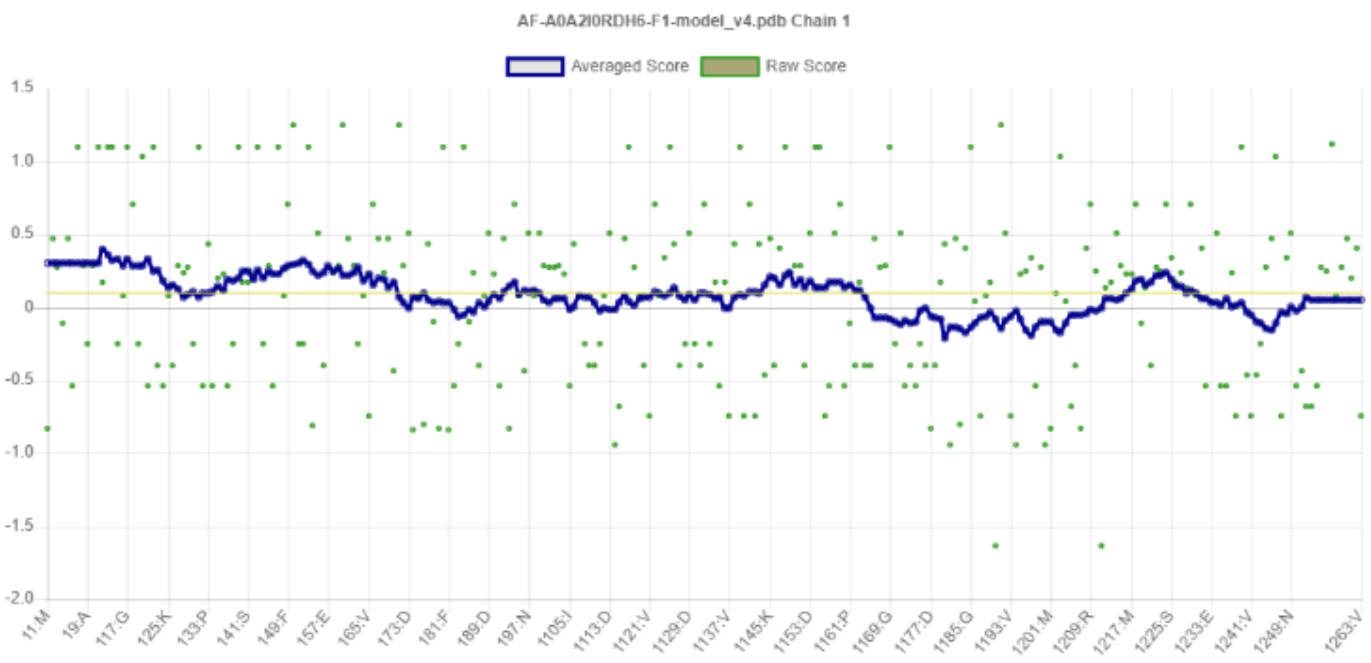
AF-A0A2I0RDH6-F1-model_v4.pdb Chain 1
Averaged Score
Raw Score
1.5
1.0
0.5
0
-0.5
-1.0
-1.5
-2.0

(c) PROCHECK:

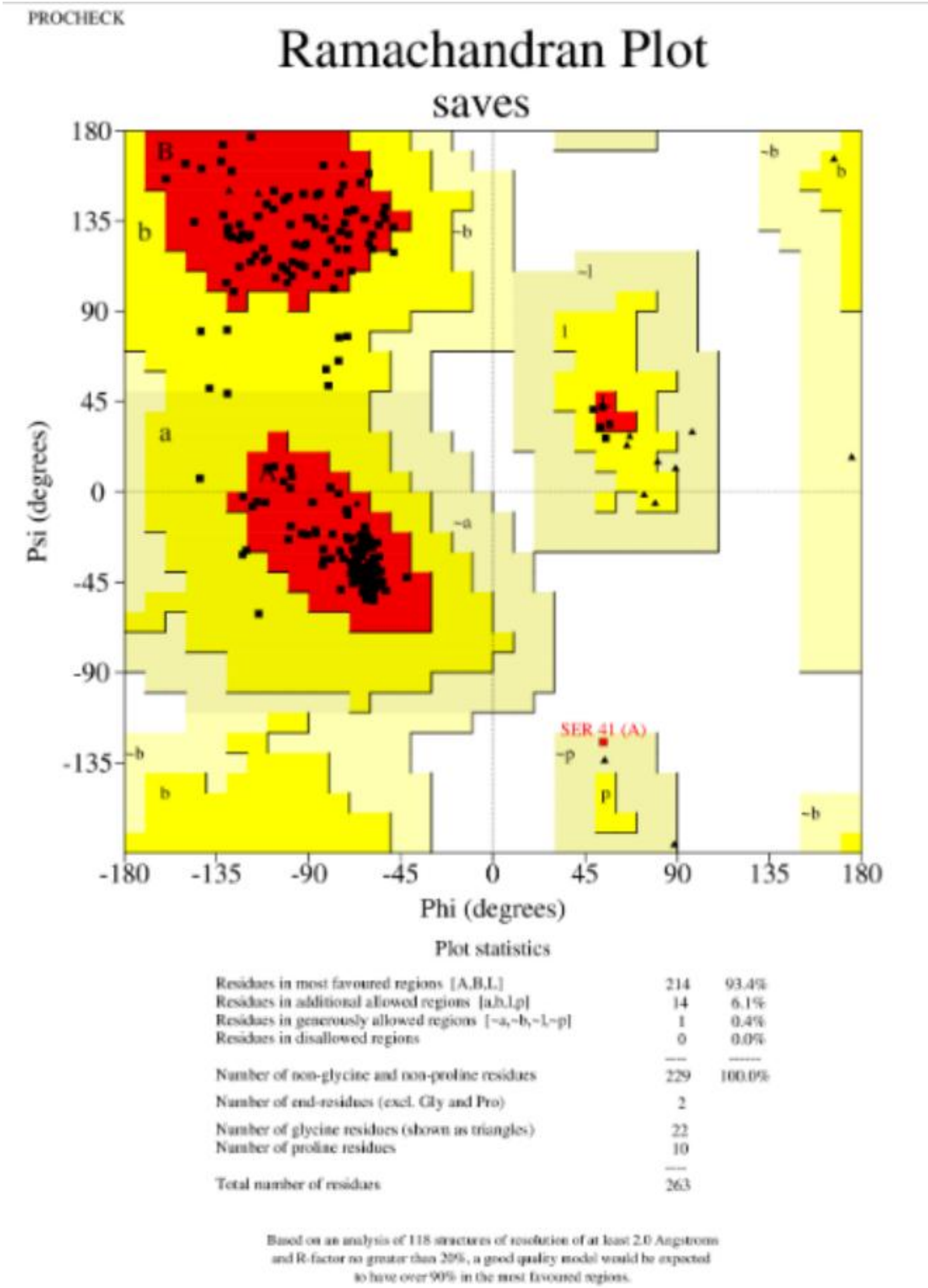

Fig. S12. Validation result of serine protease predicted by Sequence Similarity Search using AFDB:A0A2I0RDH6 as template. (a) ERRAT, (b)VERIFY3D, and (c) PROCHECK.

(B) Patatin family protein

1. AFDB:A0A345XC57 (*Bacillus sp.)*

(a) ERRAT:

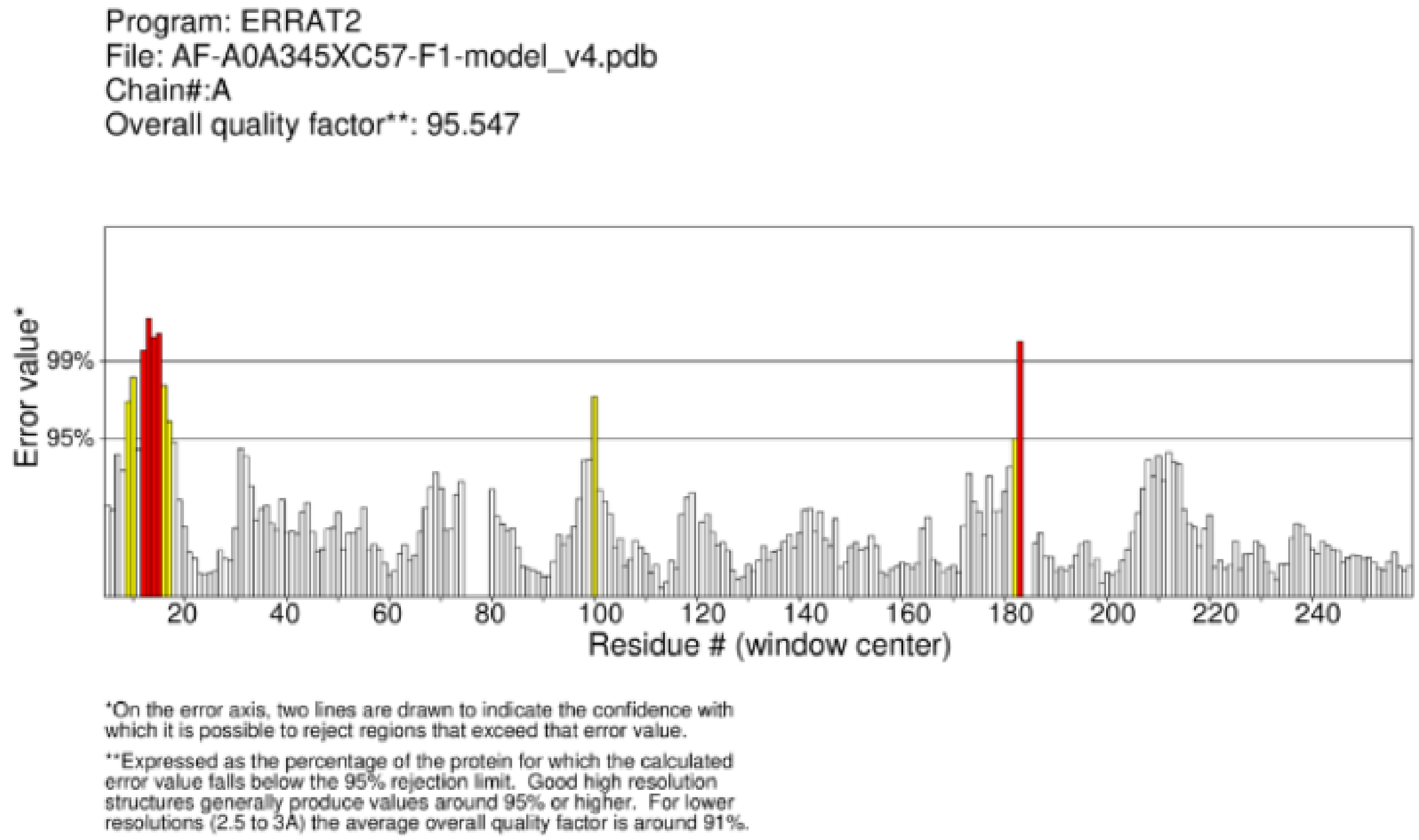

(b) VERIFY3D:

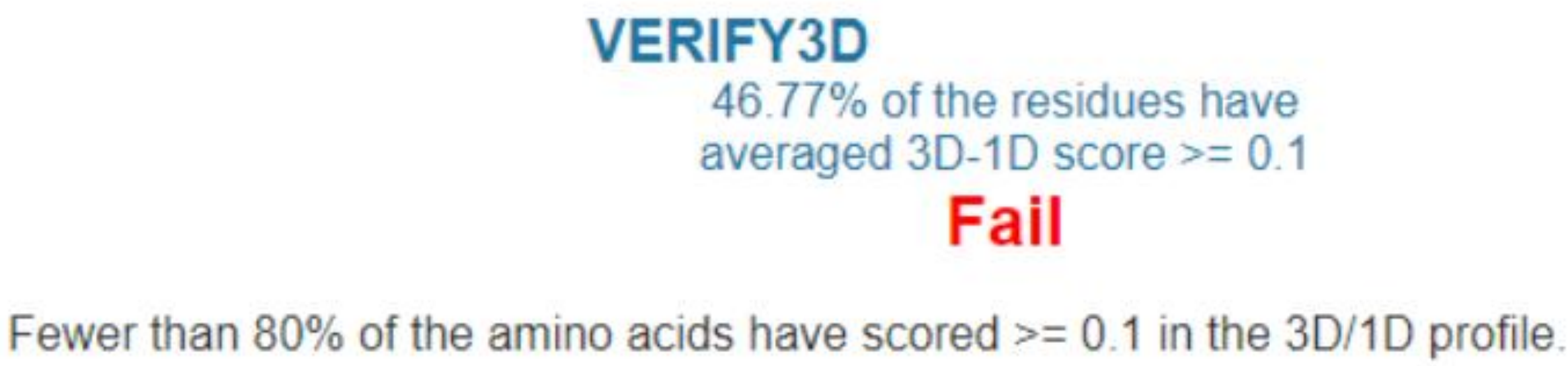

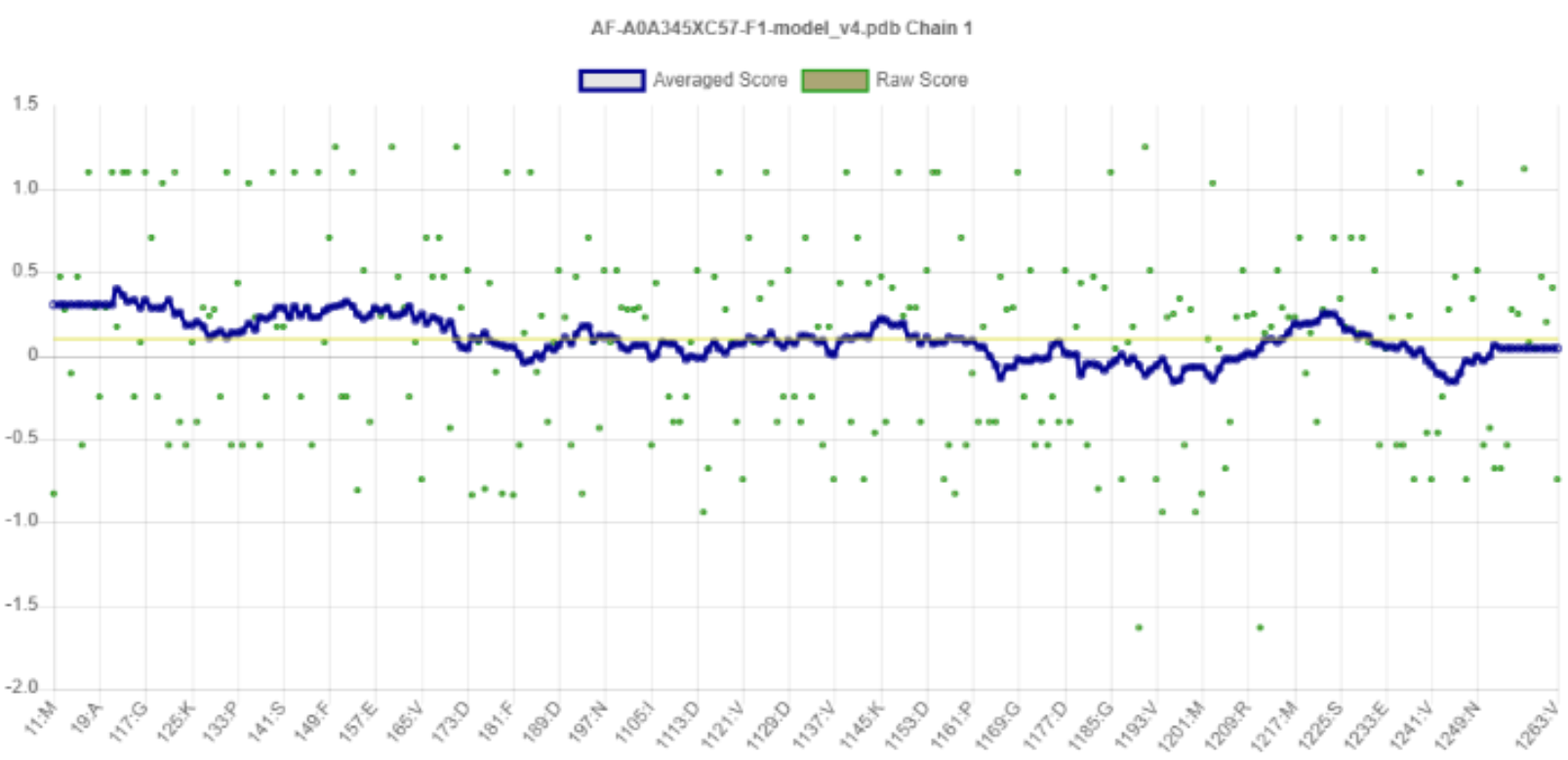

(c) PROCHECK:

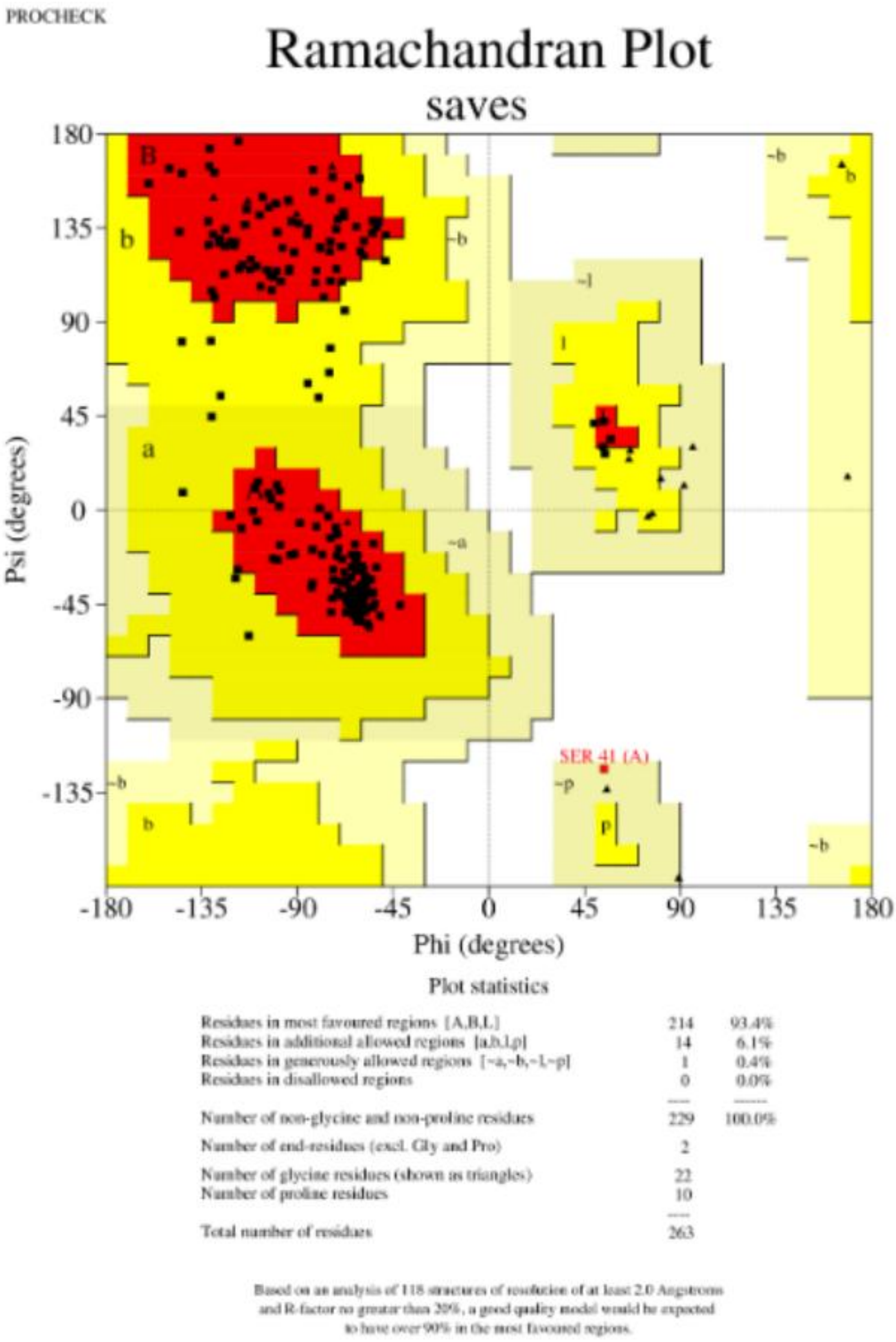

Fig. S13. Validation result of serine protease predicted by Sequence Similarity Search using AFDB:A0A345XC57 as template. (a) ERRAT, (b)VERIFY3D, and (c) PROCHECK.

2. AFDB:A0A7T3JG97 (*B. thuringiensis*)

(a) ERRAT:

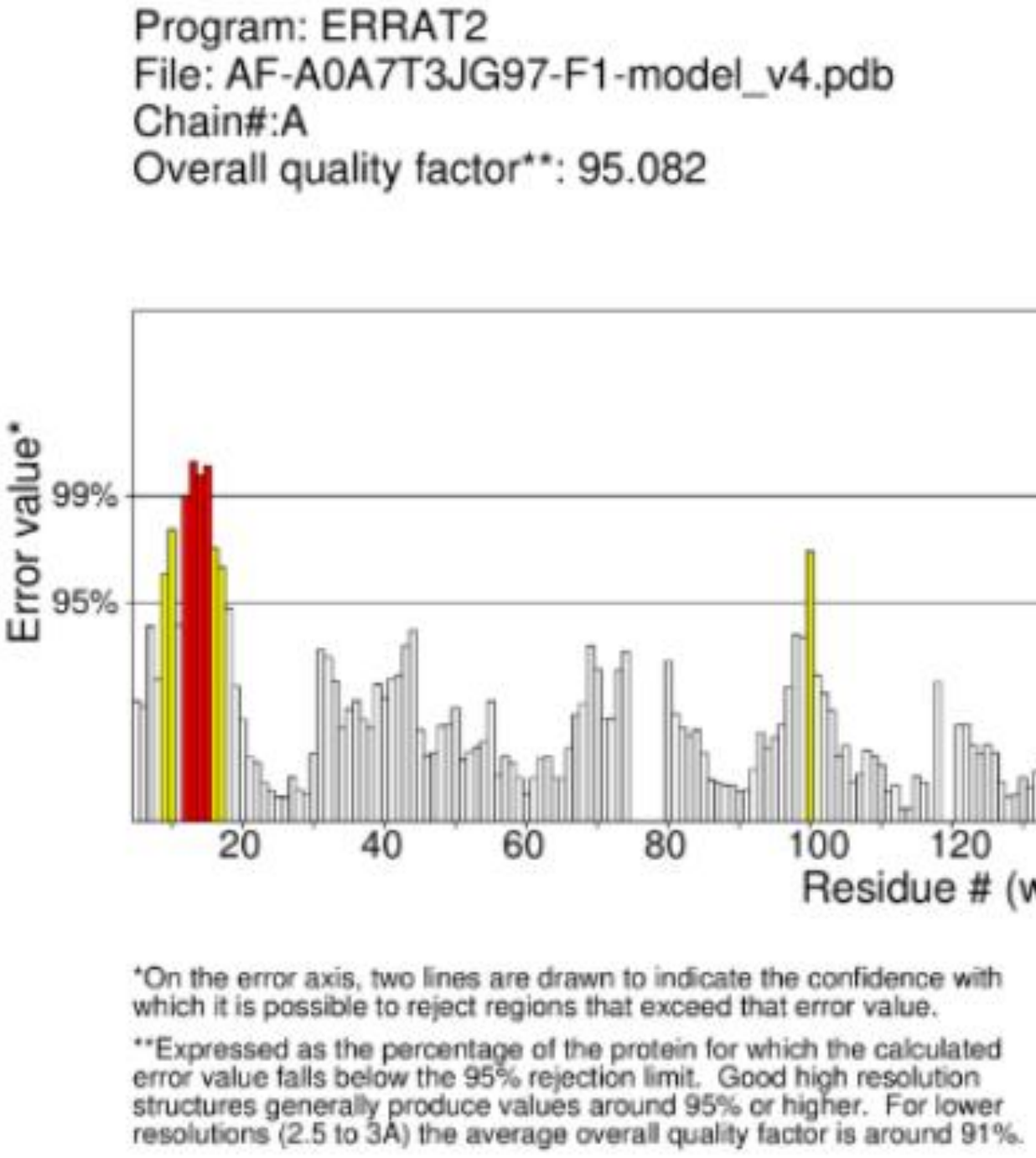

(b) VERIFY3D:

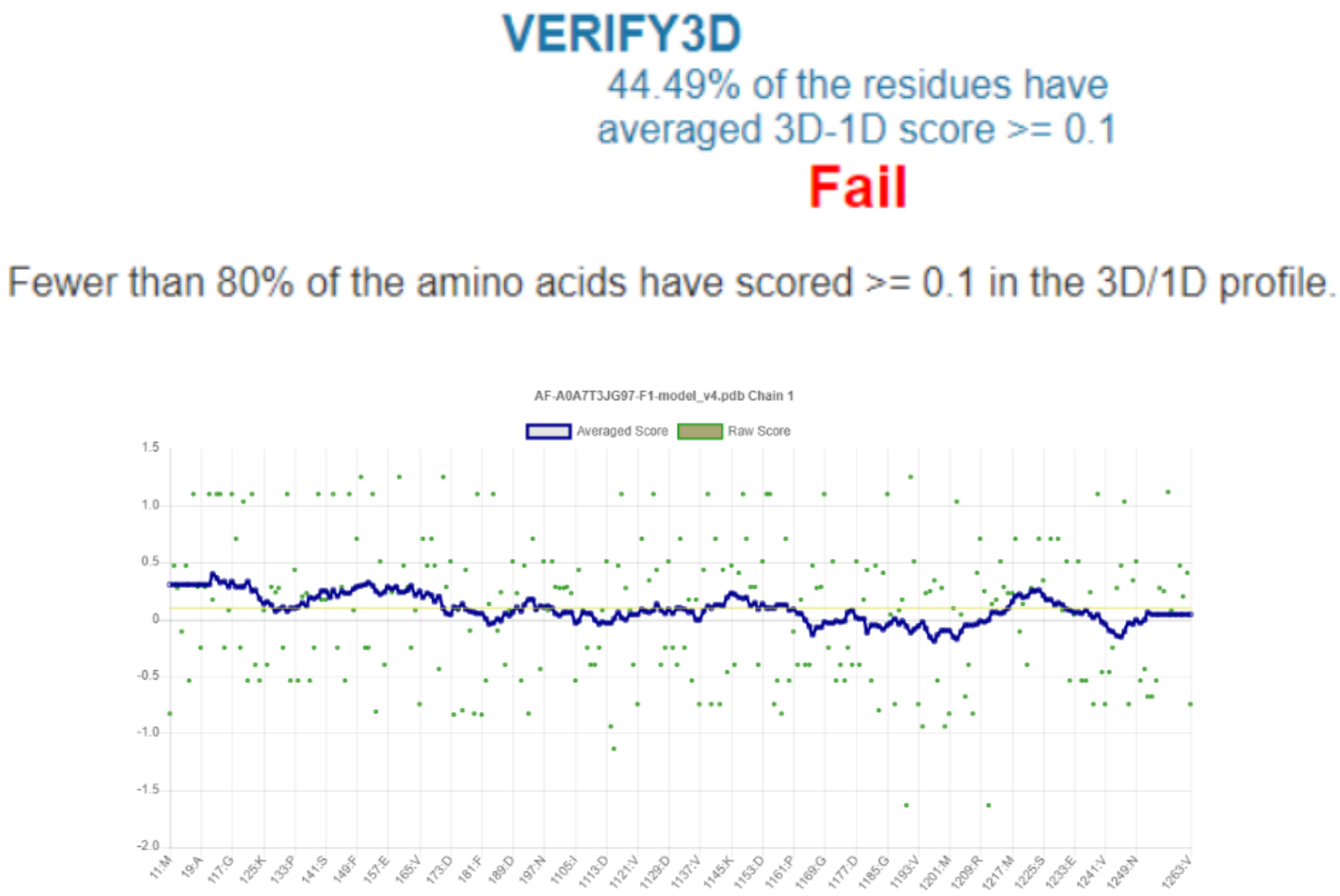

(c) PROCHECK:

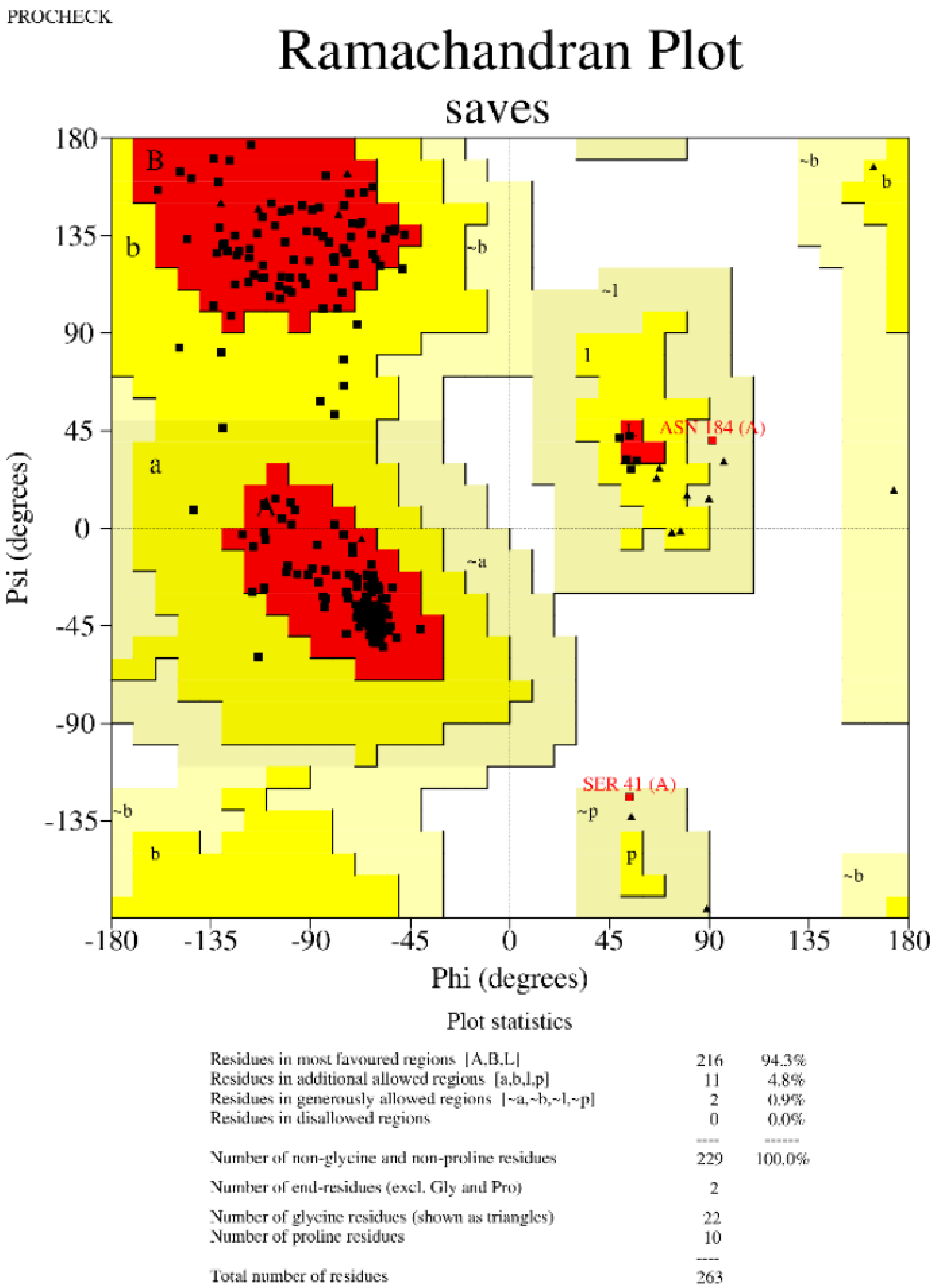

Fig. S14. Validation result of serine protease predicted by Sequence Similarity Search using AFDB:A0A7T3JG97 as template. (a) ERRAT, (b)VERIFY3D, and (c) PROCHECK.

(C) Esterase

1. AFDB:A0A242WPL1(*B. thuringiensis*)

(a) ERRAT:

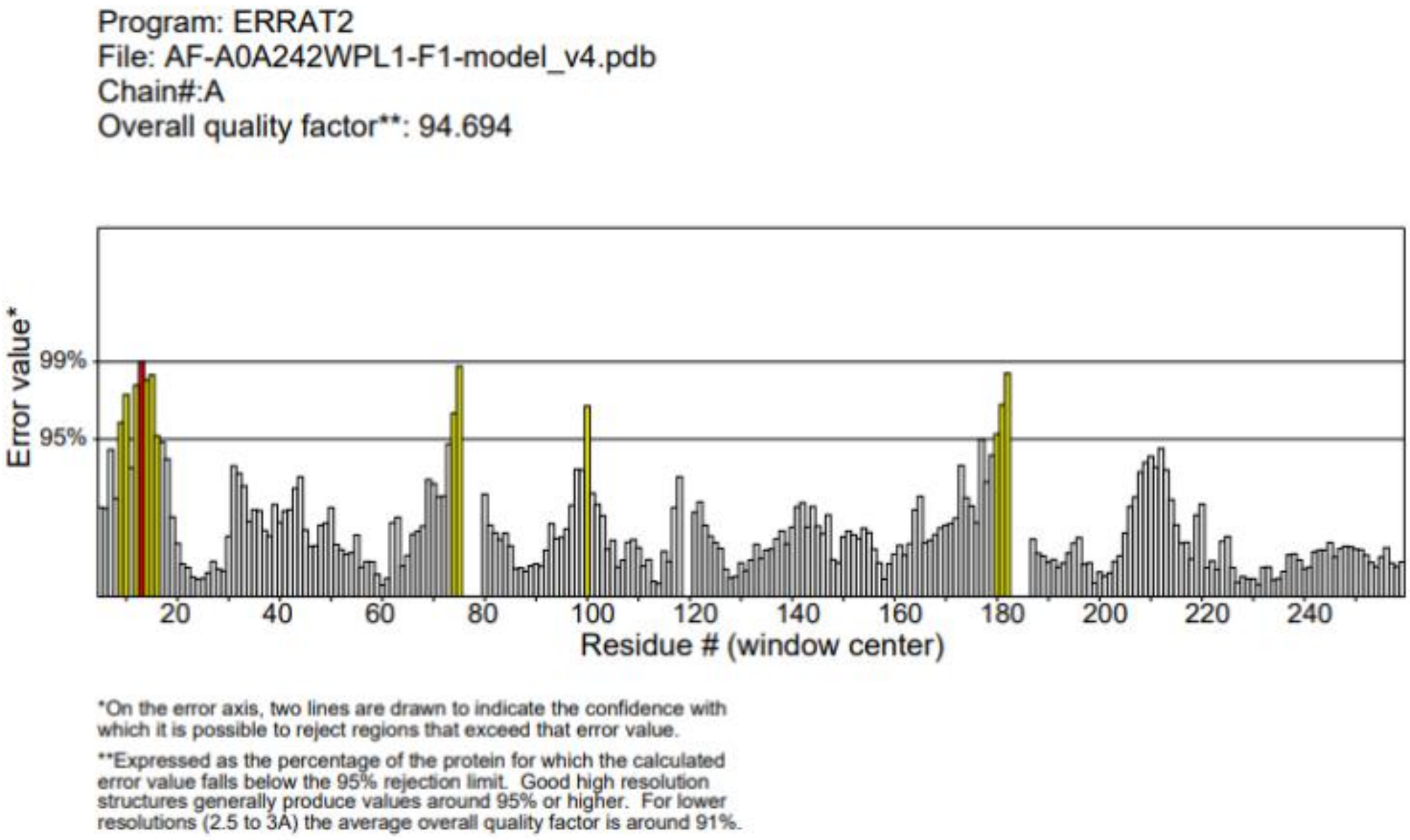

(b) VERIFY3D:

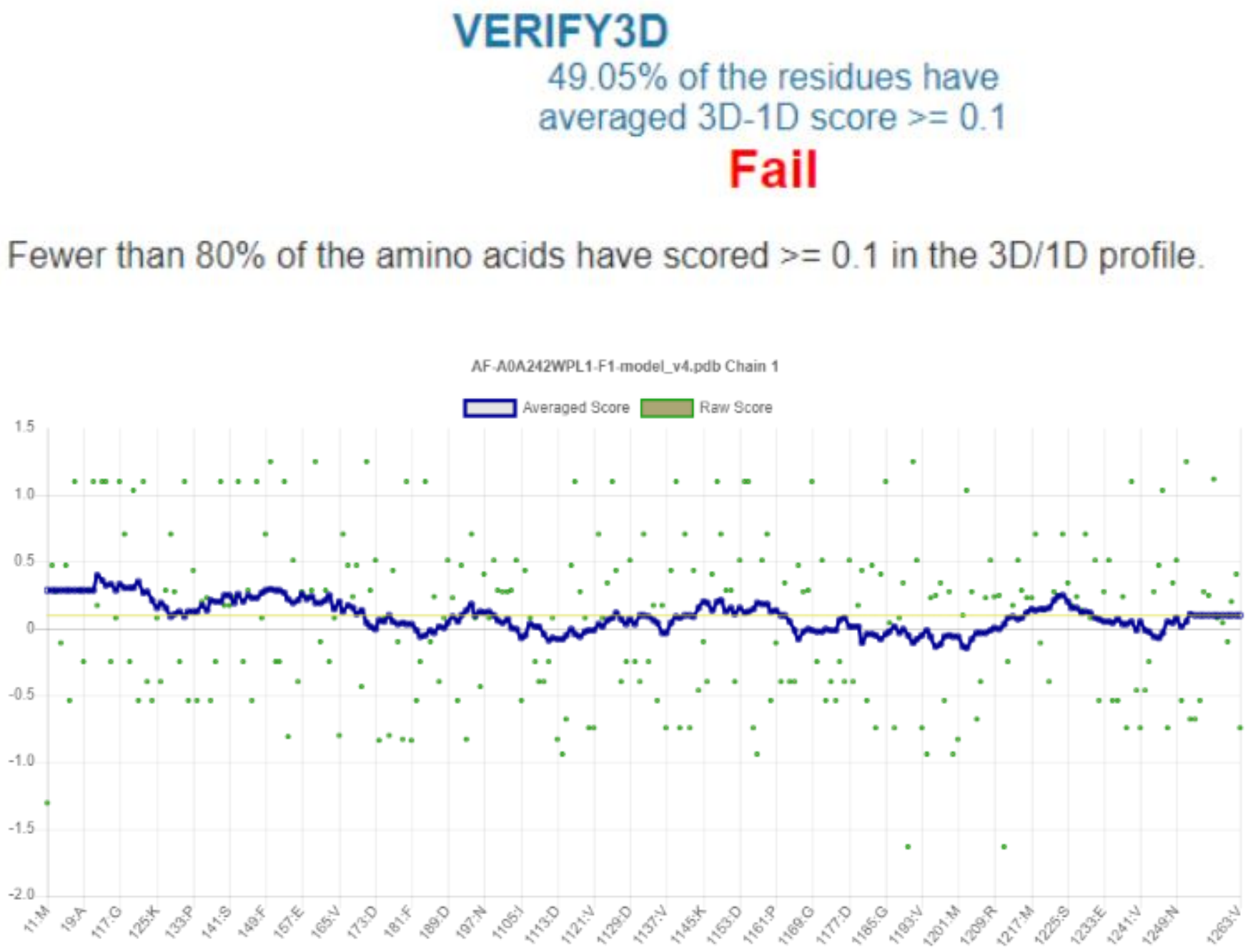

(c) PROCHECK:

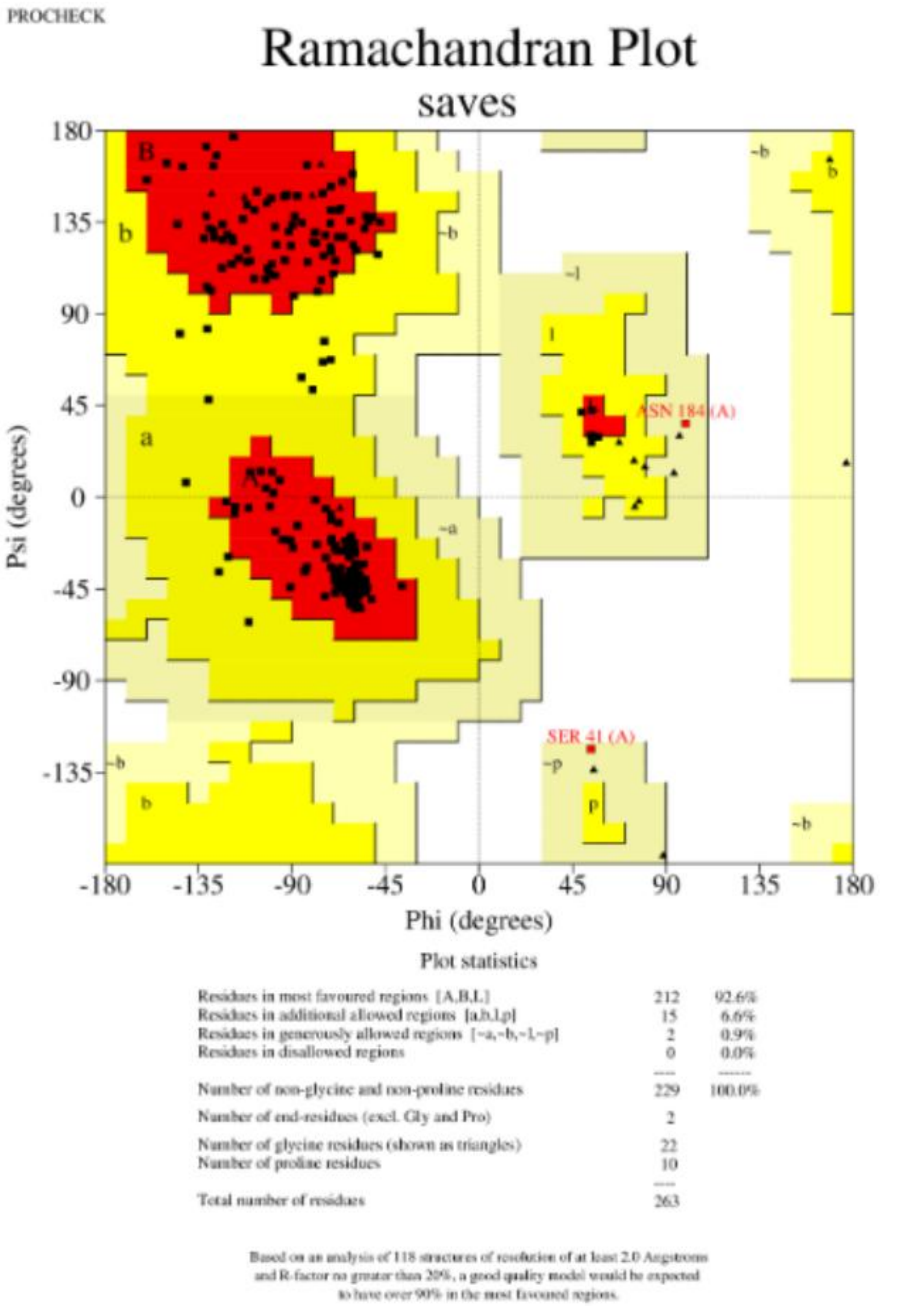

Fig. S15. Validation result of serine protease predicted by Sequence Similarity Search using AFDB:A0A242WPL1 as template. (a) ERRAT, (b)VERIFY3D, and (c) PROCHECK.

2. AFDB:A0A1V6LKE9(*B. toyonensis*)

(a) ERRAT:

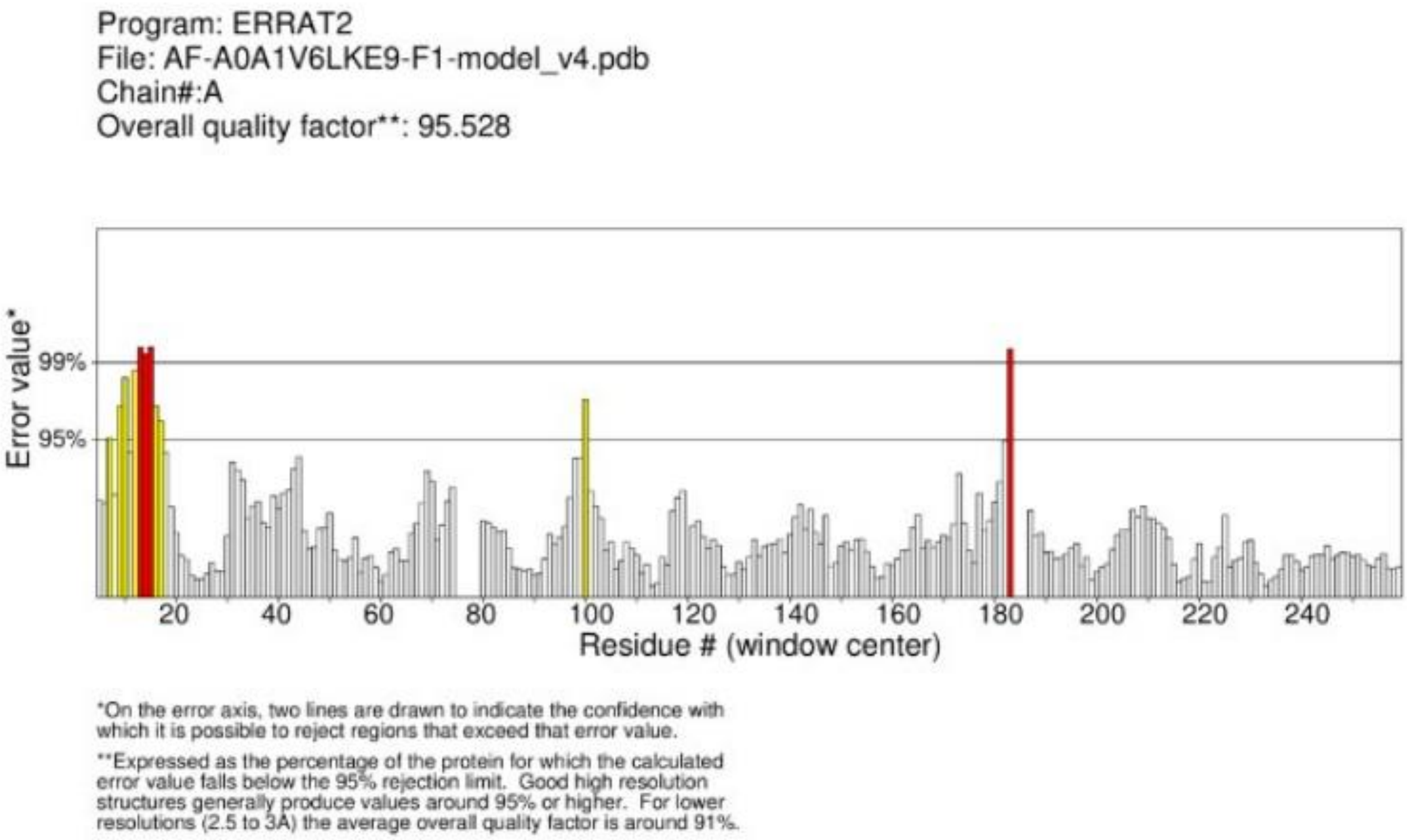

(b) VERIFY3D:

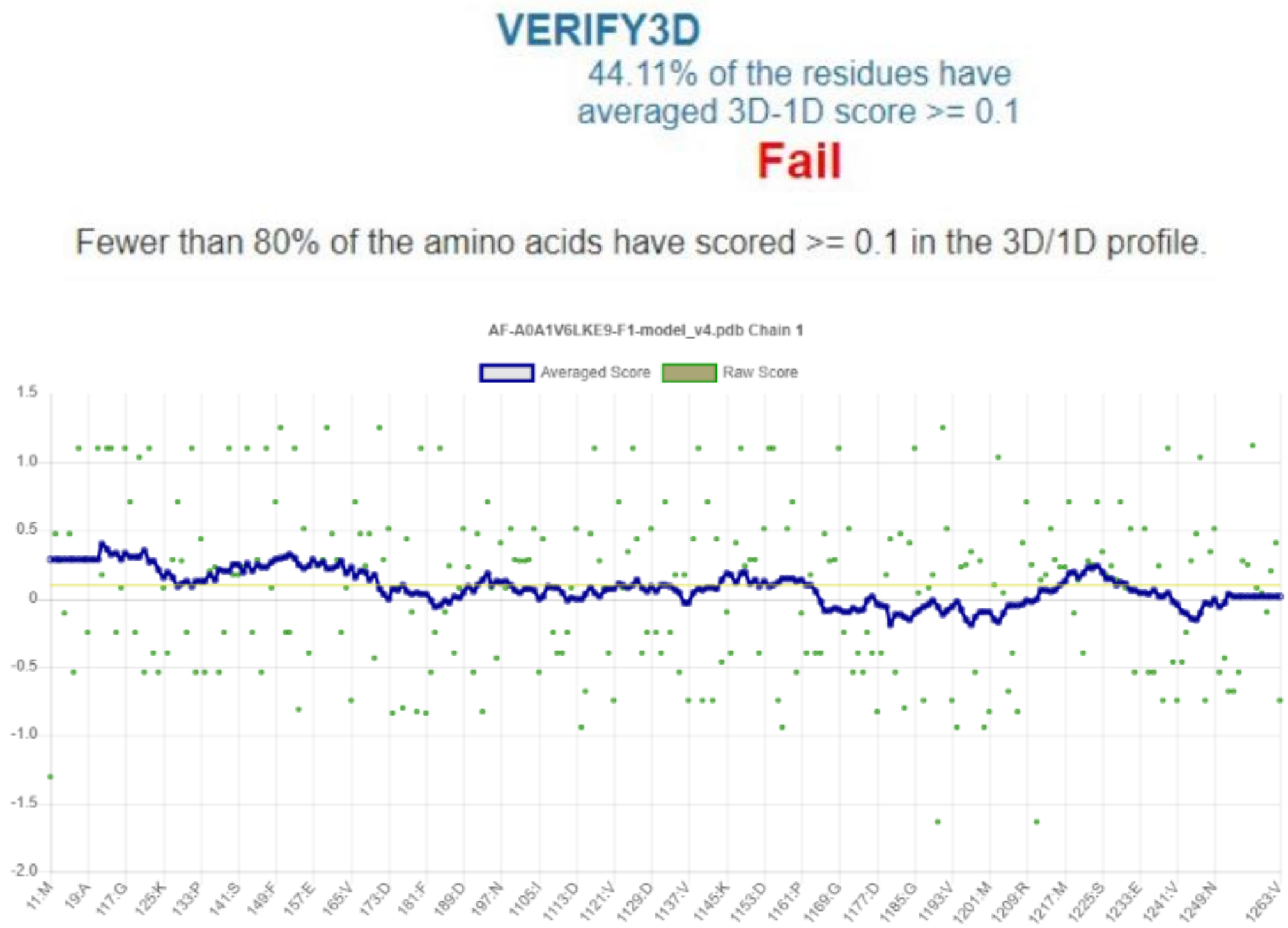

(c) PROCHECK:

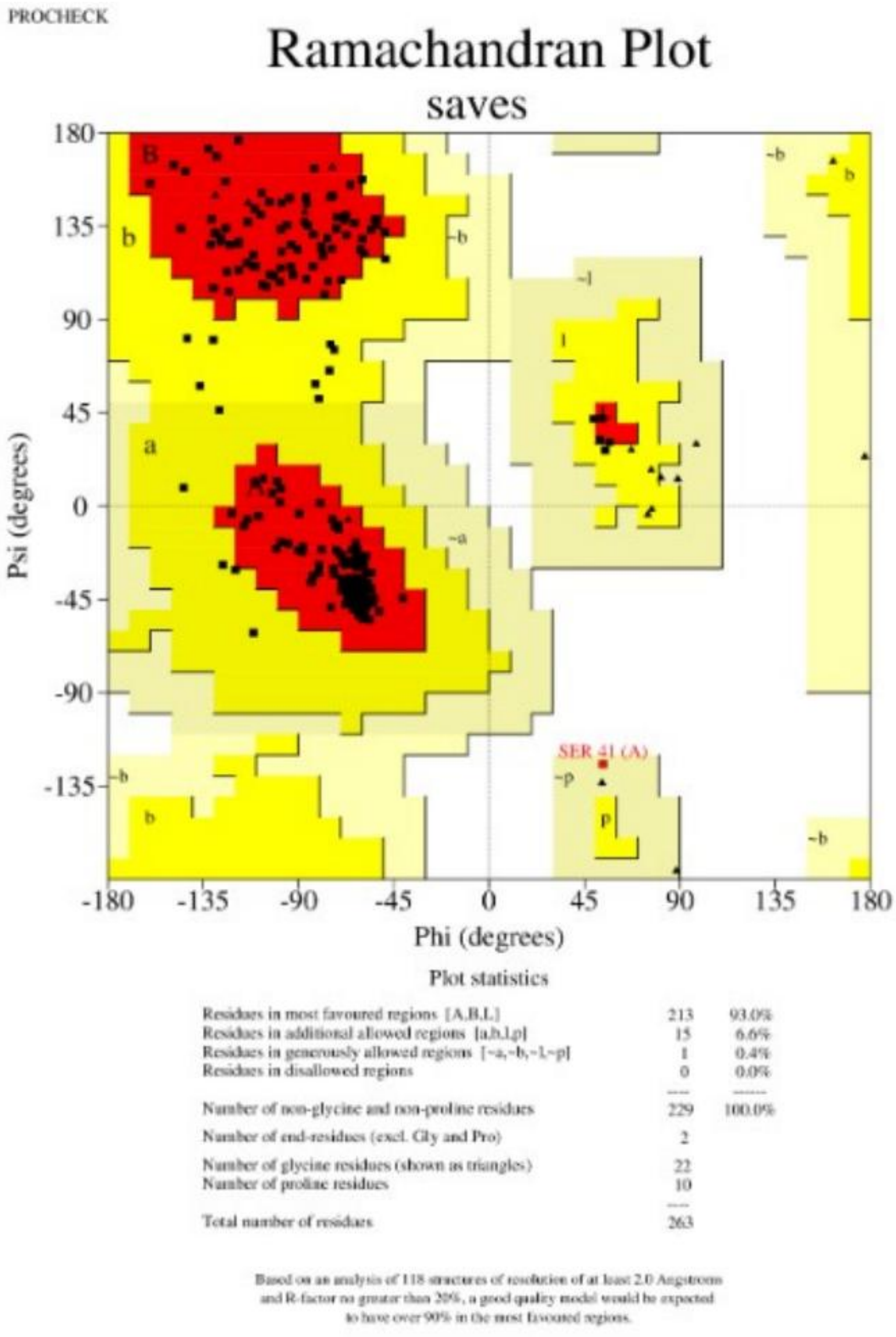

Fig. S16. Validation result of serine protease predicted by Sequence Similarity Search using AFDB:A0A1V6LKE9 as template. (a) ERRAT, (b)VERIFY3D, and (c) PROCHECK.

3. AFDB:A0A5K6LDT8(*B. sp.)*

(a) ERRAT:

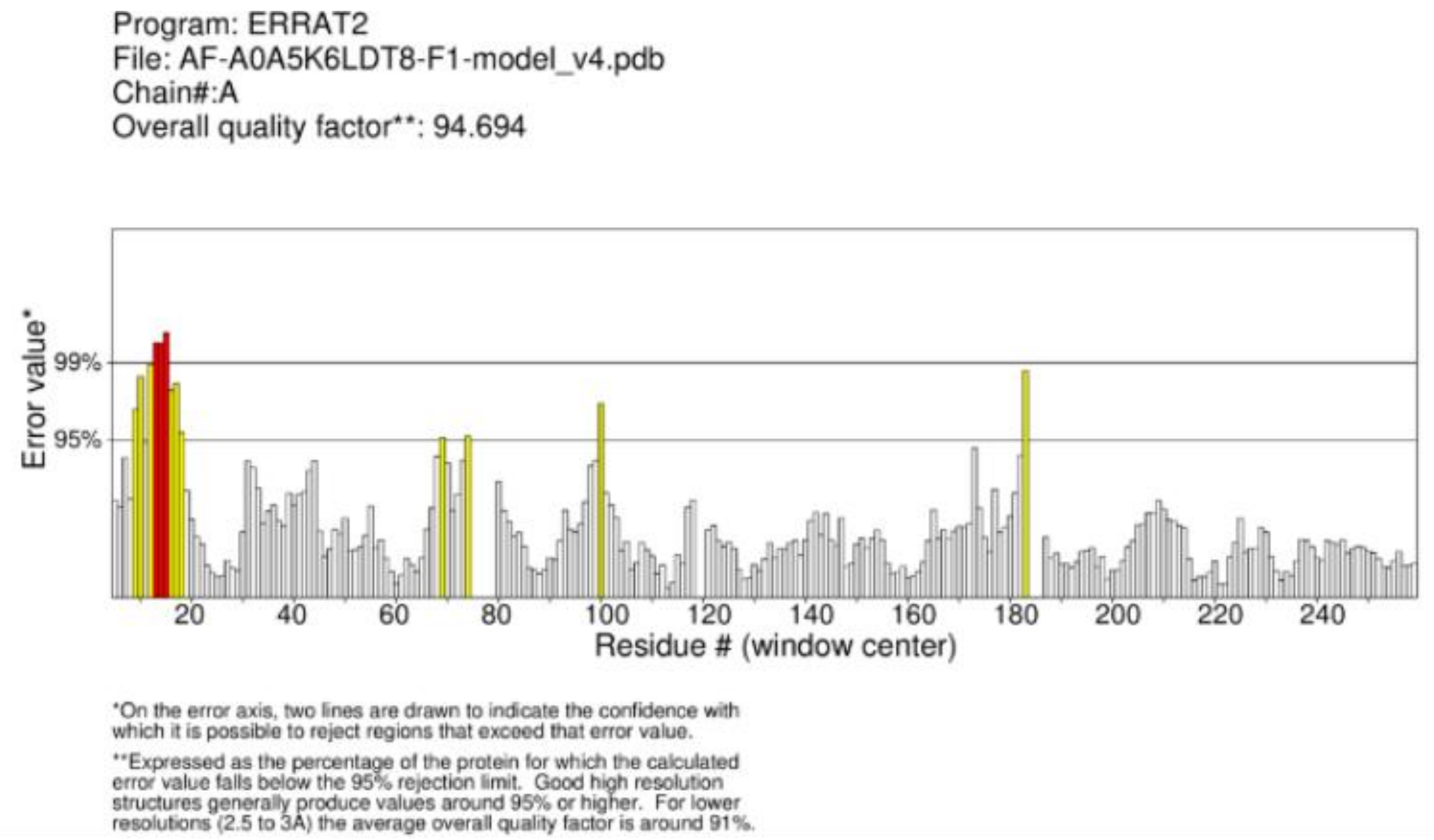

(b) VERIFY3D:

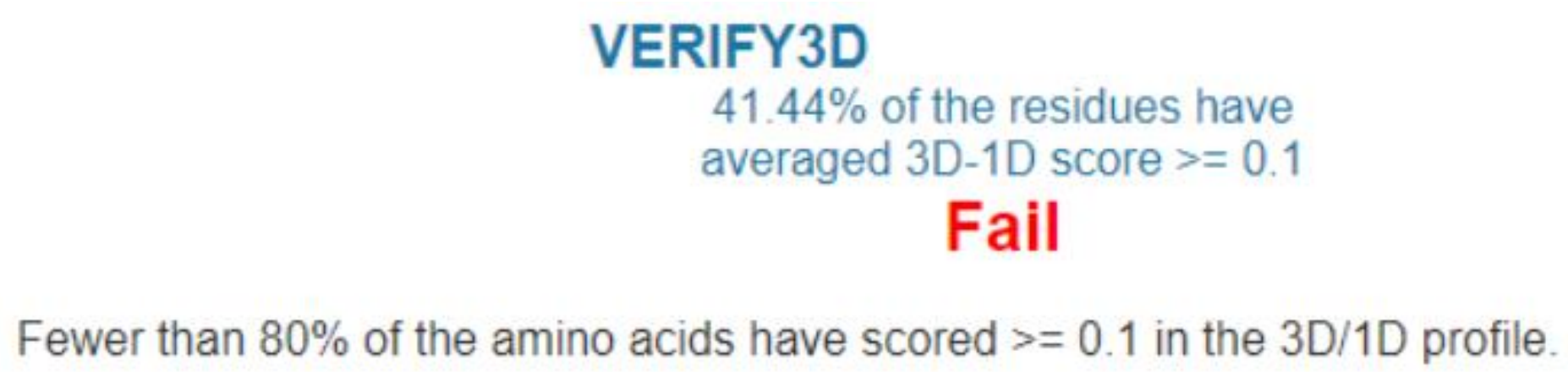

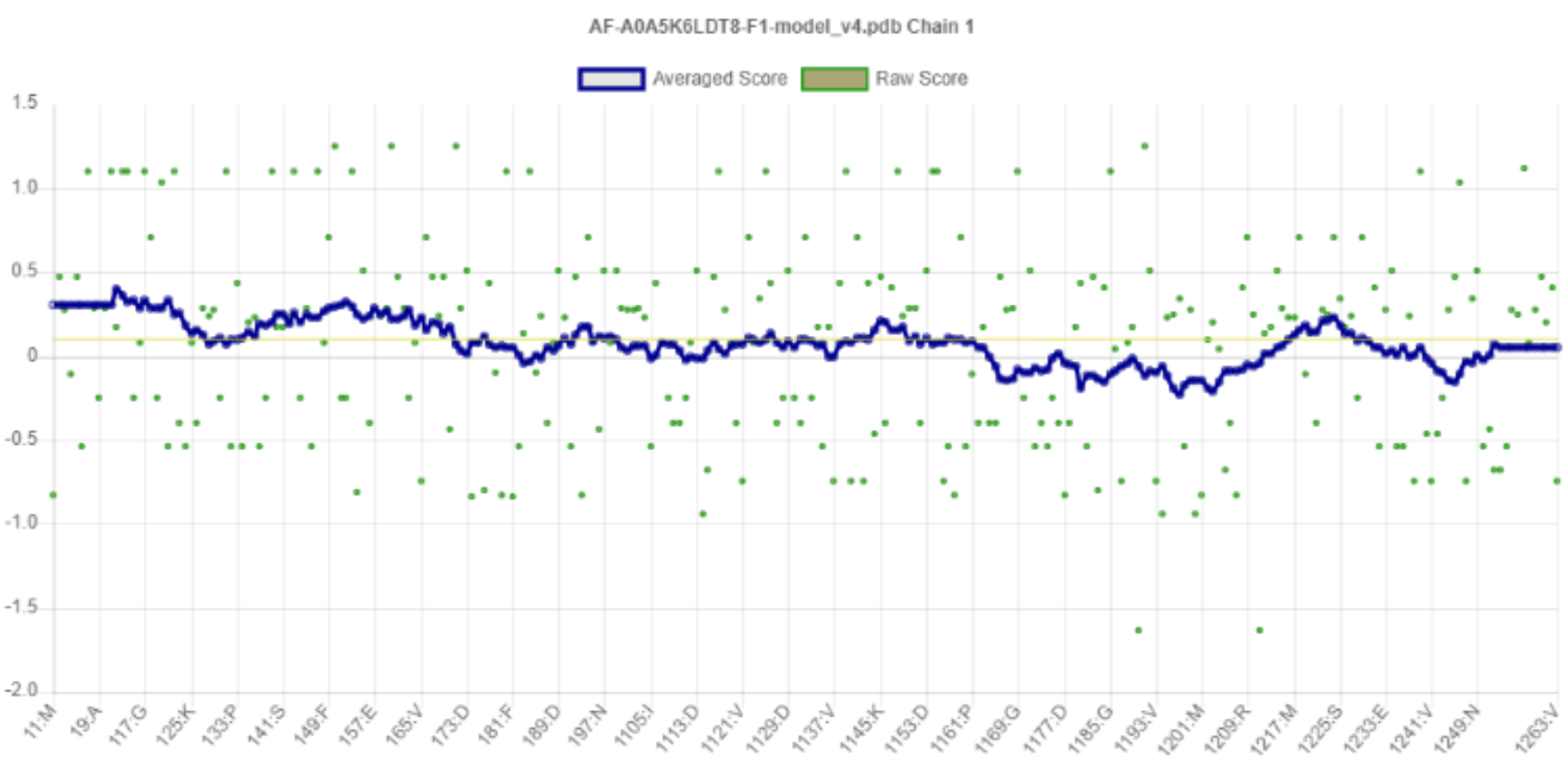

(c) PROCHECK:

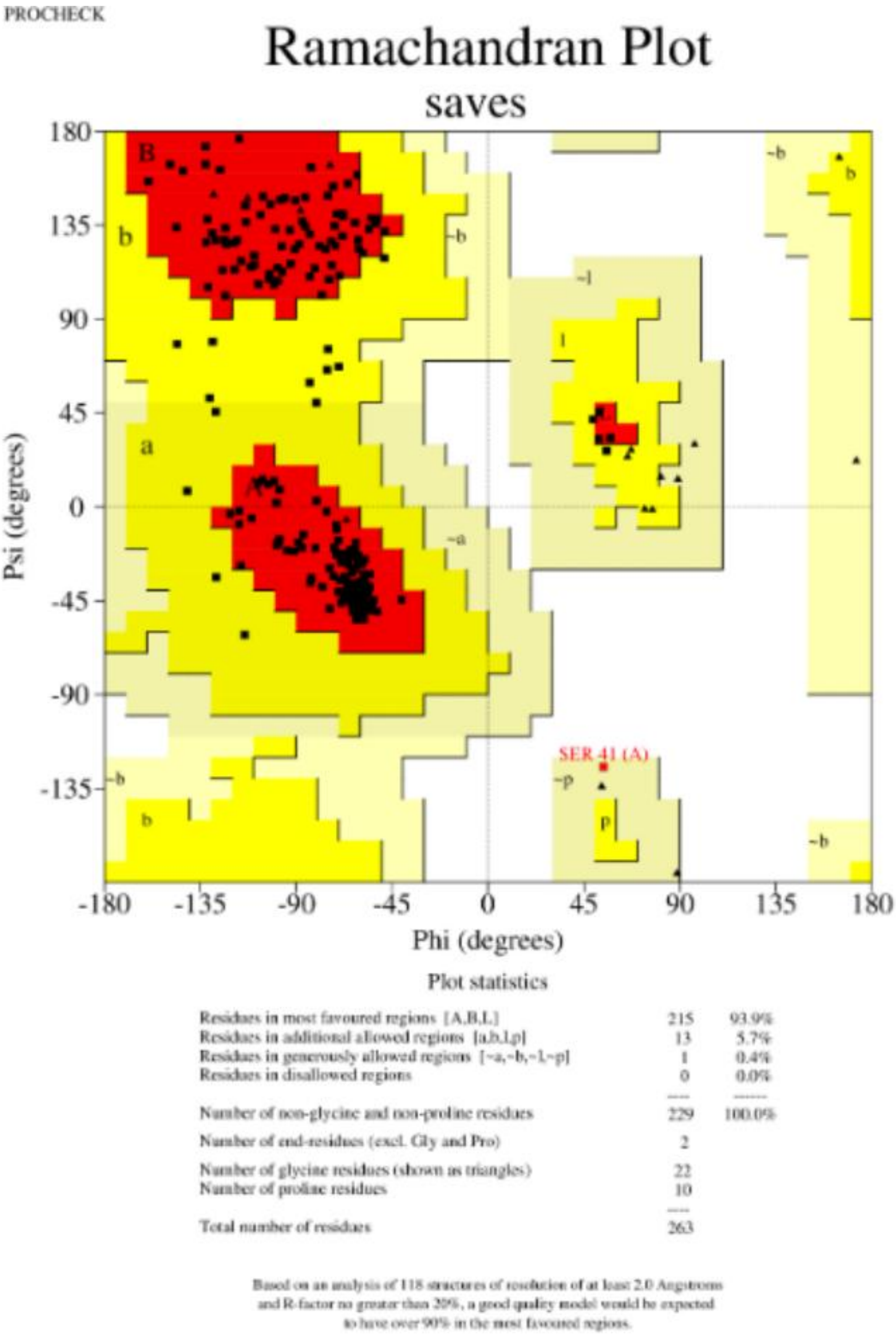

Fig. S17. Validation result of serine protease predicted by Sequence Similarity Search using AFDB:A0A5K6LDT8 as template. (a) ERRAT, (b)VERIFY3D, and (c) PROCHECK.

4. AFDB:A0A854KVS4(*B. thuringiensis*)

(a) ERRAT:

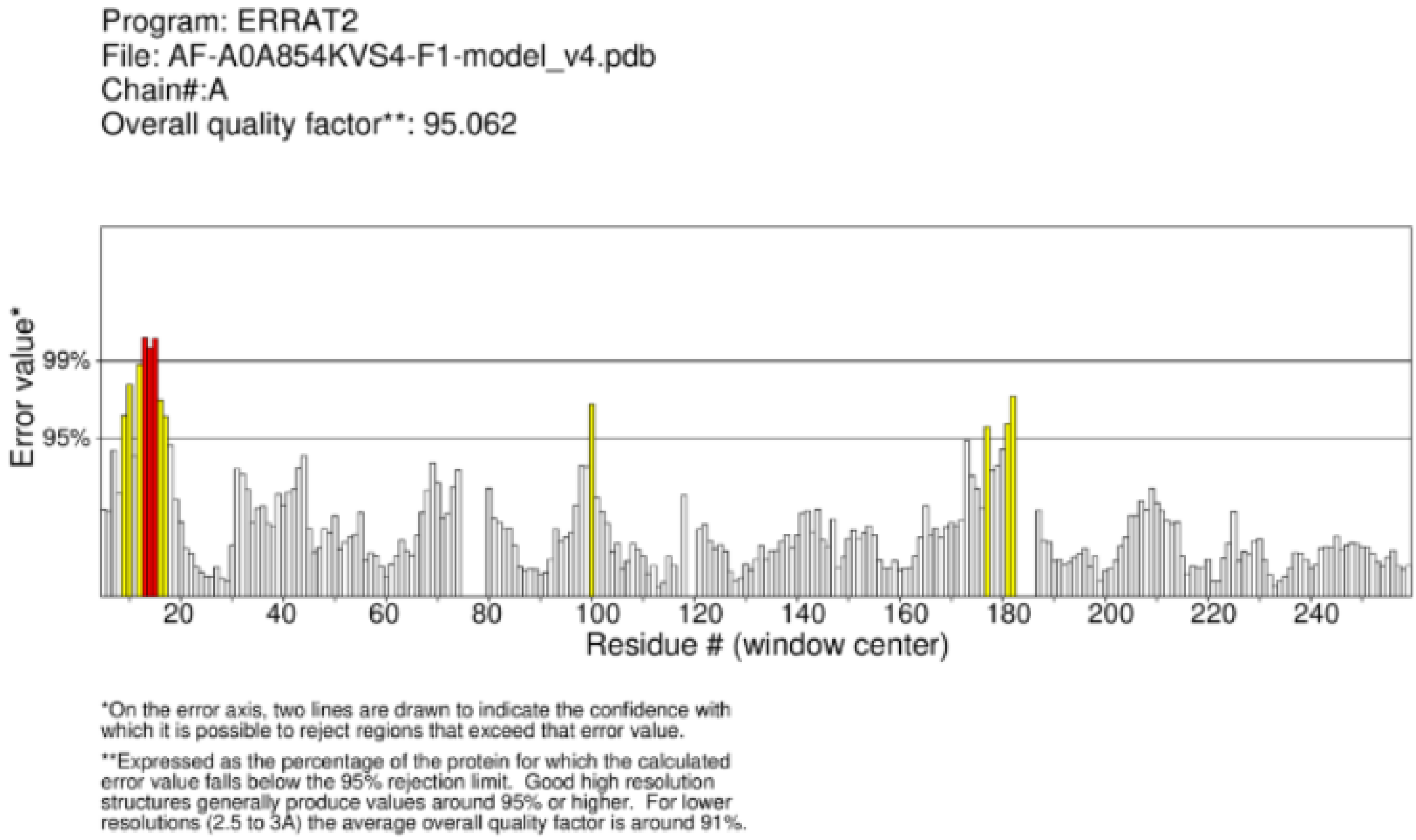

(b) VERIFY3D:

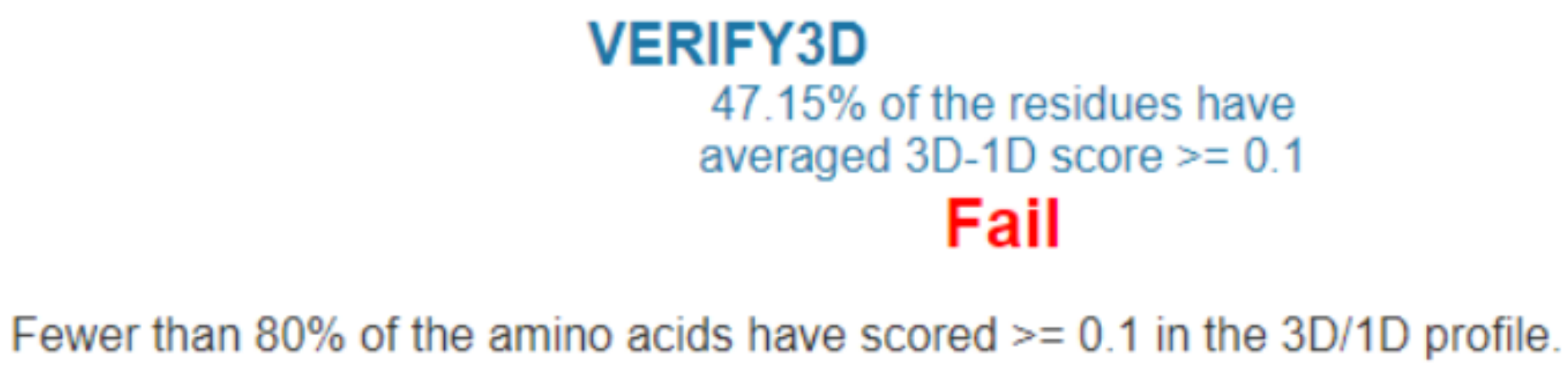

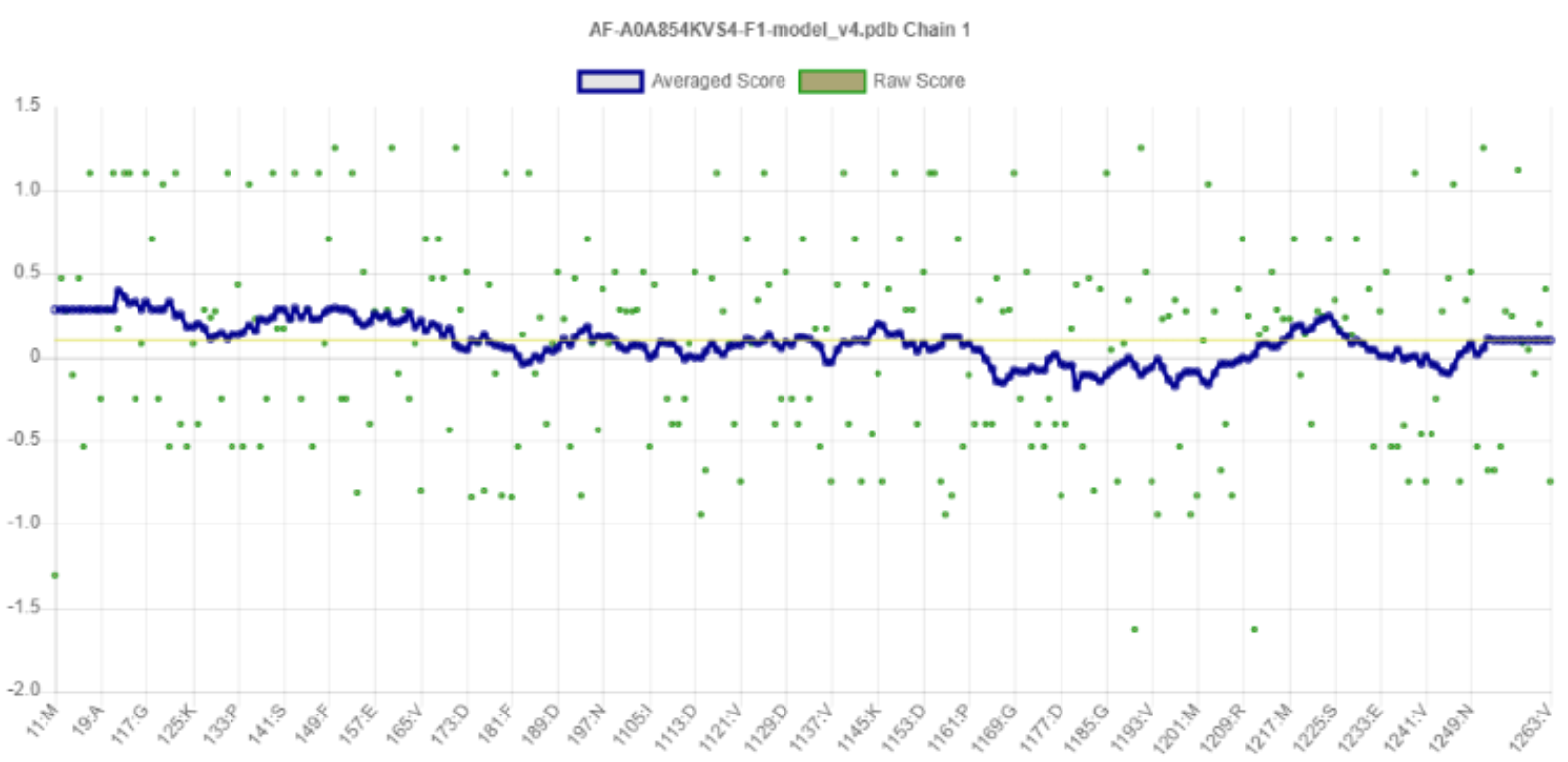

(c) PROCHECK:

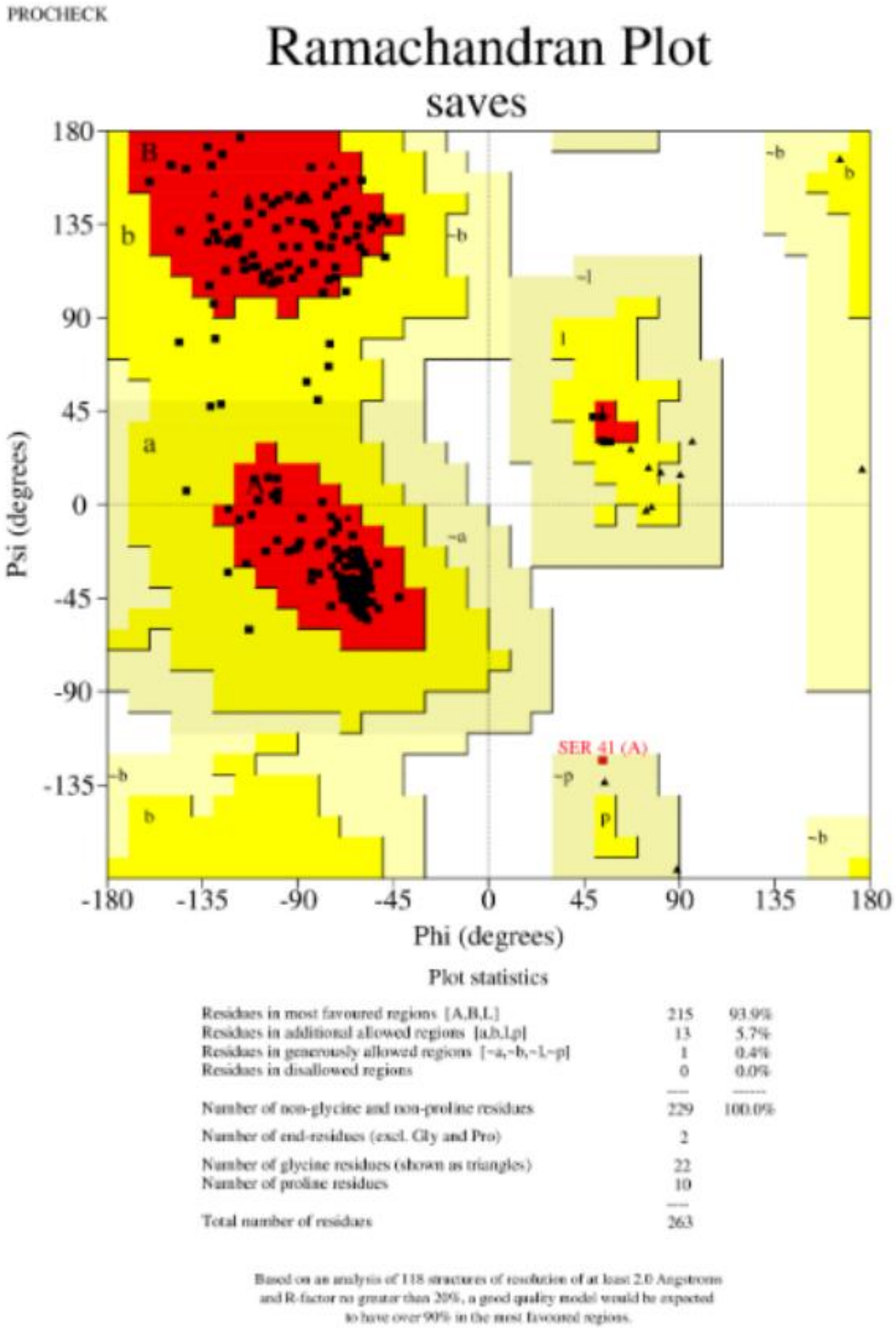

Fig. S18. Validation result of serine protease predicted by Sequence Similarity Search using AFDB:A0A854KVS4 as template. (a) ERRAT, (b)VERIFY3D, and (c) PROCHECK.

Table S1. The forward and reverse primer sequences for amplification of the serine protease gene.

Gene	Primer sequence	Length (bp)
Protease	Forward primer: 5'- GGTGGT**GGATCCG**ATGGATTATAATCGCGAT -3'	30
	Reverse primer: 5'- GGTGGT**AAGCTT**TTATTTCTTTAATCGATCCGC -3'	29

Table S2. Amino acid frequency analysis of serine protease sequences.

Species name	Ala	Cys	Asp	Glu	Phe	Gly	His	Ile	Lys	Leu	Met	Asn	Pro	Gln	Arg	Ser	Thr	Val	Trp	Tyr	Total
B.toyonensis HDX9609273	7.98	0.38	4.56	6.84	3.802	8.37	1.901	10.27	7.985	6.464	4.18	3.422	3.802	1.521	4.183	5.323	3.42	12.55	0.38	2.662	263
B.toyonensis WP220553642	7.98	0.76	4.56	6.84	3.802	8.37	1.901	10.27	7.605	6.464	4.18	3.422	3.802	1.901	4.183	5.323	3.42	12.55	0.38	2.281	263
B.cereus WP000662600	7.6	0.00	4.94	6.84	3.802	8.37	1.901	10.27	7.605	6.084	4.56	3.422	3.802	2.281	3.802	5.703	3.42	12.55	0.38	2.662	263
B.luti WP427229073	7.6	0.00	4.94	6.84	3.802	8.37	1.901	10.27	7.985	6.084	4.56	3.042	3.802	2.281	3.802	5.703	3.42	12.55	0.38	2.662	263
B.thuringiensis WP000662611	7.98	0.00	5.32	6.46	3.802	7.98	1.901	10.27	7.605	6.464	4.18	3.042	3.802	1.901	4.183	6.464	3.04	12.55	0.38	2.662	263
B.cereus HDR8489172	7.98	0.00	5.32	6.46	3.802	7.98	1.901	10.27	7.605	6.464	4.18	3.042	3.802	1.901	4.183	6.464	3.04	12.55	0.38	2.662	263
B.cereus EEK60889	8.37	0.00	5.32	6.46	3.802	7.98	1.901	10.65	7.605	6.464	4.18	3.042	3.802	1.901	4.183	6.084	3.04	12.17	0.38	2.662	263
B.cereus WP078403682	7.98	0.00	5.32	6.46	3.802	7.98	1.901	11.03	7.605	6.464	4.18	3.422	3.802	1.901	4.183	6.084	3.04	11.79	0.38	2.662	263
B.thuringiensis EAO53572	7.98	0.00	5.7	6.46	3.802	7.6	1.901	10.65	7.605	6.464	4.18	3.042	3.802	1.901	4.183	6.464	3.04	12.17	0.38	2.662	263
B.thuringiensis WP098222464	7.6	0.00	5.32	6.46	3.802	7.98	1.901	10.27	7.605	6.464	4.18	3.042	3.802	1.901	4.183	6.464	3.04	12.93	0.38	2.662	263
B.wiedmannii WP098093566	7.98	0.00	4.94	6.84	3.802	7.98	1.901	10.27	7.605	6.464	4.18	3.802	3.802	1.901	4.183	5.703	3.04	12.55	0.38	2.662	263
B.wiedmannii MCT6915489	7.6	0.00	4.56	7.22	3.802	7.98	1.901	10.27	7.605	6.464	4.18	3.802	3.802	1.901	4.183	6.084	3.04	12.55	0.38	2.662	263
B.mycoides EEL98227	8.37	0.38	4.94	6.84	3.802	7.98	2.281	12.17	7.605	6.464	3.8	2.662	3.802	1.901	4.183	5.703	3.04	11.03	0.38	2.662	263
B.cereus PFJ13601	8.37	0.38	4.56	7.22	3.802	8.37	1.901	11.41	7.224	6.084	3.8	3.042	3.802	1.901	4.563	5.323	2.66	12.55	0.38	2.662	263
B.sp MDJ1476587	7.98	0.00	4.56	7.22	3.802	7.98	1.901	10.65	7.605	6.464	4.18	3.802	3.802	1.901	4.183	5.703	3.04	12.17	0.38	2.662	263
B.mycoides WP137056832	8.75	0.38	4.94	6.84	3.802	7.98	2.281	12.17	7.605	6.464	3.8	2.662	3.802	1.901	4.183	5.323	3.04	11.03	0.38	2.662	263
B.hominis WP212934844	7.98	0.38	4.94	6.84	3.802	7.98	2.281	12.17	7.605	6.464	3.8	2.662	3.802	1.901	4.183	5.703	3.42	11.03	0.38	2.662	263
B.toyonensis AHA07180	7.98	0.38	4.56	6.84	3.802	8.37	1.901	10.27	7.605	6.464	4.18	3.422	3.802	1.901	4.183	5.323	3.42	12.55	0.38	2.662	263
Avg.	8.01	0.169	4.96	6.78	3.802	8.09	1.965	10.75	7.626	6.401	4.14	3.211	3.802	1.922	4.161	5.83	3.15	12.21	0.38	2.64	263

Table S3. Validation results of Serine Protease predicted by several web servers.

Tools	ERRAT	VERIFY3D	PROCHECK	Residue
Phyre2	89.627%	42.00%	88.5%	250
AlphaColab	96.761%	49.81%	93.0%	263
SWISS-MODEL (AlphaFold DB)	95.918%	48.67%	92.1%	263
Sequence Similarity Search (AlphaFold DB)				
(A) PNPLA domain-containing protein				
1. AFDB:J8KWL5 (*B. cereus*)	93.522%	49.43%	93.0%	263
2. AFDB:A0A2I0RDH6 (*B. cereus*)	95.510%	45.25%	93.4%	263
(B) Patatin family protein				
1. AFDB:A0A345XC57 (*Bacillus sp.)*	95.547%	46.77%	93.4%	263
2. AFDB:A0A7T3JG97 (*B. thuringiensis*)	95.082%	44.49%	94.3%	263
(C) Esterase				
1. AFDB:A0A242WPL1(*B. thuringiensis*)	94.694%	49.05%	92.6%	263
2. AFDB:A0A1V6LKE9(*B. toyonensis*)	95.529%	44.11%	93.0%	263
3. AFDB:A0A5K6LDT8(*B. sp.)*	94.694%	41.44%	93.9%	263
4. AFDB:A0A854KVS4(*B. thuringiensis*)	95.062%	47.15%	93.9%	263
